# Risk factors for saccular unruptured intracranial aneurysms: a systematic review and meta-analysis

**DOI:** 10.1093/esj/aakaf028

**Published:** 2026-02-09

**Authors:** Maaike J A van Eldik, Mariam Ali, Stijn Rietkerken, Jan W Schoones, Sanne A E Peters, Hester M den Ruijter, Ynte M Ruigrok

**Affiliations:** Department of Neurology and Neurosurgery, UMC Utrecht Brain Center, University Medical Center Utrecht, Utrecht University, Utrecht, The Netherlands; Department of Neurology, Leiden University Medical Center, Leiden, The Netherlands; Department of Neurology and Neurosurgery, UMC Utrecht Brain Center, University Medical Center Utrecht, Utrecht University, Utrecht, The Netherlands; Directorate of Research and Valorisation, Leiden University Medical Center, Leiden, The Netherlands; Julius Center for Health Sciences and Primary Care, University Medical Center Utrecht, Utrecht University, Utrecht, The Netherlands; The George Institute for Global Health, School of Public Health, Imperial College London, London, United Kingdom; Laboratory of Experimental Cardiology, University Medical Center Utrecht, Utrecht University, Utrecht, The Netherlands; Department of Neurology and Neurosurgery, UMC Utrecht Brain Center, University Medical Center Utrecht, Utrecht University, Utrecht, The Netherlands

**Keywords:** meta-analysis, risk factor, systematic review, unruptured intracranial aneurysm

## Abstract

**Introduction:**

Intracranial aneurysms are often unruptured and two-thirds of patients with unruptured intracranial aneurysms (UIAs) are women. Rupture of an intracranial aneurysm causes aneurysmal subarachnoid haemorrhage (aSAH). While risk factors for aSAH have been extensively studied, those for UIA remain less well understood. We performed a systematic review and meta-analysis to identify risk factors for the presence of saccular UIAs and assess potential sex differences.

**Patients and methods:**

We conducted a systematic review and meta-analysis of cohort, case–control, and cross-sectional studies on risk factors for UIA up to March 2024. Assessed risk factors included smoking, hypertension, alcohol use, diabetes, hypercholesterolaemia, physical activity, and body mass index. We performed random-effects meta-analyses to calculate pooled odds ratios (ORs) and 95% CIs for each risk factor.

**Results:**

We identified 21 studies reporting on overall 347 907 participants and 8698 UIA cases. Hypertension (OR 1.72, 95% CI, 1.42-2.09) and smoking (OR 1.47, 95% CI, 1.11-1.95) were associated with the presence of UIAs. No statistically significant associations were found for the other assessed risk factors. Among 18 studies that included both sexes, only one provided sex-stratified results, preventing us from assessing potential sex differences.

**Discussion:**

Future research should consistently report sex-stratified results to enable investigation of potential sex differences in UIA risk factors and further explore female-specific risk factors that may contribute to the high female preponderance in UIA.

**Conclusion:**

Hypertension and smoking are associated with an increased risk of UIAs. The lack of sex-stratified data limits conclusions about sex-specific risk profiles.

## Graphical Abstract

**Graphical Abstract:**
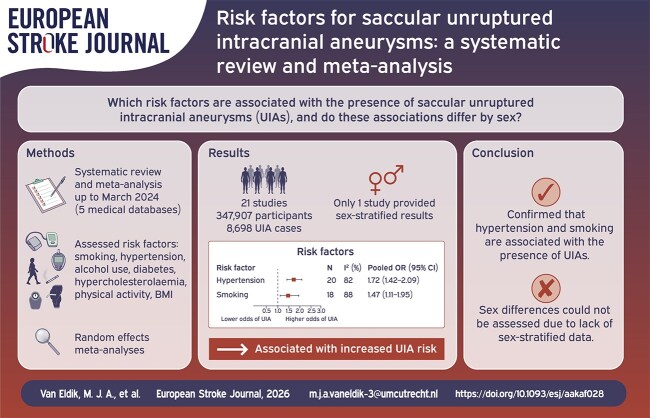

## Introduction

Unruptured intracranial aneurysms (UIAs) have a prevalence of approximately 3% in the general population.^1^ Rupture of an intracranial aneurysm causes aneurysmal subarachnoid haemorrhage (aSAH), a severe type of stroke with approximately one in three patients dying and one in three patients remaining dependent.^2^ Both UIA and aSAH are more common in women than in men, with overall two-thirds of patients being women.^1^^,^^3^ The reasons for this female preponderance remain unclear.

The risk of aSAH depends on both the development of UIAs and their subsequent rupture. It remains unclear whether risk factors for both stages overlap or whether distinct risk factors exist for each stage. It is therefore important to identify which risk factors contribute to UIA formation, as reducing the risk of developing UIAs represents a key strategy for lowering the incidence of aSAH. For aSAH, previous studies, including our recently updated meta-analysis, identified smoking, hypertension, and excessive alcohol use as the most important risk factors.^4,5^ In contrast, rigorous physical activity and diabetes were associated with a reduced risk of aSAH, while associations with hypercholesterolaemia and body mass index (BMI) remained inconclusive.^4^ However, research on similar modifiable risk factors for UIA is less extensive. To date, only hypertension and smoking are well-established, with limited evidence for other modifiable risk factors for UIA.^3,6-10^

Sex differences in the strength of certain risk factor associations may contribute to the higher burden of UIA and aSAH in women. In our recently updated meta-analysis, we confirmed that smoking was more strongly associated with aSAH in women than in men,^4^ but if sex differences exist for UIA is unknown.

Therefore, we performed a systematic review and meta-analysis to identify risk factors for the presence of saccular UIAs and to assess potential sex differences in these risk factor relations.

## Patients and methods

This systematic review and meta-analysis was conducted in accordance with the Preferred Reporting Items for Systematic Reviews and Meta-analyses Statement guidelines and followed a prospectively registered protocol in PROSPERO (CRD42024529043).^11^

### Data sources and search strategy

We performed a systematic search of the databases PubMed, Embase, Web of Science, Cochrane Library, and Emcare from database inception until March 2024 using search terms related to (1) UIAs, (2) predefined specific risk factors, and (3) cohort, case–control, and cross-sectional studies. Concurrently, we conducted a separate systematic review and meta-analysis on risk factors for aSAH.^8^ Given that both reviews examined the same preselected risk factors, we used a unified search strategy and conducted the screening process jointly. The search excluded reviews, case-reports, and non-English publications. A medical information specialist with expertise in systematic reviews was consulted for help in developing the search strategy. In addition, we screened the reference lists of the included articles to identify any additional eligible studies. The full search strategy is provided in Supplementary Material S1.

### Selection criteria

Inclusion criteria were: (1) cohort, case–control, or cross-sectional studies reporting on risk factors associated with the presence of saccular UIAs, with no minimum sample size restriction; (2) provision of crude or adjusted effect estimates with 95% CIs for smoking, hypertension, alcohol use, diabetes, hypercholesterolaemia, rigorous physical activity, and BMI, or data allowing for their calculation; (3) confirmation of UIA presence by neuroimaging (computed tomography angiography, magnetic resonance angiography, or conventional angiography), autopsy or International Classification of Diseases codes; (4) patients aged ≥18 years; and (5) articles in English. In case of multiple publications originating from the same study population, we included data from the most recent publication.

We excluded studies (1) that analysed UIA and aSAH cases together without separate results for UIA; and (2) that focused on specific patient subgroups, such as young patients (defined as age <50 years), those with familial, fusiform, or multiple aneurysms, a specific aneurysm location or size, and those focused on UIAs related to Marfan syndrome, Loeys-Dietz syndrome, autosomal dominant polycystic kidney disease, fibromuscular dysplasia, and Ehlers-Danlos syndrome (more details available in Supplementary Material S2).

### Data extraction and quality assessment

To determine study eligibility, two reviewers (M.J.A.v.E. and M.A.) independently screened titles and abstracts using the predefined in- and exclusion criteria. Subsequently, one reviewer (M.J.A.v.E.) screened full-text versions of potentially relevant articles and performed the data extraction and quality assessment of studies meeting the inclusion criteria. A second reviewer (S.R.) independently verified 50% of the full-text screenings, data extraction forms, and quality assessments, based on a predefined protocol. Data were extracted using a standardised data extraction form (more details available in Supplementary Material S3).

For quality assessment, we used an adapted version of the Newcastle-Ottawa Scale.^12^ Each study was rated as having low, high, or unclear risk of bias across the following domains (1) validation of diagnosis, (2) assessment of risk factors, (3) adjustment for confounding, and (4) generalizability (Supplementary Material S4). Disagreements at any stage, including title and abstract screening, duplicate full-text screening, data extraction, or quality assessment, were resolved through discussion with a third reviewer (Y.M.R.).

### Definition and classification of risk factors

To ensure comparability of data across studies, we standardised risk factor definitions wherever possible. We categorised smoking into (1) ever smokers and (2) never smokers. Alcohol use was categorised both as (1) alcohol use vs. no alcohol use, and (2) regular use vs. non-regular use. Lipid levels often lacked precise definitions and were reported as either hypercholesterolaemia or hyperlipidaemia (the latter being a broader term that includes both cholesterol and triglycerides). Therefore, we first combined all lipid-related definitions into a single group (any lipid abnormality vs. normal lipids), and then further categorised them into (1) hypercholesterolaemia (yes vs. no), and (2) hyperlipidaemia (yes vs. no). Rigorous physical activity was categorised as (1) ≥3 times a week, and (2) <3 times a week, and BMI as (1) ≥30 kg/m^2^ vs. (2) <30 kg/m^2^. Definitions for the remaining risk factors, hypertension, and diabetes mellitus, were dichotomised as present vs. absent and followed the criteria used in the original publications, consistent with a 2005 meta-analysis on risk factors for aSAH.^5^ Studies using risk factor definitions that could not be harmonised with the above categories, were included in the systematic review, but excluded from the meta-analysis.^6,13-18^ Specifically, 2 studies were excluded from the smoking meta-analysis,^6,13^ 1 study from alcohol use,^17^ 5 from BMI,^13-17^ 1 from hyperlipidaemia,^18^ and 1 from rigorous physical activity^17^ (studies are described in Supplementary Material S5).

### Statistical analysis

Pooled proportions of women among participants and UIA cases were calculated using a random effects meta-analysis, accounting for the weighted contribution of each study. We pooled data for each individual risk factor using an inverse variance weighted random effects meta-analysis. Data from case–control and cohort studies were combined. For each risk factor, we used the most adjusted effect estimates available and also included crude estimates if no adjusted estimates were available. We pooled different measures of association and interpreted them as equivalent to odds ratios (ORs).

We quantified heterogeneity between studies using the *I*^2^ statistic, as proposed by Higgins, and considered *I*^2^ of 25%–50% as moderate, 50%–75% as substantial, and >75% as considerable heterogeneity.^19^ When more than 10 studies reported on a specific risk factor, potential publication bias was assessed using funnel plots for visual inspection and Egger’s regression tests to statistically evaluate small-study effects. We planned to perform sex-stratified analyses when sufficient data were available. Additionally, we performed subgroup analyses for each risk factor, restricting to studies that reported adjusted effect estimates (ie, those that had adjusted for at least one covariate). All statistical analyses were performed using R statistical software, version 4.0.2.

## Results

**Figure 1:**
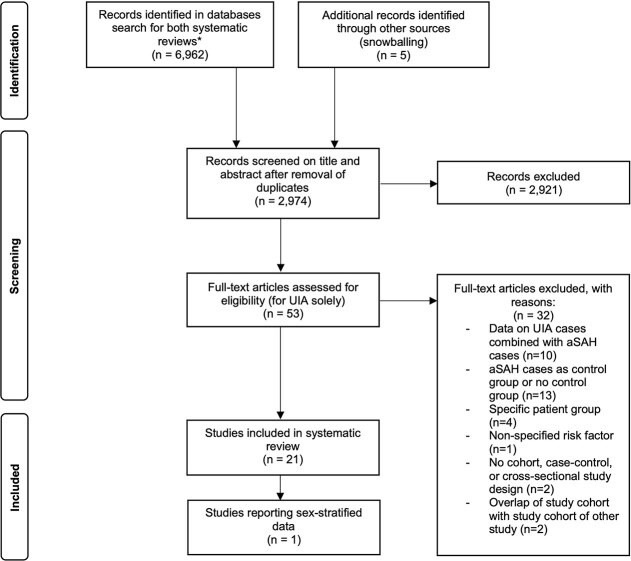
PRISMA flow diagram depicting study selection *The combined search identified 6 962 records, including records for a systematic review and meta-analysis on risk factors for aneurysmal subarachnoid haemorrhage. Abbreviations: UIA = unruptured intracranial aneurysms; aSAH = aneurysmal subarachnoid haemorrhage; PRISMA = Preferred Reporting Items for Systematic Reviews and Meta-Analyses.

The systematic search identified a total of 2 969 articles. After title and abstract screening, 53 articles reporting on UIA were evaluated in full-text and of those, a total of 21 studies met the inclusion criteria (Figure 1).^6,8,10,13-18,20-31^ Details of studies excluded after full-text review are provided in Supplementary Material S6. These 21 studies included a total of 347 907 participants (range 132–131,999) and 8 698 UIA cases (range 25–5335). The pooled proportion of women was 55.2% (95% CI, 48.9–61.4) among all participants and 62.8% (95% CI, 58.6–66.8) among UIA cases. The included studies comprised 11 case–control,^6,8,13,14,18,20,22,25,27,28,31^ 7 cross-sectional,^15-17,21,23,26,29^ 2 prospective cohort studies,^10,24^ and 1 retrospective cohort study.^30^ General characteristics of the study populations are summarised in Table 1. The age of participants ranged from 18 to 91 years. Geographically, the studies originated from 13 Asian,^8,13,15,16,18,20-24,26,29,31^ 4 North-American,^14,25,27,28^ and 4 European countries.^6,10,17,30^ Hypertension (*n* = 20), smoking (*n* = 18), and diabetes (*n* = 16) were the most frequently reported risk factors. Alcohol use and lipid abnormalities were assessed in 11 and 13 studies, respectively, while only 2 studies each examined BMI and rigorous physical activity. Eighteen studies included both women and men, while 3 studies were conducted in female-only cohorts.^14,27,28^ One out of these 18 studies provided sex-stratified results, precluding the possibility of assessing sex differences in risk factors for UIA.^8^ This 1 study reported that hypertension and diabetes were similarly and significantly associated with the presence of UIAs in both women and men.

**Table 1:** Characteristics of included studies.

**Author, year of publication and country**	**Study design**	**Size study population, *n* (% women)**	**Age range (year)**	**Number of UIA cases (% women)**	**Study period**	**Diagnostic method**	**Risk factors assessed (and included in meta-analysis)**
Horikoshi 2002, Japan	Case–control	381 (unclear)	44–85	127 (66)	1997—2000	MRA, CTA, DSA	Hypertension, hypercholesterolaemia
Inagawa 2010, Japan	Case–control	1064 (65)	34–88	266 (65)	1981—2005	MRA, CTA, angiography	Hypertension, smoking, diabetes, alcohol use, hypercholesterolaemia
Chen 2011, USA	Case–control	459 (100)	31–80	39 (100)	2008—2010 (questionnaires 1994-1998)	“brain vascular imaging”	Smoking
Vlak 2013, The Netherlands	Case–control	780 (69)	Mean age 55	206 (67)	2006—2010	CTA, MRA or conventional angiography	Hypertension, diabetes, alcohol use, hypercholesterolaemia, BMI, physical activity
Li 2013, China	Cross-sectional	4813 (51)	35–75	366 (56)	2007–2011	MRA	Hypertension, smoking, diabetes, alcohol use
Jing Li 2014, China	Cross-sectional	3993 (46)	20–80	350 (57)	2009–2010	3D CE-MRA	Hypertension, smoking, diabetes, alcohol use, hyperlipidaemia
Matsukawa 2014, China	Case–control	132 (67)	Mean age 62	66 (67)	2013–2014	MRA, CTA, DSA	Hypertension, diabetes, alcohol use, hypercholesterolaemia
Zhang 2015, China	Case–control	294 (46)	Mean age 51	37 (49)	Unclear	CTA, DSA	Hypertension, smoking, diabetes, alcohol use, hypercholesterolaemia
Kang 2015, South-Korea	Cross-sectional	18 954 (39)	Mean age 57	367 (51)	2004–2014	MRA	Hypertension, smoking, diabetes, alcohol use
Kim Tackeun 2016, Korea	Prospective cohort study	131 999 (41)	Unclear	491 (59)	2005–2013	ICD-10 code I67.1	Hypertension, smoking, diabetes, BMI, physical activity, alcohol use, hypercholesterolaemia
Atchaneeyasakul 2018, USA	Case–control	486 (53)	Mean age 61	243 (71)	2004–2014	DSA	Hypertension, smoking, diabetes, alcohol use, hyperlipidaemia
Imaizumi 2018, Japan	Cross-sectional	4070 (42)	Mean age 51	176 (60)	2014–2015	MRI/MRA	Hypertension, smoking, diabetes, hyperlipidaemia
Yoon 2019, South-Korea	Case–control	238 (32)	18+, mean age 62	25 (36)	2014–2016	MRA or CTA	Hypertension, smoking, diabetes
Müller 2019, Norway	Prospective cohort study	83 710 (53)	Unclear	92 (75)	1999–2014	MRA, CTA, DSA	Hypertension, smoking
Cras 2020, The Netherlands	Cross-sectional	5841 (55)	Mean age 64	134 (68)	2005–2015	MRI	Hypertension, smoking, diabetes, hypercholesterolaemia
Ogilvy 2020, USA (single centre)	Case–control	194 (100)	30–60	64 (100)	2016–2018	MRA	Hypertension, smoking, diabetes
Ogilvy 2020, USA and Canada (multicentre)	Case–control	226 (100)	30–60	113 (100)	2016–2018	MRA	Hypertension, smoking, diabetes
Kim Jae Ho 2021, Korea	Cross-sectional	2118 (44)	Mean age 54	80 (55)	2011–2012	MRA	Hypertension, smoking, diabetes, hyperlipidaemia
Igase 2021, Japan	Cross-sectional	1376 (59)	31–91	79 (70)	2006–2013	MRA	Hypertension, smoking, diabetes
Räisänen 2022, Finland	Retrospective cohort study	1419 (50)	Median age 46	42 (50)	1975–2014	MRA, CTA, or 4-vessel DSA	Hypertension, smoking, alcohol use
Park 2023, Korea	Case–control	85 360 (68)	Mean age 60	5335 (68)	2003–2019	ICD-10 code I67.1	Hypertension, smoking, alcohol use

Studies are shown in ascending chronological order. Abbreviations: BMI = body mass index; CE-MRA = contrast-enhanced magnetic resonance angiography; CTA = computed tomography angiography; DSA = digital subtraction angiography; ICD = International Classification of Diseases; MRI = magnetic resonance imaging; MRA = magnetic resonance angiography.

### Quality assessment

The overall quality of included studies varied (Supplementary Material S7). Two of the 21 studies were scored as having low risk of bias across all 4 assessed domains, 2 were scored as high or unclear risk in 1 domain, and the remaining 17 studies were scored as high or unclear risk in 2 or more of the 4 domains. The most common sources of bias were limited generalizability, often due to use of hospital-based populations, and insufficient adjustment for age as a confounding factor. Funnel plots were produced for smoking, hypertension, diabetes, and any lipid abnormality. Visual assessment of these funnel plots showed no clear asymmetry, and Egger’s regression tests did not indicate small-study effects (all *P* > .25), together suggesting low risk of publication bias (Supplementary Material S8).

### Meta-analysis

**Figure 2:**
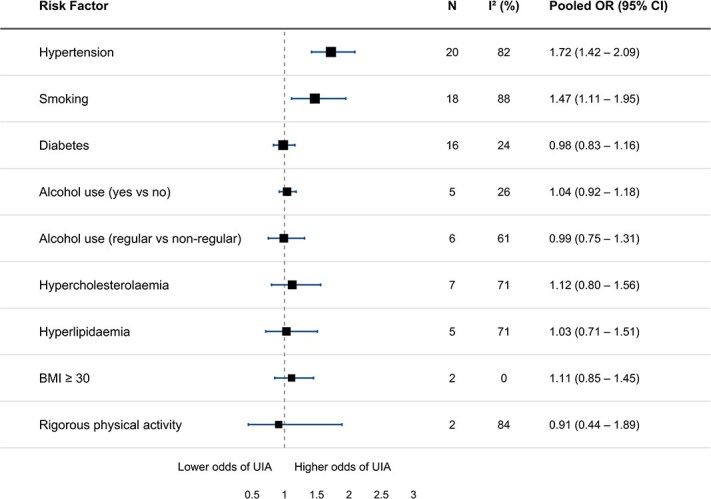
Summary of forest plots for each risk factor. Abbreviations: BMI = body mass index; N = number of studies; OR = odds ratio.

Forest plots for each individual risk factor are provided in Supplementary Material S9–S18. A combined forest plot summarising the pooled effect estimates of the assessed risk factors is shown in Figure 2. Hypertension and a history of ever smoking were associated with the presence of UIA, with a pooled OR of 1.72 (95% CI, 1.42–2.09, *I*^2^ = 82%, 20 studies) for hypertension and a pooled OR of 1.47 (95% CI, 1.11–1.95, *I*^2^ = 88%, 18 studies) for ever smoking. In subgroup analyses including only studies that adjusted their effect estimates for at least one covariate (mostly sex or age), hypertension (OR 1.92, 95% CI, 1.45–2.54, *I*^2^ = 76%, 13 studies), and ever smoking (OR 2.28, 95% CI, 1.70–3.06, *I*^2^ = 68%, 9 studies) remained statistically significantly associated with an increased UIA risk (Supplementary Materials S19 and S20).

No statistically significant associations were found between UIA and diabetes (pooled OR 0.98, 95% CI, 0.83–1.16, *I*^2^ = 24%, 16 studies), regular alcohol use (pooled OR 0.99, 95% CI, 0.75–1.31, *I*^2^ = 61%, 6 studies), BMI ≥30 (pooled OR 1.11, 95% CI, 0.85–1.45, *I*^2^ = 0%, 2 studies), or rigorous physical activity (pooled OR 0.91, 95% CI, 0.44–1.89, *I*^2^ = 84%, 2 studies). Likewise, any lipid abnormality (combined group of all reported lipid-related abnormalities) (pooled OR 1.19, 95% CI, 0.98–1.45, *I*^2^ = 55%, 13 studies), or hypercholesterolaemia specifically (pooled OR 1.12, 95% CI, 0.80–1.56, *I*^2^ = 71%, 7 studies) were not statistically significantly associated with UIA. Subgroup analyses for diabetes and hypercholesterolaemia yielded similar findings (Supplemental Materials S21 and S22).

## Discussion

In this systematic review and meta-analysis aimed at identifying risk factors for UIAs, we found that both hypertension and a history of smoking were associated with the presence of UIAs. No statistically significant associations were observed for the other assessed risk factors. Although one of the objectives was to assess sex differences in risk factor associations, this was not feasible, as only one of the 18 studies that included both sexes provided sex-stratified results.^8^ This study reported similar associations between hypertension and diabetes with UIAs in both sexes.

Our meta-analysis provides the most up-to-date and precise estimates of the associations between hypertension, smoking, and the presence of UIAs, confirming these as key modifiable risk factors. Similarly, our recent meta-analysis on risk factors for aSAH, which assessed the same risk factors as our study, identified hypertension and smoking as the most important risk factors for aSAH, along with alcohol consumption.^4^ These findings support the hypothesis that key modifiable risk factors may play roles in both the formation and rupture of intracranial aneurysms. While we found no statistically significant associations between UIAs and other modifiable risk factors, the aSAH meta-analysis additionally reported diabetes and physical activity to be associated with decreased aSAH risk, with inconsistent associations for hypercholesterolaemia and BMI.^4^ Variations in risk factor definitions and the limited numbers of studies for these risk factors may partly explain why these associations were not observed in the context of UIAs. Alternatively, some of these risk factors may primarily influence the risk of aneurysm rupture rather than UIA formation, highlighting potential differences in the risk factor profiles underlying UIA development versus aSAH occurrence.

Due to lack of sex-stratified data in our included studies, as we previously emphasised,^32^ we were unable to assess whether the found associations of hypertension and smoking with UIA differ between women and men. Therefore, we could not assess whether sex differences in the strength of these risk factors’ associations might contribute to the higher burden in women.

In this study, we focused on risk factors that occur in both women and men. However, given the female preponderance in UIA, it is important to also consider the role of female-specific risk factors. In our recent aSAH meta-analysis, no statistically significant associations were found between hormone replacement therapy or oral contraceptive use and aSAH.^4^ As part of the current UIA systematic search, we also screened for studies reporting on these two risk factors, but only one matched case–control study was identified. This study reported ORs of 3.10 (95% CI 1.50-6.20) for hormone replacement therapy use and 2.10 (95% CI 1.20-3.80) for oral contraceptive use, using patients with UIAs as the reference group, suggesting a lower likelihood of UIA among users compared with non-users.^14^ As evidence is limited to this single study, robust conclusions cannot be drawn. Other female-specific risk factors, such as pregnancy-related factors, remain largely unexplored. Further large-scale studies are needed to clarify the role of hormonal and reproductive factors in UIA risk, which may help explain the persistent female preponderance.

A strength of our study is that it included assessments of a wide range of risk factors, encompassing 347 907 participants and 8698 UIA cases. Furthermore, we provided an extensive risk of bias assessment with additional funnel plots to evaluate the quality of the included studies and to assess publication bias, thereby strengthening the interpretation of our findings. Our study also has several limitations. First, we observed considerable heterogeneity across the included studies in terms of study design, population characteristics, and risk factor definitions. Although we standardised risk factor definitions where possible to enable the meta-analysis, the underlying heterogeneity remains a limitation. The variability in definitions of risk factors also necessitated dichotomisation of most risk factors, which limits our ability to assess thresholds of exposure. Secondly, the overall quality of the included studies was limited; with only two of the 21 studies rated as low risk of bias in our quality assessment. This precluded a subgroup analysis based solely on high-quality studies. However, we did perform a sensitivity analysis restricted to studies that adjusted their effect estimates for at least one covariate, which partially addressed potential confounding. Still, substantial heterogeneity remained. This likely reflects both incomplete adjustment for confounding, since a substantial number of studies did not adjust for age and the covariates included in adjustments varied widely, as well as differences in study populations and methodological approaches. Third, we analysed risk factors independently and did not assess their joint or synergistic effects. However, some patients may have had multiple risk factors, and prior research has shown that the combined effect of hypertension and smoking exceeds the sum of their individual risks.^6^ As a result, our independent assessments may underestimate the true effect sizes.

In summary, we reaffirm that hypertension and smoking are associated with the presence of UIAs. These risk factors were previously established as being associated with aneurysm rupture, and our study now also confirms their role in UIA occurrence. These modifiable risk factors should be actively managed in patients at high risk of developing UIAs, as well as in those already diagnosed with a UIA, to potentially reduce the risk of aneurysm formation and rupture. Future research should not only focus on stratifying results by sex, but also on the investigation into female-specific risk factors, such as hormonal and pregnancy-related factors. A deeper understanding of the female predisposition to UIA is essential to optimise both prevention strategies and clinical management.

## Standard protocol approvals, registrations, and patient consents

Review board approval and informed consent were not required because this research used only published, deidentified data.

## Supplementary Material

Clean_Revised_Supplemental_material_ESJ_aakaf028

## Data Availability

Data not published within the article are available from the corresponding author on reasonable request. The data supplement is available online.
