# Supplementary material for: Risk factors for saccular unruptured intracranial aneurysms: a systematic review and meta-analysis
Source: Eur Stroke J. 2026 Feb 9;11(2):aakaf028. doi: 10.1093/esj/aakaf028 (PMC12884560; doi:10.1093/esj/aakaf028)
Supplement: Clean_Revised_Supplemental_material_ESJ_aakaf028 [file clean_revised_supplemental_material_esj_aakaf028.docx]

**SUPPLEMENTAL MATERIAL**

**Risk Factors for Saccular Unruptured Intracranial Aneurysms:**

**A Systematic Review and Meta-analysis**

S1. Detailed search query

S2. Selection criteria

S3. Data extraction

S4. Protocol - Risk of Bias Assessment (Customized version of the Newcastle-Ottawa Scale)

S5. Unclassifiable risk factor definitions not included in meta-analysis

S6. List of studies excluded after full-text review

S7. Risk of Bias Assessment

S8. Publication bias funnel plots

S9. Forest plot of hypertension (present vs absent)

S10. Forest plot of smoking (ever vs never)

S11. Forest plot of diabetes (present vs absent)

S12. Forest plot of alcohol use (yes vs no)

S13. Forest plot of alcohol use (regular vs non-regular)

S14. Forest plot of any lipid abnormality (normal vs any lipid abnormality)

S15. Forest plot of hypercholesterolemia (hypercholesterolemia vs normal cholesterol)

S16. Forest plot of hyperlipidemia (hyperlipidemia vs normal lipids)

S17. Forest plot of body mass index (BMI) (≥ 30 kg/m^2^ vs < 30 kg/m^2^)

S18. Forest plot of physical activity (≥ 3 times a week vs < 3 times a week)

S19. Subgroup analysis – Forest plot of hypertension (present vs absent)

S20. Subgroup analysis – Forest plot of smoking (ever vs never)

S21. Subgroup analysis – Forest plot of diabetes (present vs absent)

S22. Subgroup analysis – Forest plot of hypercholesterolemia (hypercholesterolemia vs normal cholesterol)

S23. References

**S1. Detailed search query**

**I. PubMed**

*(Final search run on 01-03-2024; 2204 items)*

(((("Intracranial Aneurysm"[mesh] OR "Intracranial Aneurysm"[tw] OR "Intracranial Aneurysms"[tw] OR "cerebral aneurysm"[tw] OR "cerebral aneurysms"[tw] OR "Brain Aneurysm"[tw] OR "Brain Aneurysms"[tw] OR "Berry Aneurysm"[tw] OR "Berry Aneurysms"[tw] OR "Saccular Aneurysm"[tw] OR "Saccular Aneurysms"[tw] OR "Intracranial Aneurysm"[title/abstract:~3] OR "Intracranial Aneurysms"[title/abstract:~3] OR "cerebral aneurysm"[title/abstract:~3] OR "cerebral aneurysms"[title/abstract:~3] OR "brain aneurysm"[title/abstract:~3] OR "brain aneurysms"[title/abstract:~3] OR "berry aneurysm"[title/abstract:~3] OR "berry aneurysms"[title/abstract:~3] OR "saccular aneurysm"[title/abstract:~3] OR "saccular aneurysms"[title/abstract:~3]) AND ("Smoking"[majr] OR "smoking"[ti] OR "smoke"[ti] OR "smoked"[ti] OR "Nicotine"[majr] OR "Nicotine"[ti] OR "Tobacco Products"[majr] OR "Tobacco"[ti] OR "Hypertension"[majr] OR "hypertension"[ti] OR "hypertens*"[ti] OR "High Blood Pressure"[ti] OR "elevated blood pressure"[ti] OR "Alcoholism"[majr] OR "Alcoholism"[ti] OR "alcohol abuse"[ti] OR "Alcohol Dependence"[ti] OR "Alcohol Addiction"[ti] OR "Alcoholic Intoxication"[ti] OR "Ethanol Abuse"[ti] OR "Alcohol Use Disorder"[ti] OR "Alcohol Use Disorders"[ti] OR "Contraceptives, Oral"[majr] OR "oral contraception"[ti] OR "Oral Contraceptives"[ti] OR "Oral Contraceptive"[ti] OR "Contraceptive Agents, Hormonal"[majr] OR "Contraceptive Agents, Hormonal"[Pharmacological Action] OR "hormonal contraceptive"[ti] OR "hormonal contraceptives"[ti] OR "Hormone Replacement Therapy"[majr] OR "hormone replacement therapy"[ti] OR "Estrogen Replacement Therapy"[ti] OR "Oestrogen Replacement Therapy"[ti] OR "hormone replacement"[ti] OR "Estrogen Replacement"[ti] OR "Oestrogen Replacement"[ti] OR "hormone therapy"[ti] OR "hormone therapeutic"[ti] OR "hormone treatment"[ti] OR "hormone substitution"[ti] OR "Ethnicity"[majr] OR "ethnicity"[ti] OR "ethnic"[ti] OR "Hypercholesterolemia"[majr] OR "Hypercholesterolemia"[ti] OR "Hypercholesterolem*"[ti] OR "hypercholesterolaemia"[ti] OR "hypercholesterolaem*"[ti] OR "High Cholesterol"[ti] OR "Elevated Cholesterol"[ti] OR "Exercise"[majr] OR "rigorous physical activity"[ti] OR "physical activity"[ti] OR "Exercise Training"[ti] OR "Physical Activities"[ti] OR "Physical Exercise"[ti] OR "Physical Exercises"[ti] OR "lean BMI"[ti] OR ("Body Mass Index"[majr] AND "lean"[ti]) OR "lean Body Mass Index"[ti] OR "Diabetes Mellitus"[majr] OR "Diabetes Mellitus"[ti] OR "diabetes"[ti] OR "diabetic"[ti] OR "diabetics"[ti])) OR (("Intracranial Aneurysm"[majr] OR "Intracranial Aneurysm"[ti] OR "Intracranial Aneurysms"[ti] OR "cerebral aneurysm"[ti] OR "cerebral aneurysms"[ti] OR "Brain Aneurysm"[ti] OR "Brain Aneurysms"[ti] OR "Berry Aneurysm"[ti] OR "Berry Aneurysms"[ti] OR "Saccular Aneurysm"[ti] OR "Saccular Aneurysms"[ti] OR "Intracranial Aneurysm"[title:~3] OR "Intracranial Aneurysms"[title:~3] OR "cerebral aneurysm"[title:~3] OR "cerebral aneurysms"[title:~3] OR "brain aneurysm"[title:~3] OR "brain aneurysms"[title:~3] OR "berry aneurysm"[title:~3] OR "berry aneurysms"[title:~3] OR "saccular aneurysm"[title:~3] OR "saccular aneurysms"[title:~3]) AND ("Smoking"[Mesh] OR "smoking"[tw] OR "smoke"[tw] OR "smoked"[tw] OR "Nicotine"[mesh] OR "Nicotine"[tw] OR "Tobacco Products"[mesh] OR "Tobacco"[tw] OR "Hypertension"[Mesh] OR "hypertension"[tw] OR "hypertens*"[tw] OR "High Blood Pressure"[tw] OR "elevated blood pressure"[tw] OR "Alcoholism"[Mesh] OR "Alcoholism"[tw] OR "alcohol abuse"[tw] OR "Alcohol Dependence"[tw] OR "Alcohol Addiction"[tw] OR "Alcoholic Intoxication"[tw] OR "Ethanol Abuse"[tw] OR "Alcohol Use Disorder"[tw] OR "Alcohol Use Disorders"[tw] OR "Contraceptives, Oral"[Mesh] OR "oral contraception"[tw] OR "Oral Contraceptives"[tw] OR "Oral Contraceptive"[tw] OR "Contraceptive Agents, Hormonal"[mesh] OR "Contraceptive Agents, Hormonal"[Pharmacological Action] OR "hormonal contraceptive"[tw] OR "hormonal contraceptives"[tw] OR "Hormone Replacement Therapy"[Mesh] OR "hormone replacement therapy"[tw] OR "Estrogen Replacement Therapy"[tw] OR "Oestrogen Replacement Therapy"[tw] OR "hormone replacement"[tw] OR "Estrogen Replacement"[tw] OR "Oestrogen Replacement"[tw] OR "hormone therapy"[tw] OR "hormone therapeutic"[tw] OR "hormone treatment"[tw] OR "hormone substitution"[tw] OR "Ethnicity"[Mesh] OR "ethnicity"[tw] OR "ethnic"[tw] OR "Hypercholesterolemia"[Mesh] OR "Hypercholesterolemia"[tw] OR "Hypercholesterolem*"[tw] OR "hypercholesterolaemia"[tw] OR "hypercholesterolaem*"[tw] OR "High Cholesterol"[tw] OR "Elevated Cholesterol"[tw] OR "Exercise"[Mesh] OR "rigorous physical activity"[tw] OR "physical activity"[tw] OR "Exercise Training"[tw] OR "Physical Activities"[tw] OR "Physical Exercise"[tw] OR "Physical Exercises"[tw] OR "lean BMI"[tw] OR ("Body Mass Index"[Mesh] AND "lean"[tw]) OR "lean Body Mass Index"[tw] OR "Diabetes Mellitus"[Mesh] OR "Diabetes Mellitus"[tw] OR "diabetes"[tw] OR "diabetic"[tw] OR "diabetics"[tw])) OR (("Intracranial Aneurysm"[mesh] OR "Intracranial Aneurysm"[tw] OR "Intracranial Aneurysms"[tw] OR "cerebral aneurysm"[tw] OR "cerebral aneurysms"[tw] OR "Brain Aneurysm"[tw] OR "Brain Aneurysms"[tw] OR "Berry Aneurysm"[tw] OR "Berry Aneurysms"[tw] OR "Saccular Aneurysm"[tw] OR "Saccular Aneurysms"[tw] OR "Intracranial Aneurysm"[title/abstract:~3] OR "Intracranial Aneurysms"[title/abstract:~3] OR "cerebral aneurysm"[title/abstract:~3] OR "cerebral aneurysms"[title/abstract:~3] OR "brain aneurysm"[title/abstract:~3] OR "brain aneurysms"[title/abstract:~3] OR "berry aneurysm"[title/abstract:~3] OR "berry aneurysms"[title/abstract:~3] OR "saccular aneurysm"[title/abstract:~3] OR "saccular aneurysms"[title/abstract:~3]) AND ("unruptured"[tw] OR "un ruptured"[tw] OR "unruptur*"[tw] OR "un ruptur*"[tw] OR "incidental"[tw] OR "incidental*"[tw]) AND ("Smoking"[mesh] OR "smoking"[tw] OR "smoke"[tw] OR "smoked"[tw] OR "Nicotine"[mesh] OR "Nicotine"[tw] OR "Tobacco Products"[mesh] OR "Tobacco"[tw] OR "Hypertension"[mesh] OR "hypertension"[tw] OR "hypertens*"[tw] OR "High Blood Pressure"[tw] OR "elevated blood pressure"[tw] OR "Alcoholism"[mesh] OR "Alcoholism"[tw] OR "alcohol abuse"[tw] OR "Alcohol Dependence"[tw] OR "Alcohol Addiction"[tw] OR "Alcoholic Intoxication"[tw] OR "Ethanol Abuse"[tw] OR "Alcohol Use Disorder"[tw] OR "Alcohol Use Disorders"[tw] OR "Contraceptives, Oral"[mesh] OR "oral contraception"[tw] OR "Oral Contraceptives"[tw] OR "Oral Contraceptive"[tw] OR "Contraceptive Agents, Hormonal"[mesh] OR "Contraceptive Agents, Hormonal"[Pharmacological Action] OR "hormonal contraceptive"[tw] OR "hormonal contraceptives"[tw] OR "Hormone Replacement Therapy"[mesh] OR "hormone replacement therapy"[tw] OR "Estrogen Replacement Therapy"[tw] OR "Oestrogen Replacement Therapy"[tw] OR "hormone replacement"[tw] OR "Estrogen Replacement"[tw] OR "Oestrogen Replacement"[tw] OR "hormone therapy"[tw] OR "hormone therapeutic"[tw] OR "hormone treatment"[tw] OR "hormone substitution"[tw] OR "Ethnicity"[mesh] OR "ethnicity"[tw] OR "ethnic"[tw] OR "Hypercholesterolemia"[mesh] OR "Hypercholesterolemia"[tw] OR "Hypercholesterolem*"[tw] OR "hypercholesterolaemia"[tw] OR "hypercholesterolaem*"[tw] OR "High Cholesterol"[tw] OR "Elevated Cholesterol"[tw] OR "Exercise"[mesh] OR "rigorous physical activity"[tw] OR "physical activity"[tw] OR "Exercise Training"[tw] OR "Physical Activities"[tw] OR "Physical Exercise"[tw] OR "Physical Exercises"[tw] OR "lean BMI"[tw] OR ("Body Mass Index"[mesh] AND "lean"[tw]) OR "lean Body Mass Index"[tw] OR "Diabetes Mellitus"[mesh] OR "Diabetes Mellitus"[tw] OR "diabetes"[tw] OR "diabetic"[tw] OR "diabetics"[tw]))) AND ("Case-Control Studies"[Mesh:noexp] OR "case control"[tw] OR "case controlled"[tw] OR "case control*"[tw] OR "Prospective Studies"[Mesh] OR "Prospective"[tw] OR "Prospectiv*"[tw] OR "Longitudinal Studies"[Mesh] OR "Longitudinal"[tw] OR "Longitudinal*"[tw] OR "Case-Control Studies"[Mesh] OR "Follow-Up Studies"[Mesh] OR "Follow-Up"[tw] OR "Retrospective Studies"[Mesh] OR "Retrospective"[tw] OR "Retrospectiv*"[tw] OR "Cohort Studies"[mesh] OR "Cohort"[tw] OR "Cohorts"[tw]) AND ("english"[la]) NOT ("Animals"[mesh] NOT "Humans"[mesh]) NOT (("Case Reports"[ptyp] OR "case report"[ti] OR "case rep"[all fields]) NOT ("Review"[ptyp] OR "review"[ti] OR "Clinical Study"[ptyp] OR "trial"[ti] OR "RCT"[ti]))) OR (((("Subarachnoid Hemorrhage"[Mesh] OR "Subarachnoid Hemorrhage"[tw] OR "Subarachnoid Hemorrhages"[tw] OR "Subarachnoid Hemorrhag*"[tw] OR "Subarachnoid Haemorrhage"[tw] OR "Subarachnoid Haemorrhages"[tw] OR "Subarachnoid Haemorrhag*"[tw] OR "Subarachnoid Bleeding"[tw] OR "Subarachnoidal Hemorrhage"[tw] OR "Subarachnoidal Hemorrhages"[tw] OR "Subarachnoidal Hemorrhag*"[tw] OR "Subarachnoidal Haemorrhage"[tw] OR "Subarachnoidal Haemorrhages"[tw] OR "Subarachnoidal Haemorrhag*"[tw] OR "Subarachnoidal Bleeding"[tw] OR "Subarachnoid Hemorrhage"[title/abstract:~3] OR "Subarachnoid Hemorrhages"[title/abstract:~3] OR "Subarachnoid Haemorrhage"[title/abstract:~3] OR "Subarachnoid Haemorrhages"[title/abstract:~3] OR "Subarachnoid Bleeding"[title/abstract:~3] OR "Subarachnoidal Hemorrhage"[title/abstract:~3] OR "Subarachnoidal Hemorrhages"[title/abstract:~3] OR "Subarachnoidal Haemorrhage"[title/abstract:~3] OR "Subarachnoidal Haemorrhages"[title/abstract:~3] OR "Subarachnoidal Bleeding"[title/abstract:~3] OR "Hemorrhagic Stroke"[Mesh] OR "hemorrhagic stroke"[tw] OR "hemorrhagic strokes"[tw] OR "haemorrhagic stroke"[tw] OR "haemorrhagic strokes"[tw] OR "hemorrhagic stroke"[title/abstract:~3] OR "hemorrhagic strokes"[title/abstract:~3] OR "haemorrhagic stroke"[title/abstract:~3] OR "haemorrhagic strokes"[title/abstract:~3]) AND ("Smoking"[majr] OR "smoking"[ti] OR "smoke"[ti] OR "smoked"[ti] OR "Nicotine"[majr] OR "Nicotine"[ti] OR "Tobacco Products"[majr] OR "Tobacco"[ti] OR "Hypertension"[majr] OR "hypertension"[ti] OR "hypertens*"[ti] OR "High Blood Pressure"[ti] OR "elevated blood pressure"[ti] OR "Alcoholism"[majr] OR "Alcoholism"[ti] OR "alcohol abuse"[ti] OR "Alcohol Dependence"[ti] OR "Alcohol Addiction"[ti] OR "Alcoholic Intoxication"[ti] OR "Ethanol Abuse"[ti] OR "Alcohol Use Disorder"[ti] OR "Alcohol Use Disorders"[ti] OR "Contraceptives, Oral"[majr] OR "oral contraception"[ti] OR "Oral Contraceptives"[ti] OR "Oral Contraceptive"[ti] OR "Contraceptive Agents, Hormonal"[majr] OR  "Contraceptive Agents, Hormonal"[Pharmacological Action] OR "hormonal contraceptive"[ti] OR "hormonal contraceptives"[ti] OR "Hormone Replacement Therapy"[majr] OR "hormone replacement therapy"[ti] OR "Estrogen Replacement Therapy"[ti] OR "Oestrogen Replacement Therapy"[ti] OR "hormone replacement"[ti] OR "Estrogen Replacement"[ti] OR "Oestrogen Replacement"[ti] OR "hormone therapy"[ti] OR "hormone therapeutic"[ti] OR "hormone treatment"[ti] OR "hormone substitution"[ti] OR "Ethnicity"[majr] OR "ethnicity"[ti] OR "ethnic"[ti] OR "Hypercholesterolemia"[majr] OR "Hypercholesterolemia"[ti] OR "Hypercholesterolem*"[ti] OR "hypercholesterolaemia"[ti] OR "hypercholesterolaem*"[ti] OR "High Cholesterol"[ti] OR "Elevated Cholesterol"[ti] OR "Exercise"[majr] OR "rigorous physical activity"[ti] OR "physical activity"[ti] OR "Exercise Training"[ti] OR "Physical Activities"[ti] OR "Physical Exercise"[ti] OR "Physical Exercises"[ti] OR "lean BMI"[ti] OR ("Body Mass Index"[majr] AND "lean"[ti]) OR "lean Body Mass Index"[ti] OR "Diabetes Mellitus"[majr] OR "Diabetes Mellitus"[ti] OR "diabetes"[ti] OR "diabetic"[ti] OR "diabetics"[ti])) OR (("Subarachnoid Hemorrhage"[majr] OR "Subarachnoid Hemorrhage"[ti] OR "Subarachnoid Hemorrhages"[ti] OR "Subarachnoid Hemorrhag*"[ti] OR "Subarachnoid Haemorrhage"[ti] OR "Subarachnoid Haemorrhages"[ti] OR "Subarachnoid Haemorrhag*"[ti] OR "Subarachnoid Bleeding"[ti] OR "Subarachnoidal Hemorrhage"[ti] OR "Subarachnoidal Hemorrhages"[ti] OR "Subarachnoidal Hemorrhag*"[ti] OR "Subarachnoidal Haemorrhage"[ti] OR "Subarachnoidal Haemorrhages"[ti] OR "Subarachnoidal Haemorrhag*"[ti] OR "Subarachnoidal Bleeding"[ti] OR "Subarachnoid Hemorrhage"[title:~3] OR "Subarachnoid Hemorrhages"[title:~3] OR "Subarachnoid Haemorrhage"[title:~3] OR "Subarachnoid Haemorrhages"[title:~3] OR "Subarachnoid Bleeding"[title:~3] OR "Subarachnoidal Hemorrhage"[title:~3] OR "Subarachnoidal Hemorrhages"[title:~3] OR "Subarachnoidal Haemorrhage"[title:~3] OR "Subarachnoidal Haemorrhages"[title:~3] OR "Subarachnoidal Bleeding"[title:~3] OR "Hemorrhagic Stroke"[majr] OR "hemorrhagic stroke"[ti] OR "hemorrhagic strokes"[ti] OR "haemorrhagic stroke"[ti] OR "haemorrhagic strokes"[ti] OR "hemorrhagic stroke"[title:~3] OR "hemorrhagic strokes"[title:~3] OR "haemorrhagic stroke"[title:~3] OR "haemorrhagic strokes"[title:~3]) AND ("Smoking"[Mesh] OR "smoking"[tw] OR "smoke"[tw] OR "smoked"[tw] OR "Nicotine"[mesh] OR "Nicotine"[tw] OR "Tobacco Products"[mesh] OR "Tobacco"[tw] OR "Hypertension"[Mesh] OR "hypertension"[tw] OR "hypertens*"[tw] OR "High Blood Pressure"[tw] OR "elevated blood pressure"[tw] OR "Alcoholism"[Mesh] OR "Alcoholism"[tw] OR "alcohol abuse"[tw] OR "Alcohol Dependence"[tw] OR "Alcohol Addiction"[tw] OR "Alcoholic Intoxication"[tw] OR "Ethanol Abuse"[tw] OR "Alcohol Use Disorder"[tw] OR "Alcohol Use Disorders"[tw] OR "Contraceptives, Oral"[Mesh] OR "oral contraception"[tw] OR "Oral Contraceptives"[tw] OR "Oral Contraceptive"[tw] OR "Contraceptive Agents, Hormonal"[mesh] OR "Contraceptive Agents, Hormonal"[Pharmacological Action] OR "hormonal contraceptive"[tw] OR "hormonal contraceptives"[tw] OR "Hormone Replacement Therapy"[Mesh] OR "hormone replacement therapy"[tw] OR "Estrogen Replacement Therapy"[tw] OR "Oestrogen Replacement Therapy"[tw] OR "hormone replacement"[tw] OR "Estrogen Replacement"[tw] OR "Oestrogen Replacement"[tw] OR "hormone therapy"[tw] OR "hormone therapeutic"[tw] OR "hormone treatment"[tw] OR "hormone substitution"[tw] OR "Ethnicity"[Mesh] OR "ethnicity"[tw] OR "ethnic"[tw] OR "Hypercholesterolemia"[Mesh] OR "Hypercholesterolemia"[tw] OR "Hypercholesterolem*"[tw] OR "hypercholesterolaemia"[tw] OR "hypercholesterolaem*"[tw] OR "High Cholesterol"[tw] OR "Elevated Cholesterol"[tw] OR "Exercise"[Mesh] OR "rigorous physical activity"[tw] OR "physical activity"[tw] OR "Exercise Training"[tw] OR "Physical Activities"[tw] OR "Physical Exercise"[tw] OR "Physical Exercises"[tw] OR "lean BMI"[tw] OR ("Body Mass Index"[Mesh] AND "lean"[tw]) OR "lean Body Mass Index"[tw] OR "Diabetes Mellitus"[Mesh] OR "Diabetes Mellitus"[tw] OR "diabetes"[tw] OR "diabetic"[tw] OR "diabetics"[tw])) OR ((("Subarachnoid Hemorrhage"[Mesh] AND "Aneurysm"[mesh]) OR "Aneurysmal subarachnoid Hemorrhage"[tw] OR "Aneurysmal subarachnoid Hemorrhages"[tw] OR "Aneurysmal subarachnoid Hemorrhag*"[tw] OR "Aneurysmal subarachnoid Haemorrhage"[tw] OR "Aneurysmal subarachnoid Haemorrhages"[tw] OR "Aneurysmal subarachnoid Haemorrhag*"[tw] OR "Aneurysmal subarachnoid Bleeding"[tw] OR "Aneurysmal subarachnoidal Hemorrhage"[tw] OR "Aneurysmal subarachnoidal Hemorrhag*"[tw] OR "Aneurysmal subarachnoid Hemorrhage"[title/abstract:~6] OR "Aneurysmal subarachnoid Hemorrhages"[title/abstract:~6] OR "Aneurysmal subarachnoid Haemorrhage"[title/abstract:~6] OR "Aneurysmal subarachnoid Haemorrhages"[title/abstract:~6] OR "Aneurysmal subarachnoid Bleeding"[title/abstract:~6] OR "Aneurysmal subarachnoidal Hemorrhage"[title/abstract:~6] OR "Aneurysmal subarachnoidal Hemorrhages"[title/abstract:~6] OR "Aneurysmal subarachnoidal Haemorrhage"[title/abstract:~6] OR "Aneurysmal subarachnoidal Haemorrhages"[title/abstract:~6] OR "Aneurysmal subarachnoidal Bleeding"[title/abstract:~6] OR "Aneurysm subarachnoid Hemorrhage"[title/abstract:~6] OR "Aneurysm subarachnoid Hemorrhages"[title/abstract:~6] OR "Aneurysm subarachnoid Haemorrhage"[title/abstract:~6] OR "Aneurysm subarachnoid Haemorrhages"[title/abstract:~6] OR "Aneurysm subarachnoid Bleeding"[title/abstract:~6] OR "Aneurysm subarachnoidal Hemorrhage"[title/abstract:~6] OR "Aneurysm subarachnoidal Hemorrhages"[title/abstract:~6] OR "Aneurysm subarachnoidal Haemorrhage"[title/abstract:~6] OR "Aneurysm subarachnoidal Haemorrhages"[title/abstract:~6] OR "Aneurysm subarachnoidal Bleeding"[title/abstract:~6] OR "Aneurysms subarachnoid Hemorrhage"[title/abstract:~6] OR "Aneurysms subarachnoid Hemorrhages"[title/abstract:~6] OR "Aneurysms subarachnoid Haemorrhage"[title/abstract:~6] OR "Aneurysms subarachnoid Haemorrhages"[title/abstract:~6] OR "Aneurysms subarachnoid Bleeding"[title/abstract:~6] OR "Aneurysms subarachnoidal Hemorrhage"[title/abstract:~6] OR "Aneurysms subarachnoidal Hemorrhages"[title/abstract:~6] OR "Aneurysms subarachnoidal Haemorrhage"[title/abstract:~6] OR "Aneurysms subarachnoidal Haemorrhages"[title/abstract:~6] OR "Aneurysms subarachnoidal Bleeding"[title/abstract:~6]) AND ("Smoking"[Mesh] OR "smoking"[tw] OR "smoke"[tw] OR "smoked"[tw] OR "Nicotine"[mesh] OR "Nicotine"[tw] OR "Tobacco Products"[mesh] OR "Tobacco"[tw] OR "Hypertension"[Mesh] OR "hypertension"[tw] OR "hypertens*"[tw] OR "High Blood Pressure"[tw] OR "elevated blood pressure"[tw] OR "Alcoholism"[Mesh] OR "Alcoholism"[tw] OR "alcohol abuse"[tw] OR "Alcohol Dependence"[tw] OR "Alcohol Addiction"[tw] OR "Alcoholic Intoxication"[tw] OR "Ethanol Abuse"[tw] OR "Alcohol Use Disorder"[tw] OR "Alcohol Use Disorders"[tw] OR "Contraceptives, Oral"[Mesh] OR "oral contraception"[tw] OR "Oral Contraceptives"[tw] OR "Oral Contraceptive"[tw] OR "Contraceptive Agents, Hormonal"[mesh] OR "Contraceptive Agents, Hormonal"[Pharmacological Action] OR "hormonal contraceptive"[tw] OR "hormonal contraceptives"[tw] OR "Hormone Replacement Therapy"[Mesh] OR "hormone replacement therapy"[tw] OR "Estrogen Replacement Therapy"[tw] OR "Oestrogen Replacement Therapy"[tw] OR "hormone replacement"[tw] OR "Estrogen Replacement"[tw] OR "Oestrogen Replacement"[tw] OR "hormone therapy"[tw] OR "hormone therapeutic"[tw] OR "hormone treatment"[tw] OR "hormone substitution"[tw] OR "Ethnicity"[Mesh] OR "ethnicity"[tw] OR "ethnic"[tw] OR "Hypercholesterolemia"[Mesh] OR "Hypercholesterolemia"[tw] OR "Hypercholesterolem*"[tw] OR "hypercholesterolaemia"[tw] OR "hypercholesterolaem*"[tw] OR "High Cholesterol"[tw] OR "Elevated Cholesterol"[tw] OR "Exercise"[Mesh] OR "rigorous physical activity"[tw] OR "physical activity"[tw] OR "Exercise Training"[tw] OR "Physical Activities"[tw] OR "Physical Exercise"[tw] OR "Physical Exercises"[tw] OR "lean BMI"[tw] OR ("Body Mass Index"[Mesh] AND "lean"[tw]) OR "lean Body Mass Index"[tw] OR "Diabetes Mellitus"[Mesh] OR "Diabetes Mellitus"[tw] OR "diabetes"[tw] OR "diabetic"[tw] OR "diabetics"[tw]))) AND ("Case-Control Studies"[Mesh:noexp] OR "case control"[tw] OR "case controlled"[tw] OR "case control*"[tw] OR "Prospective Studies"[Mesh] OR "Prospective"[tw] OR "Prospectiv*"[tw] OR "Longitudinal Studies"[Mesh] OR "Longitudinal"[tw] OR "Longitudinal*"[tw] OR "Case-Control Studies"[Mesh] OR "Follow-Up Studies"[Mesh] OR "Follow-Up"[tw] OR "Retrospective Studies"[Mesh] OR "Retrospective"[tw] OR "Retrospectiv*"[tw] OR "Cohort Studies"[mesh] OR "Cohort"[tw] OR "Cohorts"[tw]) AND ("english"[la]) NOT ("Animals"[mesh] NOT "Humans"[mesh]) NOT (("Case Reports"[ptyp] OR "case report"[ti] OR "case rep"[all fields]) NOT ("Review"[ptyp] OR "review"[ti] OR "Clinical Study"[ptyp] OR "trial"[ti] OR "RCT"[ti])))

**II. Embase (OVID version)**

*(Final search run on 01-03-2024; 582 unique items)*

(((((exp *"Intracranial Aneurysm"/ OR "Intracranial Aneurysm".ti,ab OR "Intracranial Aneurysms".ti,ab OR "cerebral aneurysm".ti,ab OR "cerebral aneurysms".ti,ab OR "Brain Aneurysm".ti,ab OR "Brain Aneurysms".ti,ab OR "Berry Aneurysm".ti,ab OR "Berry Aneurysms".ti,ab OR "Saccular Aneurysm".ti,ab OR "Saccular Aneurysms".ti,ab OR (("Intracranial" ADJ3 "Aneurysm") OR ("Intracranial" ADJ3 "Aneurysms") OR ("cerebral" ADJ3 "aneurysm") OR ("cerebral" ADJ3 "aneurysms") OR ("brain" ADJ3 "aneurysm") OR ("brain" ADJ3 "aneurysms") OR ("berry" ADJ3 "aneurysm") OR ("berry" ADJ3 "aneurysms") OR ("saccular" ADJ3 "aneurysm") OR ("saccular" ADJ3 "aneurysms")).ti,ab) AND (exp *"Smoking"/ OR "smoking".ti OR "smoke".ti OR "smoked".ti OR exp *"Nicotine"/ OR "Nicotine".ti OR exp *"Tobacco"/ OR "Tobacco".ti OR exp *"Hypertension"/ OR "hypertension".ti OR "hypertens*".ti OR "High Blood Pressure".ti OR "elevated blood pressure".ti OR exp *"Alcoholism"/ OR exp *"Alcohol Abuse"/ OR "Alcoholism".ti OR "alcohol abuse".ti OR "Alcohol Dependence".ti OR "Alcohol Addiction".ti OR "Alcoholic Intoxication".ti OR "Ethanol Abuse".ti OR "Alcohol Use Disorder".ti OR "Alcohol Use Disorders".ti OR exp *"oral contraceptive agent"/ OR exp *"oral contraception"/ OR "oral contraception".ti OR "Oral Contraceptives".ti OR "Oral Contraceptive".ti OR "hormonal contraceptive agent"/ OR "hormonal contraceptive".ti OR "hormonal contraceptives".ti OR exp *"Hormone Substitution"/ OR "hormone replacement therapy".ti OR "Estrogen Replacement Therapy".ti OR "Oestrogen Replacement Therapy".ti OR "hormone replacement".ti OR "Estrogen Replacement".ti OR "Oestrogen Replacement".ti OR "hormone therapy".ti OR "hormone therapeutic".ti OR "hormone treatment".ti OR "hormone substitution".ti OR exp "hormonal therapy"/ OR exp *"Ethnicity"/ OR "ethnicity".ti OR "ethnic".ti OR exp *"Hypercholesterolemia"/ OR "Hypercholesterolemia".ti OR "Hypercholesterolem*".ti OR "hypercholesterolaemia".ti OR "hypercholesterolaem*".ti OR "High Cholesterol".ti OR "Elevated Cholesterol".ti OR exp *"Exercise"/ OR exp *"physical activity"/ OR "rigorous physical activity".ti OR "physical activity".ti OR "Exercise Training".ti OR "Physical Activities".ti OR "Physical Exercise".ti OR "Physical Exercises".ti OR "lean BMI".ti OR (exp *"Body Mass"/ AND "lean".ti) OR "lean Body Mass Index".ti OR exp *"Diabetes Mellitus"/ OR "Diabetes Mellitus".ti OR "diabetes".ti OR "diabetic".ti OR "diabetics".ti)) OR ((exp *"Intracranial Aneurysm"/ OR "Intracranial Aneurysm".ti OR "Intracranial Aneurysms".ti OR "cerebral aneurysm".ti OR "cerebral aneurysms".ti OR "Brain Aneurysm".ti OR "Brain Aneurysms".ti OR "Berry Aneurysm".ti OR "Berry Aneurysms".ti OR "Saccular Aneurysm".ti OR "Saccular Aneurysms".ti OR (("Intracranial" ADJ3 "Aneurysm") OR ("Intracranial" ADJ3 "Aneurysms") OR ("cerebral" ADJ3 "aneurysm") OR ("cerebral" ADJ3 "aneurysms") OR ("brain" ADJ3 "aneurysm") OR ("brain" ADJ3 "aneurysms") OR ("berry" ADJ3 "aneurysm") OR ("berry" ADJ3 "aneurysms") OR ("saccular" ADJ3 "aneurysm") OR ("saccular" ADJ3 "aneurysms")).ti) AND (exp *"Smoking"/ OR "smoking".ti,ab OR "smoke".ti,ab OR "smoked".ti,ab OR exp *"Nicotine"/ OR "Nicotine".ti,ab OR exp *"Tobacco"/ OR "Tobacco".ti,ab OR exp *"Hypertension"/ OR "hypertension".ti,ab OR "hypertens*".ti,ab OR "High Blood Pressure".ti,ab OR "elevated blood pressure".ti,ab OR exp *"Alcoholism"/ OR exp *"Alcohol Abuse"/ OR "Alcoholism".ti,ab OR "alcohol abuse".ti,ab OR "Alcohol Dependence".ti,ab OR "Alcohol Addiction".ti,ab OR "Alcoholic Intoxication".ti,ab OR "Ethanol Abuse".ti,ab OR "Alcohol Use Disorder".ti,ab OR "Alcohol Use Disorders".ti,ab OR exp *"oral contraceptive agent"/ OR exp *"oral contraception"/ OR "oral contraception".ti,ab OR "Oral Contraceptives".ti,ab OR "Oral Contraceptive".ti,ab OR "hormonal contraceptive agent"/ OR "hormonal contraceptive".ti,ab OR "hormonal contraceptives".ti,ab OR exp *"Hormone Substitution"/ OR "hormone replacement therapy".ti,ab OR "Estrogen Replacement Therapy".ti,ab OR "Oestrogen Replacement Therapy".ti,ab OR "hormone replacement".ti,ab OR "Estrogen Replacement".ti,ab OR "Oestrogen Replacement".ti,ab OR "hormone therapy".ti,ab OR "hormone therapeutic".ti,ab OR "hormone treatment".ti,ab OR "hormone substitution".ti,ab OR exp "hormonal therapy"/ OR exp *"Ethnicity"/ OR "ethnicity".ti,ab OR "ethnic".ti,ab OR exp *"Hypercholesterolemia"/ OR "Hypercholesterolemia".ti,ab OR "Hypercholesterolem*".ti,ab OR "hypercholesterolaemia".ti,ab OR "hypercholesterolaem*".ti,ab OR "High Cholesterol".ti,ab OR "Elevated Cholesterol".ti,ab OR exp *"Exercise"/ OR exp *"physical activity"/ OR "rigorous physical activity".ti,ab OR "physical activity".ti,ab OR "Exercise Training".ti,ab OR "Physical Activities".ti,ab OR "Physical Exercise".ti,ab OR "Physical Exercises".ti,ab OR "lean BMI".ti,ab OR (exp *"Body Mass"/ AND "lean".ti,ab) OR "lean Body Mass Index".ti,ab OR exp *"Diabetes Mellitus"/ OR "Diabetes Mellitus".ti,ab OR "diabetes".ti,ab OR "diabetic".ti,ab OR "diabetics".ti,ab)) OR ((exp *"Intracranial Aneurysm"/ OR "Intracranial Aneurysm".ti,ab OR "Intracranial Aneurysms".ti,ab OR "cerebral aneurysm".ti,ab OR "cerebral aneurysms".ti,ab OR "Brain Aneurysm".ti,ab OR "Brain Aneurysms".ti,ab OR "Berry Aneurysm".ti,ab OR "Berry Aneurysms".ti,ab OR "Saccular Aneurysm".ti,ab OR "Saccular Aneurysms".ti,ab OR (("Intracranial" ADJ3 "Aneurysm") OR ("Intracranial" ADJ3 "Aneurysms") OR ("cerebral" ADJ3 "aneurysm") OR ("cerebral" ADJ3 "aneurysms") OR ("brain" ADJ3 "aneurysm") OR ("brain" ADJ3 "aneurysms") OR ("berry" ADJ3 "aneurysm") OR ("berry" ADJ3 "aneurysms") OR ("saccular" ADJ3 "aneurysm") OR ("saccular" ADJ3 "aneurysms")).ti,ab) AND ("unruptured".ti,ab OR "un ruptured".ti,ab OR "unruptur*".ti,ab OR "un ruptur*".ti,ab OR "incidental".ti,ab OR "incidental*".ti,ab) AND (exp *"Smoking"/ OR "smoking".ti,ab OR "smoke".ti,ab OR "smoked".ti,ab OR exp *"Nicotine"/ OR "Nicotine".ti,ab OR exp *"Tobacco"/ OR "Tobacco".ti,ab OR exp *"Hypertension"/ OR "hypertension".ti,ab OR "hypertens*".ti,ab OR "High Blood Pressure".ti,ab OR "elevated blood pressure".ti,ab OR exp *"Alcoholism"/ OR exp *"Alcohol Abuse"/ OR "Alcoholism".ti,ab OR "alcohol abuse".ti,ab OR "Alcohol Dependence".ti,ab OR "Alcohol Addiction".ti,ab OR "Alcoholic Intoxication".ti,ab OR "Ethanol Abuse".ti,ab OR "Alcohol Use Disorder".ti,ab OR "Alcohol Use Disorders".ti,ab OR exp *"oral contraceptive agent"/ OR exp *"oral contraception"/ OR "oral contraception".ti,ab OR "Oral Contraceptives".ti,ab OR "Oral Contraceptive".ti,ab OR "hormonal contraceptive agent"/ OR "hormonal contraceptive".ti,ab OR "hormonal contraceptives".ti,ab OR exp *"Hormone Substitution"/ OR "hormone replacement therapy".ti,ab OR "Estrogen Replacement Therapy".ti,ab OR "Oestrogen Replacement Therapy".ti,ab OR "hormone replacement".ti,ab OR "Estrogen Replacement".ti,ab OR "Oestrogen Replacement".ti,ab OR "hormone therapy".ti,ab OR "hormone therapeutic".ti,ab OR "hormone treatment".ti,ab OR "hormone substitution".ti,ab OR exp "hormonal therapy"/ OR exp *"Ethnicity"/ OR "ethnicity".ti,ab OR "ethnic".ti,ab OR exp *"Hypercholesterolemia"/ OR "Hypercholesterolemia".ti,ab OR "Hypercholesterolem*".ti,ab OR "hypercholesterolaemia".ti,ab OR "hypercholesterolaem*".ti,ab OR "High Cholesterol".ti,ab OR "Elevated Cholesterol".ti,ab OR exp *"Exercise"/ OR exp *"physical activity"/ OR "rigorous physical activity".ti,ab OR "physical activity".ti,ab OR "Exercise Training".ti,ab OR "Physical Activities".ti,ab OR "Physical Exercise".ti,ab OR "Physical Exercises".ti,ab OR "lean BMI".ti,ab OR (exp *"Body Mass"/ AND "lean".ti,ab) OR "lean Body Mass Index".ti,ab OR exp *"Diabetes Mellitus"/ OR "Diabetes Mellitus".ti,ab OR "diabetes".ti,ab OR "diabetic".ti,ab OR "diabetics".ti,ab))) AND (exp "Case Control Study"/ OR "case control".mp OR "case controlled".mp OR "case control*".mp OR exp "Prospective Study"/ OR "Prospective".mp OR "Prospectiv*".mp OR exp "Longitudinal Study"/ OR "Longitudinal".mp OR "Longitudinal*".mp OR exp *"Follow Up"/ OR "Follow-Up".mp OR exp "Retrospective Study"/ OR "Retrospective".mp OR "Retrospectiv*".mp OR exp "Cohort Analysis"/ OR "Cohort".mp OR "Cohorts".mp) AND ("english".la) NOT (exp "Animals"/ NOT exp "Humans"/) NOT (("Case Report"/ OR "case report".ti OR (case AND (report OR reports)).jw) NOT (exp "Review"/ OR "review".ti OR "Clinical Study"/ OR exp "Clinical Trial"/ OR "trial".ti OR "RCT".ti)) OR ((((exp *"Subarachnoid Hemorrhage"/ OR "Subarachnoid Hemorrhage".ti,ab OR "Subarachnoid Hemorrhages".ti,ab OR "Subarachnoid Hemorrhag*".ti,ab OR "Subarachnoid Haemorrhage".ti,ab OR "Subarachnoid Haemorrhages".ti,ab OR "Subarachnoid Haemorrhag*".ti,ab OR "Subarachnoid Bleeding".ti,ab OR "Subarachnoidal Hemorrhage".ti,ab OR "Subarachnoidal Hemorrhages".ti,ab OR "Subarachnoidal Hemorrhag*".ti,ab OR "Subarachnoidal Haemorrhage".ti,ab OR "Subarachnoidal Haemorrhages".ti,ab OR "Subarachnoidal Haemorrhag*".ti,ab OR "Subarachnoidal Bleeding".ti,ab OR (("Subarachnoid" ADJ3 "Hemorrhage") OR ("Subarachnoid" ADJ3 "Hemorrhages") OR ("Subarachnoid" ADJ3 "Haemorrhage") OR ("Subarachnoid" ADJ3 "Haemorrhages") OR ("Subarachnoid" ADJ3 "Bleeding") OR ("Subarachnoidal" ADJ3 "Hemorrhage") OR ("Subarachnoidal" ADJ3 "Hemorrhages") OR ("Subarachnoidal" ADJ3 "Haemorrhage") OR ("Subarachnoidal" ADJ3 "Haemorrhages") OR ("Subarachnoidal" ADJ3 "Bleeding")).ti,ab OR "hemorrhagic stroke".ti,ab OR "hemorrhagic strokes".ti,ab OR "haemorrhagic stroke".ti,ab OR "haemorrhagic strokes".ti,ab OR (("hemorrhagic" ADJ3 "stroke") OR ("hemorrhagic" ADJ3 "strokes") OR ("haemorrhagic" ADJ3 "stroke") OR ("haemorrhagic" ADJ3 "strokes")).ti,ab) AND (exp *"Smoking"/ OR "smoking".ti OR "smoke".ti OR "smoked".ti OR exp *"Nicotine"/ OR "Nicotine".ti OR exp *"Tobacco"/ OR "Tobacco".ti OR exp *"Hypertension"/ OR "hypertension".ti OR "hypertens*".ti OR "High Blood Pressure".ti OR "elevated blood pressure".ti OR exp *"Alcoholism"/ OR exp *"Alcohol Abuse"/ OR "Alcoholism".ti OR "alcohol abuse".ti OR "Alcohol Dependence".ti OR "Alcohol Addiction".ti OR "Alcoholic Intoxication".ti OR "Ethanol Abuse".ti OR "Alcohol Use Disorder".ti OR "Alcohol Use Disorders".ti OR exp *"oral contraceptive agent"/ OR exp *"oral contraception"/ OR "oral contraception".ti OR "Oral Contraceptives".ti OR "Oral Contraceptive".ti OR "hormonal contraceptive agent"/ OR "hormonal contraceptive".ti OR "hormonal contraceptives".ti OR exp *"Hormone Substitution"/ OR "hormone replacement therapy".ti OR "Estrogen Replacement Therapy".ti OR "Oestrogen Replacement Therapy".ti OR "hormone replacement".ti OR "Estrogen Replacement".ti OR "Oestrogen Replacement".ti OR "hormone therapy".ti OR "hormone therapeutic".ti OR "hormone treatment".ti OR "hormone substitution".ti OR exp "hormonal therapy"/ OR exp *"Ethnicity"/ OR "ethnicity".ti OR "ethnic".ti OR exp *"Hypercholesterolemia"/ OR "Hypercholesterolemia".ti OR "Hypercholesterolem*".ti OR "hypercholesterolaemia".ti OR "hypercholesterolaem*".ti OR "High Cholesterol".ti OR "Elevated Cholesterol".ti OR exp *"Exercise"/ OR exp *"physical activity"/ OR "rigorous physical activity".ti OR "physical activity".ti OR "Exercise Training".ti OR "Physical Activities".ti OR "Physical Exercise".ti OR "Physical Exercises".ti OR "lean BMI".ti OR (exp *"Body Mass"/ AND "lean".ti) OR "lean Body Mass Index".ti OR exp *"Diabetes Mellitus"/ OR "Diabetes Mellitus".ti OR "diabetes".ti OR "diabetic".ti OR "diabetics".ti)) OR ((exp *"Subarachnoid Hemorrhage"/ OR "Subarachnoid Hemorrhage".ti OR "Subarachnoid Hemorrhages".ti OR "Subarachnoid Hemorrhag*".ti OR "Subarachnoid Haemorrhage".ti OR "Subarachnoid Haemorrhages".ti OR "Subarachnoid Haemorrhag*".ti OR "Subarachnoid Bleeding".ti OR "Subarachnoidal Hemorrhage".ti OR "Subarachnoidal Hemorrhages".ti OR "Subarachnoidal Hemorrhag*".ti OR "Subarachnoidal Haemorrhage".ti OR "Subarachnoidal Haemorrhages".ti OR "Subarachnoidal Haemorrhag*".ti OR "Subarachnoidal Bleeding".ti OR (("Subarachnoid" ADJ3 "Hemorrhage") OR ("Subarachnoid" ADJ3 "Hemorrhages") OR ("Subarachnoid" ADJ3 "Haemorrhage") OR ("Subarachnoid" ADJ3 "Haemorrhages") OR ("Subarachnoid" ADJ3 "Bleeding") OR ("Subarachnoidal" ADJ3 "Hemorrhage") OR ("Subarachnoidal" ADJ3 "Hemorrhages") OR ("Subarachnoidal" ADJ3 "Haemorrhage") OR ("Subarachnoidal" ADJ3 "Haemorrhages") OR ("Subarachnoidal" ADJ3 "Bleeding")).ti OR "hemorrhagic stroke".ti OR "hemorrhagic strokes".ti OR "haemorrhagic stroke".ti OR "haemorrhagic strokes".ti OR (("hemorrhagic" ADJ3 "stroke") OR ("hemorrhagic" ADJ3 "strokes") OR ("haemorrhagic" ADJ3 "stroke") OR ("haemorrhagic" ADJ3 "strokes")).ti) AND (exp *"Smoking"/ OR "smoking".ti,ab OR "smoke".ti,ab OR "smoked".ti,ab OR exp *"Nicotine"/ OR "Nicotine".ti,ab OR exp *"Tobacco"/ OR "Tobacco".ti,ab OR exp *"Hypertension"/ OR "hypertension".ti,ab OR "hypertens*".ti,ab OR "High Blood Pressure".ti,ab OR "elevated blood pressure".ti,ab OR exp *"Alcoholism"/ OR exp *"Alcohol Abuse"/ OR "Alcoholism".ti,ab OR "alcohol abuse".ti,ab OR "Alcohol Dependence".ti,ab OR "Alcohol Addiction".ti,ab OR "Alcoholic Intoxication".ti,ab OR "Ethanol Abuse".ti,ab OR "Alcohol Use Disorder".ti,ab OR "Alcohol Use Disorders".ti,ab OR exp *"oral contraceptive agent"/ OR exp *"oral contraception"/ OR "oral contraception".ti,ab OR "Oral Contraceptives".ti,ab OR "Oral Contraceptive".ti,ab OR "hormonal contraceptive agent"/ OR "hormonal contraceptive".ti,ab OR "hormonal contraceptives".ti,ab OR exp *"Hormone Substitution"/ OR "hormone replacement therapy".ti,ab OR "Estrogen Replacement Therapy".ti,ab OR "Oestrogen Replacement Therapy".ti,ab OR "hormone replacement".ti,ab OR "Estrogen Replacement".ti,ab OR "Oestrogen Replacement".ti,ab OR "hormone therapy".ti,ab OR "hormone therapeutic".ti,ab OR "hormone treatment".ti,ab OR "hormone substitution".ti,ab OR exp "hormonal therapy"/ OR exp *"Ethnicity"/ OR "ethnicity".ti,ab OR "ethnic".ti,ab OR exp *"Hypercholesterolemia"/ OR "Hypercholesterolemia".ti,ab OR "Hypercholesterolem*".ti,ab OR "hypercholesterolaemia".ti,ab OR "hypercholesterolaem*".ti,ab OR "High Cholesterol".ti,ab OR "Elevated Cholesterol".ti,ab OR exp *"Exercise"/ OR exp *"physical activity"/ OR "rigorous physical activity".ti,ab OR "physical activity".ti,ab OR "Exercise Training".ti,ab OR "Physical Activities".ti,ab OR "Physical Exercise".ti,ab OR "Physical Exercises".ti,ab OR "lean BMI".ti,ab OR (exp *"Body Mass"/ AND "lean".ti,ab) OR "lean Body Mass Index".ti,ab OR exp *"Diabetes Mellitus"/ OR "Diabetes Mellitus".ti,ab OR "diabetes".ti,ab OR "diabetic".ti,ab OR "diabetics".ti,ab)) OR (((exp *"Subarachnoid Hemorrhage"/ AND exp *"Aneurysm"/) OR "Subarachnoid Hemorrhage".ti,ab OR "aneurysmal Subarachnoid Hemorrhages".ti,ab OR "aneurysmal Subarachnoid Hemorrhag*".ti,ab OR "aneurysmal Subarachnoid Haemorrhage".ti,ab OR "aneurysmal Subarachnoid Haemorrhages".ti,ab OR "aneurysmal Subarachnoid Haemorrhag*".ti,ab OR "aneurysmal Subarachnoid Bleeding".ti,ab OR "aneurysmal Subarachnoidal Hemorrhage".ti,ab OR "aneurysmal Subarachnoidal Hemorrhages".ti,ab OR "aneurysmal Subarachnoidal Hemorrhag*".ti,ab OR "aneurysmal Subarachnoidal Haemorrhage".ti,ab OR "aneurysmal Subarachnoidal Haemorrhages".ti,ab OR "aneurysmal Subarachnoidal Haemorrhag*".ti,ab OR "aneurysmal Subarachnoidal Bleeding".ti,ab OR (( "aneurysm*" ADJ3 "subarachnoid" ADJ3 "Hemorrhage") OR ( "aneurysm*" ADJ3 "subarachnoid" ADJ3 "Hemorrhages") OR ( "aneurysm*" ADJ3 "subarachnoid" ADJ3 "Haemorrhage") OR ( "aneurysm*" ADJ3 "subarachnoid" ADJ3 "Haemorrhages") OR ( "aneurysm*" ADJ3 "subarachnoid" ADJ3 "Bleeding") OR ("Subarachnoidal" ADJ3 "Hemorrhage") OR ("Subarachnoidal" ADJ3 "Hemorrhages") OR ("Subarachnoidal" ADJ3 "Haemorrhage") OR ("Subarachnoidal" ADJ3 "Haemorrhages") OR ("Subarachnoidal" ADJ3 "Bleeding")).ti,ab) AND (exp *"Smoking"/ OR "smoking".ti,ab OR "smoke".ti,ab OR "smoked".ti,ab OR exp *"Nicotine"/ OR "Nicotine".ti,ab OR exp *"Tobacco"/ OR "Tobacco".ti,ab OR exp *"Hypertension"/ OR "hypertension".ti,ab OR "hypertens*".ti,ab OR "High Blood Pressure".ti,ab OR "elevated blood pressure".ti,ab OR exp *"Alcoholism"/ OR exp *"Alcohol Abuse"/ OR "Alcoholism".ti,ab OR "alcohol abuse".ti,ab OR "Alcohol Dependence".ti,ab OR "Alcohol Addiction".ti,ab OR "Alcoholic Intoxication".ti,ab OR "Ethanol Abuse".ti,ab OR "Alcohol Use Disorder".ti,ab OR "Alcohol Use Disorders".ti,ab OR exp *"oral contraceptive agent"/ OR exp *"oral contraception"/ OR "oral contraception".ti,ab OR "Oral Contraceptives".ti,ab OR "Oral Contraceptive".ti,ab OR "hormonal contraceptive agent"/ OR "hormonal contraceptive".ti,ab OR "hormonal contraceptives".ti,ab OR exp *"Hormone Substitution"/ OR "hormone replacement therapy".ti,ab OR "Estrogen Replacement Therapy".ti,ab OR "Oestrogen Replacement Therapy".ti,ab OR "hormone replacement".ti,ab OR "Estrogen Replacement".ti,ab OR "Oestrogen Replacement".ti,ab OR "hormone therapy".ti,ab OR "hormone therapeutic".ti,ab OR "hormone treatment".ti,ab OR "hormone substitution".ti,ab OR exp "hormonal therapy"/ OR exp *"Ethnicity"/ OR "ethnicity".ti,ab OR "ethnic".ti,ab OR exp *"Hypercholesterolemia"/ OR "Hypercholesterolemia".ti,ab OR "Hypercholesterolem*".ti,ab OR "hypercholesterolaemia".ti,ab OR "hypercholesterolaem*".ti,ab OR "High Cholesterol".ti,ab OR "Elevated Cholesterol".ti,ab OR exp *"Exercise"/ OR exp *"physical activity"/ OR "rigorous physical activity".ti,ab OR "physical activity".ti,ab OR "Exercise Training".ti,ab OR "Physical Activities".ti,ab OR "Physical Exercise".ti,ab OR "Physical Exercises".ti,ab OR "lean BMI".ti,ab OR (exp *"Body Mass"/ AND "lean".ti,ab) OR "lean Body Mass Index".ti,ab OR exp *"Diabetes Mellitus"/ OR "Diabetes Mellitus".ti,ab OR "diabetes".ti,ab OR "diabetic".ti,ab OR "diabetics".ti,ab))) AND (exp "Case Control Study"/ OR "case control".mp OR "case controlled".mp OR "case control*".mp OR exp "Prospective Study"/ OR "Prospective".mp OR "Prospectiv*".mp OR exp "Longitudinal Study"/ OR "Longitudinal".mp OR "Longitudinal*".mp OR exp *"Follow Up"/ OR "Follow-Up".mp OR exp "Retrospective Study"/ OR "Retrospective".mp OR "Retrospectiv*".mp OR exp "Cohort Analysis"/ OR "Cohort".mp OR "Cohorts".mp) AND ("english".la) NOT (exp "Animals"/ NOT exp "Humans"/) NOT (("Case Report"/ OR "case report".ti OR (case AND (report OR reports)).jw) NOT (exp "Review"/ OR "review".ti OR "Clinical Study"/ OR exp "Clinical Trial"/ OR "trial".ti OR "RCT".ti))))) NOT (conference review or conference abstract).pt

**III. Web of Science**

*(Final search run on 01-03-2024; 146 unique items)*

((((TI=("Intracranial Aneurysm" OR "Intracranial Aneurysm" OR "Intracranial Aneurysms" OR "cerebral aneurysm" OR "cerebral aneurysms" OR "Brain Aneurysm" OR "Brain Aneurysms" OR "Berry Aneurysm" OR "Berry Aneurysms" OR "Saccular Aneurysm" OR "Saccular Aneurysms" OR (("Intracranial" NEAR/3 "Aneurysm") OR ("Intracranial" NEAR/3 "Aneurysms") OR ("cerebral" NEAR/3 "aneurysm") OR ("cerebral" NEAR/3 "aneurysms") OR ("brain" NEAR/3 "aneurysm") OR ("brain" NEAR/3 "aneurysms") OR ("berry" NEAR/3 "aneurysm") OR ("berry" NEAR/3 "aneurysms") OR ("saccular" NEAR/3 "aneurysm") OR ("saccular" NEAR/3 "aneurysms"))) OR AK=("Intracranial Aneurysm" OR "Intracranial Aneurysm" OR "Intracranial Aneurysms" OR "cerebral aneurysm" OR "cerebral aneurysms" OR "Brain Aneurysm" OR "Brain Aneurysms" OR "Berry Aneurysm" OR "Berry Aneurysms" OR "Saccular Aneurysm" OR "Saccular Aneurysms" OR (("Intracranial" NEAR/3 "Aneurysm") OR ("Intracranial" NEAR/3 "Aneurysms") OR ("cerebral" NEAR/3 "aneurysm") OR ("cerebral" NEAR/3 "aneurysms") OR ("brain" NEAR/3 "aneurysm") OR ("brain" NEAR/3 "aneurysms") OR ("berry" NEAR/3 "aneurysm") OR ("berry" NEAR/3 "aneurysms") OR ("saccular" NEAR/3 "aneurysm") OR ("saccular" NEAR/3 "aneurysms"))) OR AB=("Intracranial Aneurysm" OR "Intracranial Aneurysm" OR "Intracranial Aneurysms" OR "cerebral aneurysm" OR "cerebral aneurysms" OR "Brain Aneurysm" OR "Brain Aneurysms" OR "Berry Aneurysm" OR "Berry Aneurysms" OR "Saccular Aneurysm" OR "Saccular Aneurysms" OR (("Intracranial" NEAR/3 "Aneurysm") OR ("Intracranial" NEAR/3 "Aneurysms") OR ("cerebral" NEAR/3 "aneurysm") OR ("cerebral" NEAR/3 "aneurysms") OR ("brain" NEAR/3 "aneurysm") OR ("brain" NEAR/3 "aneurysms") OR ("berry" NEAR/3 "aneurysm") OR ("berry" NEAR/3 "aneurysms") OR ("saccular" NEAR/3 "aneurysm") OR ("saccular" NEAR/3 "aneurysms")))) AND TI=("Smoking" OR "smoking" OR "smoke" OR "smoked" OR "Nicotine" OR "Nicotine" OR "Tobacco" OR "Tobacco" OR "Hypertension" OR "hypertension" OR "hypertens*" OR "High Blood Pressure" OR "elevated blood pressure" OR "Alcoholism" OR "Alcohol Abuse" OR "Alcoholism" OR "alcohol abuse" OR "Alcohol Dependence" OR "Alcohol Addiction" OR "Alcoholic Intoxication" OR "Ethanol Abuse" OR "Alcohol Use Disorder" OR "Alcohol Use Disorders" OR "oral contraceptive agent" OR "oral contraception" OR "oral contraception" OR "Oral Contraceptives" OR "Oral Contraceptive" OR "hormonal contraceptive agent" OR "hormonal contraceptive" OR "hormonal contraceptives" OR "Hormone Substitution" OR "hormone replacement therapy" OR "Estrogen Replacement Therapy" OR "Oestrogen Replacement Therapy" OR "hormone replacement" OR "Estrogen Replacement" OR "Oestrogen Replacement" OR "hormone therapy" OR "hormone therapeutic" OR "hormone treatment" OR "hormone substitution" OR exp "hormonal therapy" OR "Ethnicity" OR "ethnicity" OR "ethnic" OR "Hypercholesterolemia" OR "Hypercholesterolemia" OR "Hypercholesterolem*" OR "hypercholesterolaemia" OR "hypercholesterolaem*" OR "High Cholesterol" OR "Elevated Cholesterol" OR "Exercise" OR "physical activity" OR "rigorous physical activity" OR "physical activity" OR "Exercise Training" OR "Physical Activities" OR "Physical Exercise" OR "Physical Exercises" OR "lean BMI" OR ("Body Mass" AND "lean") OR "lean Body Mass Index" OR "Diabetes Mellitus" OR "Diabetes Mellitus" OR "diabetes" OR "diabetic" OR "diabetics")) OR (TI=("Intracranial Aneurysm" OR "Intracranial Aneurysm" OR "Intracranial Aneurysms" OR "cerebral aneurysm" OR "cerebral aneurysms" OR "Brain Aneurysm" OR "Brain Aneurysms" OR "Berry Aneurysm" OR "Berry Aneurysms" OR "Saccular Aneurysm" OR "Saccular Aneurysms" OR (("Intracranial" NEAR/3 "Aneurysm") OR ("Intracranial" NEAR/3 "Aneurysms") OR ("cerebral" NEAR/3 "aneurysm") OR ("cerebral" NEAR/3 "aneurysms") OR ("brain" NEAR/3 "aneurysm") OR ("brain" NEAR/3 "aneurysms") OR ("berry" NEAR/3 "aneurysm") OR ("berry" NEAR/3 "aneurysms") OR ("saccular" NEAR/3 "aneurysm") OR ("saccular" NEAR/3 "aneurysms"))) AND (TI=("Smoking" OR "smoking" OR "smoke" OR "smoked" OR "Nicotine" OR "Nicotine" OR "Tobacco" OR "Tobacco" OR "Hypertension" OR "hypertension" OR "hypertens*" OR "High Blood Pressure" OR "elevated blood pressure" OR "Alcoholism" OR "Alcohol Abuse" OR "Alcoholism" OR "alcohol abuse" OR "Alcohol Dependence" OR "Alcohol Addiction" OR "Alcoholic Intoxication" OR "Ethanol Abuse" OR "Alcohol Use Disorder" OR "Alcohol Use Disorders" OR "oral contraceptive agent" OR "oral contraception" OR "oral contraception" OR "Oral Contraceptives" OR "Oral Contraceptive" OR "hormonal contraceptive agent" OR "hormonal contraceptive" OR "hormonal contraceptives" OR "Hormone Substitution" OR "hormone replacement therapy" OR "Estrogen Replacement Therapy" OR "Oestrogen Replacement Therapy" OR "hormone replacement" OR "Estrogen Replacement" OR "Oestrogen Replacement" OR "hormone therapy" OR "hormone therapeutic" OR "hormone treatment" OR "hormone substitution" OR exp "hormonal therapy" OR "Ethnicity" OR "ethnicity" OR "ethnic" OR "Hypercholesterolemia" OR "Hypercholesterolemia" OR "Hypercholesterolem*" OR "hypercholesterolaemia" OR "hypercholesterolaem*" OR "High Cholesterol" OR "Elevated Cholesterol" OR "Exercise" OR "physical activity" OR "rigorous physical activity" OR "physical activity" OR "Exercise Training" OR "Physical Activities" OR "Physical Exercise" OR "Physical Exercises" OR "lean BMI" OR ("Body Mass" AND "lean") OR "lean Body Mass Index" OR "Diabetes Mellitus" OR "Diabetes Mellitus" OR "diabetes" OR "diabetic" OR "diabetics") OR AK=("Smoking" OR "smoking" OR "smoke" OR "smoked" OR "Nicotine" OR "Nicotine" OR "Tobacco" OR "Tobacco" OR "Hypertension" OR "hypertension" OR "hypertens*" OR "High Blood Pressure" OR "elevated blood pressure" OR "Alcoholism" OR "Alcohol Abuse" OR "Alcoholism" OR "alcohol abuse" OR "Alcohol Dependence" OR "Alcohol Addiction" OR "Alcoholic Intoxication" OR "Ethanol Abuse" OR "Alcohol Use Disorder" OR "Alcohol Use Disorders" OR "oral contraceptive agent" OR "oral contraception" OR "oral contraception" OR "Oral Contraceptives" OR "Oral Contraceptive" OR "hormonal contraceptive agent" OR "hormonal contraceptive" OR "hormonal contraceptives" OR "Hormone Substitution" OR "hormone replacement therapy" OR "Estrogen Replacement Therapy" OR "Oestrogen Replacement Therapy" OR "hormone replacement" OR "Estrogen Replacement" OR "Oestrogen Replacement" OR "hormone therapy" OR "hormone therapeutic" OR "hormone treatment" OR "hormone substitution" OR exp "hormonal therapy" OR "Ethnicity" OR "ethnicity" OR "ethnic" OR "Hypercholesterolemia" OR "Hypercholesterolemia" OR "Hypercholesterolem*" OR "hypercholesterolaemia" OR "hypercholesterolaem*" OR "High Cholesterol" OR "Elevated Cholesterol" OR "Exercise" OR "physical activity" OR "rigorous physical activity" OR "physical activity" OR "Exercise Training" OR "Physical Activities" OR "Physical Exercise" OR "Physical Exercises" OR "lean BMI" OR ("Body Mass" AND "lean") OR "lean Body Mass Index" OR "Diabetes Mellitus" OR "Diabetes Mellitus" OR "diabetes" OR "diabetic" OR "diabetics") OR AB=("Smoking" OR "smoking" OR "smoke" OR "smoked" OR "Nicotine" OR "Nicotine" OR "Tobacco" OR "Tobacco" OR "Hypertension" OR "hypertension" OR "hypertens*" OR "High Blood Pressure" OR "elevated blood pressure" OR "Alcoholism" OR "Alcohol Abuse" OR "Alcoholism" OR "alcohol abuse" OR "Alcohol Dependence" OR "Alcohol Addiction" OR "Alcoholic Intoxication" OR "Ethanol Abuse" OR "Alcohol Use Disorder" OR "Alcohol Use Disorders" OR "oral contraceptive agent" OR "oral contraception" OR "oral contraception" OR "Oral Contraceptives" OR "Oral Contraceptive" OR "hormonal contraceptive agent" OR "hormonal contraceptive" OR "hormonal contraceptives" OR "Hormone Substitution" OR "hormone replacement therapy" OR "Estrogen Replacement Therapy" OR "Oestrogen Replacement Therapy" OR "hormone replacement" OR "Estrogen Replacement" OR "Oestrogen Replacement" OR "hormone therapy" OR "hormone therapeutic" OR "hormone treatment" OR "hormone substitution" OR exp "hormonal therapy" OR "Ethnicity" OR "ethnicity" OR "ethnic" OR "Hypercholesterolemia" OR "Hypercholesterolemia" OR "Hypercholesterolem*" OR "hypercholesterolaemia" OR "hypercholesterolaem*" OR "High Cholesterol" OR "Elevated Cholesterol" OR "Exercise" OR "physical activity" OR "rigorous physical activity" OR "physical activity" OR "Exercise Training" OR "Physical Activities" OR "Physical Exercise" OR "Physical Exercises" OR "lean BMI" OR ("Body Mass" AND "lean") OR "lean Body Mass Index" OR "Diabetes Mellitus" OR "Diabetes Mellitus" OR "diabetes" OR "diabetic" OR "diabetics"))) OR ((TI=("Intracranial Aneurysm" OR "Intracranial Aneurysm" OR "Intracranial Aneurysms" OR "cerebral aneurysm" OR "cerebral aneurysms" OR "Brain Aneurysm" OR "Brain Aneurysms" OR "Berry Aneurysm" OR "Berry Aneurysms" OR "Saccular Aneurysm" OR "Saccular Aneurysms" OR (("Intracranial" NEAR/3 "Aneurysm") OR ("Intracranial" NEAR/3 "Aneurysms") OR ("cerebral" NEAR/3 "aneurysm") OR ("cerebral" NEAR/3 "aneurysms") OR ("brain" NEAR/3 "aneurysm") OR ("brain" NEAR/3 "aneurysms") OR ("berry" NEAR/3 "aneurysm") OR ("berry" NEAR/3 "aneurysms") OR ("saccular" NEAR/3 "aneurysm") OR ("saccular" NEAR/3 "aneurysms"))) OR AK=("Intracranial Aneurysm" OR "Intracranial Aneurysm" OR "Intracranial Aneurysms" OR "cerebral aneurysm" OR "cerebral aneurysms" OR "Brain Aneurysm" OR "Brain Aneurysms" OR "Berry Aneurysm" OR "Berry Aneurysms" OR "Saccular Aneurysm" OR "Saccular Aneurysms" OR (("Intracranial" NEAR/3 "Aneurysm") OR ("Intracranial" NEAR/3 "Aneurysms") OR ("cerebral" NEAR/3 "aneurysm") OR ("cerebral" NEAR/3 "aneurysms") OR ("brain" NEAR/3 "aneurysm") OR ("brain" NEAR/3 "aneurysms") OR ("berry" NEAR/3 "aneurysm") OR ("berry" NEAR/3 "aneurysms") OR ("saccular" NEAR/3 "aneurysm") OR ("saccular" NEAR/3 "aneurysms"))) OR AB=("Intracranial Aneurysm" OR "Intracranial Aneurysm" OR "Intracranial Aneurysms" OR "cerebral aneurysm" OR "cerebral aneurysms" OR "Brain Aneurysm" OR "Brain Aneurysms" OR "Berry Aneurysm" OR "Berry Aneurysms" OR "Saccular Aneurysm" OR "Saccular Aneurysms" OR (("Intracranial" NEAR/3 "Aneurysm") OR ("Intracranial" NEAR/3 "Aneurysms") OR ("cerebral" NEAR/3 "aneurysm") OR ("cerebral" NEAR/3 "aneurysms") OR ("brain" NEAR/3 "aneurysm") OR ("brain" NEAR/3 "aneurysms") OR ("berry" NEAR/3 "aneurysm") OR ("berry" NEAR/3 "aneurysms") OR ("saccular" NEAR/3 "aneurysm") OR ("saccular" NEAR/3 "aneurysms")))) AND (TI=("unruptured" OR "un ruptured" OR "unruptur*" OR "un ruptur*" OR "incidental" OR "incidental*") OR AK=("unruptured" OR "un ruptured" OR "unruptur*" OR "un ruptur*" OR "incidental" OR "incidental*") OR AB=("unruptured" OR "un ruptured" OR "unruptur*" OR "un ruptur*" OR "incidental" OR "incidental*")) AND (TI=("Smoking" OR "smoking" OR "smoke" OR "smoked" OR "Nicotine" OR "Nicotine" OR "Tobacco" OR "Tobacco" OR "Hypertension" OR "hypertension" OR "hypertens*" OR "High Blood Pressure" OR "elevated blood pressure" OR "Alcoholism" OR "Alcohol Abuse" OR "Alcoholism" OR "alcohol abuse" OR "Alcohol Dependence" OR "Alcohol Addiction" OR "Alcoholic Intoxication" OR "Ethanol Abuse" OR "Alcohol Use Disorder" OR "Alcohol Use Disorders" OR "oral contraceptive agent" OR "oral contraception" OR "oral contraception" OR "Oral Contraceptives" OR "Oral Contraceptive" OR "hormonal contraceptive agent" OR "hormonal contraceptive" OR "hormonal contraceptives" OR "Hormone Substitution" OR "hormone replacement therapy" OR "Estrogen Replacement Therapy" OR "Oestrogen Replacement Therapy" OR "hormone replacement" OR "Estrogen Replacement" OR "Oestrogen Replacement" OR "hormone therapy" OR "hormone therapeutic" OR "hormone treatment" OR "hormone substitution" OR exp "hormonal therapy" OR "Ethnicity" OR "ethnicity" OR "ethnic" OR "Hypercholesterolemia" OR "Hypercholesterolemia" OR "Hypercholesterolem*" OR "hypercholesterolaemia" OR "hypercholesterolaem*" OR "High Cholesterol" OR "Elevated Cholesterol" OR "Exercise" OR "physical activity" OR "rigorous physical activity" OR "physical activity" OR "Exercise Training" OR "Physical Activities" OR "Physical Exercise" OR "Physical Exercises" OR "lean BMI" OR ("Body Mass" AND "lean") OR "lean Body Mass Index" OR "Diabetes Mellitus" OR "Diabetes Mellitus" OR "diabetes" OR "diabetic" OR "diabetics") OR AK=("Smoking" OR "smoking" OR "smoke" OR "smoked" OR "Nicotine" OR "Nicotine" OR "Tobacco" OR "Tobacco" OR "Hypertension" OR "hypertension" OR "hypertens*" OR "High Blood Pressure" OR "elevated blood pressure" OR "Alcoholism" OR "Alcohol Abuse" OR "Alcoholism" OR "alcohol abuse" OR "Alcohol Dependence" OR "Alcohol Addiction" OR "Alcoholic Intoxication" OR "Ethanol Abuse" OR "Alcohol Use Disorder" OR "Alcohol Use Disorders" OR "oral contraceptive agent" OR "oral contraception" OR "oral contraception" OR "Oral Contraceptives" OR "Oral Contraceptive" OR "hormonal contraceptive agent" OR "hormonal contraceptive" OR "hormonal contraceptives" OR "Hormone Substitution" OR "hormone replacement therapy" OR "Estrogen Replacement Therapy" OR "Oestrogen Replacement Therapy" OR "hormone replacement" OR "Estrogen Replacement" OR "Oestrogen Replacement" OR "hormone therapy" OR "hormone therapeutic" OR "hormone treatment" OR "hormone substitution" OR exp "hormonal therapy" OR "Ethnicity" OR "ethnicity" OR "ethnic" OR "Hypercholesterolemia" OR "Hypercholesterolemia" OR "Hypercholesterolem*" OR "hypercholesterolaemia" OR "hypercholesterolaem*" OR "High Cholesterol" OR "Elevated Cholesterol" OR "Exercise" OR "physical activity" OR "rigorous physical activity" OR "physical activity" OR "Exercise Training" OR "Physical Activities" OR "Physical Exercise" OR "Physical Exercises" OR "lean BMI" OR ("Body Mass" AND "lean") OR "lean Body Mass Index" OR "Diabetes Mellitus" OR "Diabetes Mellitus" OR "diabetes" OR "diabetic" OR "diabetics") OR AB=("Smoking" OR "smoking" OR "smoke" OR "smoked" OR "Nicotine" OR "Nicotine" OR "Tobacco" OR "Tobacco" OR "Hypertension" OR "hypertension" OR "hypertens*" OR "High Blood Pressure" OR "elevated blood pressure" OR "Alcoholism" OR "Alcohol Abuse" OR "Alcoholism" OR "alcohol abuse" OR "Alcohol Dependence" OR "Alcohol Addiction" OR "Alcoholic Intoxication" OR "Ethanol Abuse" OR "Alcohol Use Disorder" OR "Alcohol Use Disorders" OR "oral contraceptive agent" OR "oral contraception" OR "oral contraception" OR "Oral Contraceptives" OR "Oral Contraceptive" OR "hormonal contraceptive agent" OR "hormonal contraceptive" OR "hormonal contraceptives" OR "Hormone Substitution" OR "hormone replacement therapy" OR "Estrogen Replacement Therapy" OR "Oestrogen Replacement Therapy" OR "hormone replacement" OR "Estrogen Replacement" OR "Oestrogen Replacement" OR "hormone therapy" OR "hormone therapeutic" OR "hormone treatment" OR "hormone substitution" OR exp "hormonal therapy" OR "Ethnicity" OR "ethnicity" OR "ethnic" OR "Hypercholesterolemia" OR "Hypercholesterolemia" OR "Hypercholesterolem*" OR "hypercholesterolaemia" OR "hypercholesterolaem*" OR "High Cholesterol" OR "Elevated Cholesterol" OR "Exercise" OR "physical activity" OR "rigorous physical activity" OR "physical activity" OR "Exercise Training" OR "Physical Activities" OR "Physical Exercise" OR "Physical Exercises" OR "lean BMI" OR ("Body Mass" AND "lean") OR "lean Body Mass Index" OR "Diabetes Mellitus" OR "Diabetes Mellitus" OR "diabetes" OR "diabetic" OR "diabetics")))) AND TS=("Case Control Study" OR "case control" OR "case controlled" OR "case control*" OR "Prospective Study" OR "Prospective" OR "Prospectiv*" OR "Longitudinal Study" OR "Longitudinal" OR "Longitudinal*" OR "Follow Up" OR "Follow-Up" OR "Retrospective Study" OR "Retrospective" OR "Retrospectiv*" OR "Cohort Analysis" OR "Cohort" OR "Cohorts") AND LA=("english") NOT (TI=("veterinary" OR "rabbit" OR "rabbits" OR "animal" OR "animals" OR "mouse" OR "mice" OR "rodent" OR "rodents" OR "rat" OR "rats" OR "pig" OR "pigs" OR "porcine" OR "horse" OR "horses" OR "equine" OR "cow" OR "cows" OR "bovine" OR "goat" OR "goats" OR "sheep" OR "ovine" OR "canine" OR "dog" OR "dogs" OR "feline" OR "cat" OR "cats") OR AK=("veterinary" OR "rabbit" OR "rabbits" OR "animal" OR "animals" OR "mouse" OR "mice" OR "rodent" OR "rodents" OR "rat" OR "rats" OR "pig" OR "pigs" OR "porcine" OR "horse" OR "horses" OR "equine" OR "cow" OR "cows" OR "bovine" OR "goat" OR "goats" OR "sheep" OR "ovine" OR "canine" OR "dog" OR "dogs" OR "feline" OR "cat" OR "cats")) NOT (TI="Case Report" OR AK="case report") NOT DT=("meeting abstract")) OR ((((TI=("Subarachnoid Hemorrhage" OR "Subarachnoid Hemorrhage" OR "Subarachnoid Hemorrhages" OR "Subarachnoid Hemorrhag*" OR "Subarachnoid Haemorrhage" OR "Subarachnoid Haemorrhages" OR "Subarachnoid Haemorrhag*" OR "Subarachnoid Bleeding" OR "Subarachnoidal Hemorrhage" OR "Subarachnoidal Hemorrhages" OR "Subarachnoidal Hemorrhag*" OR "Subarachnoidal Haemorrhage" OR "Subarachnoidal Haemorrhages" OR "Subarachnoidal Haemorrhag*" OR "Subarachnoidal Bleeding" OR (("Subarachnoid" NEAR/3 "Hemorrhage") OR ("Subarachnoid" NEAR/3 "Hemorrhages") OR ("Subarachnoid" NEAR/3 "Haemorrhage") OR ("Subarachnoid" NEAR/3 "Haemorrhages") OR ("Subarachnoid" NEAR/3 "Bleeding") OR ("Subarachnoidal" NEAR/3 "Hemorrhage") OR ("Subarachnoidal" NEAR/3 "Hemorrhages") OR ("Subarachnoidal" NEAR/3 "Haemorrhage") OR ("Subarachnoidal" NEAR/3 "Haemorrhages") OR ("Subarachnoidal" NEAR/3 "Bleeding")) OR "hemorrhagic stroke" OR "hemorrhagic strokes" OR "haemorrhagic stroke" OR "haemorrhagic strokes" OR (("hemorrhagic" NEAR/3 "stroke") OR ("hemorrhagic" NEAR/3 "strokes") OR ("haemorrhagic" NEAR/3 "stroke") OR ("haemorrhagic" NEAR/3 "strokes"))) OR AK=("Subarachnoid Hemorrhage" OR "Subarachnoid Hemorrhage" OR "Subarachnoid Hemorrhages" OR "Subarachnoid Hemorrhag*" OR "Subarachnoid Haemorrhage" OR "Subarachnoid Haemorrhages" OR "Subarachnoid Haemorrhag*" OR "Subarachnoid Bleeding" OR "Subarachnoidal Hemorrhage" OR "Subarachnoidal Hemorrhages" OR "Subarachnoidal Hemorrhag*" OR "Subarachnoidal Haemorrhage" OR "Subarachnoidal Haemorrhages" OR "Subarachnoidal Haemorrhag*" OR "Subarachnoidal Bleeding" OR (("Subarachnoid" NEAR/3 "Hemorrhage") OR ("Subarachnoid" NEAR/3 "Hemorrhages") OR ("Subarachnoid" NEAR/3 "Haemorrhage") OR ("Subarachnoid" NEAR/3 "Haemorrhages") OR ("Subarachnoid" NEAR/3 "Bleeding") OR ("Subarachnoidal" NEAR/3 "Hemorrhage") OR ("Subarachnoidal" NEAR/3 "Hemorrhages") OR ("Subarachnoidal" NEAR/3 "Haemorrhage") OR ("Subarachnoidal" NEAR/3 "Haemorrhages") OR ("Subarachnoidal" NEAR/3 "Bleeding")) OR "hemorrhagic stroke" OR "hemorrhagic strokes" OR "haemorrhagic stroke" OR "haemorrhagic strokes" OR (("hemorrhagic" NEAR/3 "stroke") OR ("hemorrhagic" NEAR/3 "strokes") OR ("haemorrhagic" NEAR/3 "stroke") OR ("haemorrhagic" NEAR/3 "strokes"))) OR AB=("Subarachnoid Hemorrhage" OR "Subarachnoid Hemorrhage" OR "Subarachnoid Hemorrhages" OR "Subarachnoid Hemorrhag*" OR "Subarachnoid Haemorrhage" OR "Subarachnoid Haemorrhages" OR "Subarachnoid Haemorrhag*" OR "Subarachnoid Bleeding" OR "Subarachnoidal Hemorrhage" OR "Subarachnoidal Hemorrhages" OR "Subarachnoidal Hemorrhag*" OR "Subarachnoidal Haemorrhage" OR "Subarachnoidal Haemorrhages" OR "Subarachnoidal Haemorrhag*" OR "Subarachnoidal Bleeding" OR (("Subarachnoid" NEAR/3 "Hemorrhage") OR ("Subarachnoid" NEAR/3 "Hemorrhages") OR ("Subarachnoid" NEAR/3 "Haemorrhage") OR ("Subarachnoid" NEAR/3 "Haemorrhages") OR ("Subarachnoid" NEAR/3 "Bleeding") OR ("Subarachnoidal" NEAR/3 "Hemorrhage") OR ("Subarachnoidal" NEAR/3 "Hemorrhages") OR ("Subarachnoidal" NEAR/3 "Haemorrhage") OR ("Subarachnoidal" NEAR/3 "Haemorrhages") OR ("Subarachnoidal" NEAR/3 "Bleeding")) OR "hemorrhagic stroke" OR "hemorrhagic strokes" OR "haemorrhagic stroke" OR "haemorrhagic strokes" OR (("hemorrhagic" NEAR/3 "stroke") OR ("hemorrhagic" NEAR/3 "strokes") OR ("haemorrhagic" NEAR/3 "stroke") OR ("haemorrhagic" NEAR/3 "strokes")))) AND TI=("Smoking" OR "smoking" OR "smoke" OR "smoked" OR "Nicotine" OR "Nicotine" OR "Tobacco" OR "Tobacco" OR "Hypertension" OR "hypertension" OR "hypertens*" OR "High Blood Pressure" OR "elevated blood pressure" OR "Alcoholism" OR "Alcohol Abuse" OR "Alcoholism" OR "alcohol abuse" OR "Alcohol Dependence" OR "Alcohol Addiction" OR "Alcoholic Intoxication" OR "Ethanol Abuse" OR "Alcohol Use Disorder" OR "Alcohol Use Disorders" OR "oral contraceptive agent" OR "oral contraception" OR "oral contraception" OR "Oral Contraceptives" OR "Oral Contraceptive" OR "hormonal contraceptive agent" OR "hormonal contraceptive" OR "hormonal contraceptives" OR "Hormone Substitution" OR "hormone replacement therapy" OR "Estrogen Replacement Therapy" OR "Oestrogen Replacement Therapy" OR "hormone replacement" OR "Estrogen Replacement" OR "Oestrogen Replacement" OR "hormone therapy" OR "hormone therapeutic" OR "hormone treatment" OR "hormone substitution" OR "hormonal therapy" OR "Ethnicity" OR "ethnicity" OR "ethnic" OR "Hypercholesterolemia" OR "Hypercholesterolemia" OR "Hypercholesterolem*" OR "hypercholesterolaemia" OR "hypercholesterolaem*" OR "High Cholesterol" OR "Elevated Cholesterol" OR "Exercise" OR "physical activity" OR "rigorous physical activity" OR "physical activity" OR "Exercise Training" OR "Physical Activities" OR "Physical Exercise" OR "Physical Exercises" OR "lean BMI" OR ("Body Mass" AND "lean") OR "lean Body Mass Index" OR "Diabetes Mellitus" OR "Diabetes Mellitus" OR "diabetes" OR "diabetic" OR "diabetics")) OR (TI=("Subarachnoid Hemorrhage" OR "Subarachnoid Hemorrhage" OR "Subarachnoid Hemorrhages" OR "Subarachnoid Hemorrhag*" OR "Subarachnoid Haemorrhage" OR "Subarachnoid Haemorrhages" OR "Subarachnoid Haemorrhag*" OR "Subarachnoid Bleeding" OR "Subarachnoidal Hemorrhage" OR "Subarachnoidal Hemorrhages" OR "Subarachnoidal Hemorrhag*" OR "Subarachnoidal Haemorrhage" OR "Subarachnoidal Haemorrhages" OR "Subarachnoidal Haemorrhag*" OR "Subarachnoidal Bleeding" OR (("Subarachnoid" NEAR/3 "Hemorrhage") OR ("Subarachnoid" NEAR/3 "Hemorrhages") OR ("Subarachnoid" NEAR/3 "Haemorrhage") OR ("Subarachnoid" NEAR/3 "Haemorrhages") OR ("Subarachnoid" NEAR/3 "Bleeding") OR ("Subarachnoidal" NEAR/3 "Hemorrhage") OR ("Subarachnoidal" NEAR/3 "Hemorrhages") OR ("Subarachnoidal" NEAR/3 "Haemorrhage") OR ("Subarachnoidal" NEAR/3 "Haemorrhages") OR ("Subarachnoidal" NEAR/3 "Bleeding")) OR "hemorrhagic stroke" OR "hemorrhagic strokes" OR "haemorrhagic stroke" OR "haemorrhagic strokes" OR (("hemorrhagic" NEAR/3 "stroke") OR ("hemorrhagic" NEAR/3 "strokes") OR ("haemorrhagic" NEAR/3 "stroke") OR ("haemorrhagic" NEAR/3 "strokes"))) AND (TI=("Smoking" OR "smoking" OR "smoke" OR "smoked" OR "Nicotine" OR "Nicotine" OR "Tobacco" OR "Tobacco" OR "Hypertension" OR "hypertension" OR "hypertens*" OR "High Blood Pressure" OR "elevated blood pressure" OR "Alcoholism" OR "Alcohol Abuse" OR "Alcoholism" OR "alcohol abuse" OR "Alcohol Dependence" OR "Alcohol Addiction" OR "Alcoholic Intoxication" OR "Ethanol Abuse" OR "Alcohol Use Disorder" OR "Alcohol Use Disorders" OR "oral contraceptive agent" OR "oral contraception" OR "oral contraception" OR "Oral Contraceptives" OR "Oral Contraceptive" OR "hormonal contraceptive agent" OR "hormonal contraceptive" OR "hormonal contraceptives" OR "Hormone Substitution" OR "hormone replacement therapy" OR "Estrogen Replacement Therapy" OR "Oestrogen Replacement Therapy" OR "hormone replacement" OR "Estrogen Replacement" OR "Oestrogen Replacement" OR "hormone therapy" OR "hormone therapeutic" OR "hormone treatment" OR "hormone substitution" OR "hormonal therapy" OR "Ethnicity" OR "ethnicity" OR "ethnic" OR "Hypercholesterolemia" OR "Hypercholesterolemia" OR "Hypercholesterolem*" OR "hypercholesterolaemia" OR "hypercholesterolaem*" OR "High Cholesterol" OR "Elevated Cholesterol" OR "Exercise" OR "physical activity" OR "rigorous physical activity" OR "physical activity" OR "Exercise Training" OR "Physical Activities" OR "Physical Exercise" OR "Physical Exercises" OR "lean BMI" OR ("Body Mass" AND "lean") OR "lean Body Mass Index" OR "Diabetes Mellitus" OR "Diabetes Mellitus" OR "diabetes" OR "diabetic" OR "diabetics") OR AK=("Smoking" OR "smoking" OR "smoke" OR "smoked" OR "Nicotine" OR "Nicotine" OR "Tobacco" OR "Tobacco" OR "Hypertension" OR "hypertension" OR "hypertens*" OR "High Blood Pressure" OR "elevated blood pressure" OR "Alcoholism" OR "Alcohol Abuse" OR "Alcoholism" OR "alcohol abuse" OR "Alcohol Dependence" OR "Alcohol Addiction" OR "Alcoholic Intoxication" OR "Ethanol Abuse" OR "Alcohol Use Disorder" OR "Alcohol Use Disorders" OR "oral contraceptive agent" OR "oral contraception" OR "oral contraception" OR "Oral Contraceptives" OR "Oral Contraceptive" OR "hormonal contraceptive agent" OR "hormonal contraceptive" OR "hormonal contraceptives" OR "Hormone Substitution" OR "hormone replacement therapy" OR "Estrogen Replacement Therapy" OR "Oestrogen Replacement Therapy" OR "hormone replacement" OR "Estrogen Replacement" OR "Oestrogen Replacement" OR "hormone therapy" OR "hormone therapeutic" OR "hormone treatment" OR "hormone substitution" OR "hormonal therapy" OR "Ethnicity" OR "ethnicity" OR "ethnic" OR "Hypercholesterolemia" OR "Hypercholesterolemia" OR "Hypercholesterolem*" OR "hypercholesterolaemia" OR "hypercholesterolaem*" OR "High Cholesterol" OR "Elevated Cholesterol" OR "Exercise" OR "physical activity" OR "rigorous physical activity" OR "physical activity" OR "Exercise Training" OR "Physical Activities" OR "Physical Exercise" OR "Physical Exercises" OR "lean BMI" OR ("Body Mass" AND "lean") OR "lean Body Mass Index" OR "Diabetes Mellitus" OR "Diabetes Mellitus" OR "diabetes" OR "diabetic" OR "diabetics") OR AB=("Smoking" OR "smoking" OR "smoke" OR "smoked" OR "Nicotine" OR "Nicotine" OR "Tobacco" OR "Tobacco" OR "Hypertension" OR "hypertension" OR "hypertens*" OR "High Blood Pressure" OR "elevated blood pressure" OR "Alcoholism" OR "Alcohol Abuse" OR "Alcoholism" OR "alcohol abuse" OR "Alcohol Dependence" OR "Alcohol Addiction" OR "Alcoholic Intoxication" OR "Ethanol Abuse" OR "Alcohol Use Disorder" OR "Alcohol Use Disorders" OR "oral contraceptive agent" OR "oral contraception" OR "oral contraception" OR "Oral Contraceptives" OR "Oral Contraceptive" OR "hormonal contraceptive agent" OR "hormonal contraceptive" OR "hormonal contraceptives" OR "Hormone Substitution" OR "hormone replacement therapy" OR "Estrogen Replacement Therapy" OR "Oestrogen Replacement Therapy" OR "hormone replacement" OR "Estrogen Replacement" OR "Oestrogen Replacement" OR "hormone therapy" OR "hormone therapeutic" OR "hormone treatment" OR "hormone substitution" OR "hormonal therapy" OR "Ethnicity" OR "ethnicity" OR "ethnic" OR "Hypercholesterolemia" OR "Hypercholesterolemia" OR "Hypercholesterolem*" OR "hypercholesterolaemia" OR "hypercholesterolaem*" OR "High Cholesterol" OR "Elevated Cholesterol" OR "Exercise" OR "physical activity" OR "rigorous physical activity" OR "physical activity" OR "Exercise Training" OR "Physical Activities" OR "Physical Exercise" OR "Physical Exercises" OR "lean BMI" OR ("Body Mass" AND "lean") OR "lean Body Mass Index" OR "Diabetes Mellitus" OR "Diabetes Mellitus" OR "diabetes" OR "diabetic" OR "diabetics"))) OR ((TI=(("Subarachnoid Hemorrhage" AND "Aneurysm") OR "Subarachnoid Hemorrhage" OR "aneurysmal Subarachnoid Hemorrhages" OR "aneurysmal Subarachnoid Hemorrhag*" OR "aneurysmal Subarachnoid Haemorrhage" OR "aneurysmal Subarachnoid Haemorrhages" OR "aneurysmal Subarachnoid Haemorrhag*" OR "aneurysmal Subarachnoid Bleeding" OR "aneurysmal Subarachnoidal Hemorrhage" OR "aneurysmal Subarachnoidal Hemorrhages" OR "aneurysmal Subarachnoidal Hemorrhag*" OR "aneurysmal Subarachnoidal Haemorrhage" OR "aneurysmal Subarachnoidal Haemorrhages" OR "aneurysmal Subarachnoidal Haemorrhag*" OR "aneurysmal Subarachnoidal Bleeding" OR (( "aneurysm*" NEAR/3 "subarachnoid" NEAR/3 "Hemorrhage") OR ( "aneurysm*" NEAR/3 "subarachnoid" NEAR/3 "Hemorrhages") OR ( "aneurysm*" NEAR/3 "subarachnoid" NEAR/3 "Haemorrhage") OR ( "aneurysm*" NEAR/3 "subarachnoid" NEAR/3 "Haemorrhages") OR ( "aneurysm*" NEAR/3 "subarachnoid" NEAR/3 "Bleeding") OR ("Subarachnoidal" NEAR/3 "Hemorrhage") OR ("Subarachnoidal" NEAR/3 "Hemorrhages") OR ("Subarachnoidal" NEAR/3 "Haemorrhage") OR ("Subarachnoidal" NEAR/3 "Haemorrhages") OR ("Subarachnoidal" NEAR/3 "Bleeding"))) OR AK=(("Subarachnoid Hemorrhage" AND "Aneurysm") OR "Subarachnoid Hemorrhage" OR "aneurysmal Subarachnoid Hemorrhages" OR "aneurysmal Subarachnoid Hemorrhag*" OR "aneurysmal Subarachnoid Haemorrhage" OR "aneurysmal Subarachnoid Haemorrhages" OR "aneurysmal Subarachnoid Haemorrhag*" OR "aneurysmal Subarachnoid Bleeding" OR "aneurysmal Subarachnoidal Hemorrhage" OR "aneurysmal Subarachnoidal Hemorrhages" OR "aneurysmal Subarachnoidal Hemorrhag*" OR "aneurysmal Subarachnoidal Haemorrhage" OR "aneurysmal Subarachnoidal Haemorrhages" OR "aneurysmal Subarachnoidal Haemorrhag*" OR "aneurysmal Subarachnoidal Bleeding" OR (( "aneurysm*" NEAR/3 "subarachnoid" NEAR/3 "Hemorrhage") OR ( "aneurysm*" NEAR/3 "subarachnoid" NEAR/3 "Hemorrhages") OR ( "aneurysm*" NEAR/3 "subarachnoid" NEAR/3 "Haemorrhage") OR ( "aneurysm*" NEAR/3 "subarachnoid" NEAR/3 "Haemorrhages") OR ( "aneurysm*" NEAR/3 "subarachnoid" NEAR/3 "Bleeding") OR ("Subarachnoidal" NEAR/3 "Hemorrhage") OR ("Subarachnoidal" NEAR/3 "Hemorrhages") OR ("Subarachnoidal" NEAR/3 "Haemorrhage") OR ("Subarachnoidal" NEAR/3 "Haemorrhages") OR ("Subarachnoidal" NEAR/3 "Bleeding"))) OR AB=(("Subarachnoid Hemorrhage" AND "Aneurysm") OR "Subarachnoid Hemorrhage" OR "aneurysmal Subarachnoid Hemorrhages" OR "aneurysmal Subarachnoid Hemorrhag*" OR "aneurysmal Subarachnoid Haemorrhage" OR "aneurysmal Subarachnoid Haemorrhages" OR "aneurysmal Subarachnoid Haemorrhag*" OR "aneurysmal Subarachnoid Bleeding" OR "aneurysmal Subarachnoidal Hemorrhage" OR "aneurysmal Subarachnoidal Hemorrhages" OR "aneurysmal Subarachnoidal Hemorrhag*" OR "aneurysmal Subarachnoidal Haemorrhage" OR "aneurysmal Subarachnoidal Haemorrhages" OR "aneurysmal Subarachnoidal Haemorrhag*" OR "aneurysmal Subarachnoidal Bleeding" OR (( "aneurysm*" NEAR/3 "subarachnoid" NEAR/3 "Hemorrhage") OR ( "aneurysm*" NEAR/3 "subarachnoid" NEAR/3 "Hemorrhages") OR ( "aneurysm*" NEAR/3 "subarachnoid" NEAR/3 "Haemorrhage") OR ( "aneurysm*" NEAR/3 "subarachnoid" NEAR/3 "Haemorrhages") OR ( "aneurysm*" NEAR/3 "subarachnoid" NEAR/3 "Bleeding") OR ("Subarachnoidal" NEAR/3 "Hemorrhage") OR ("Subarachnoidal" NEAR/3 "Hemorrhages") OR ("Subarachnoidal" NEAR/3 "Haemorrhage") OR ("Subarachnoidal" NEAR/3 "Haemorrhages") OR ("Subarachnoidal" NEAR/3 "Bleeding")))) AND (TI=("Smoking" OR "smoking" OR "smoke" OR "smoked" OR "Nicotine" OR "Nicotine" OR "Tobacco" OR "Tobacco" OR "Hypertension" OR "hypertension" OR "hypertens*" OR "High Blood Pressure" OR "elevated blood pressure" OR "Alcoholism" OR "Alcohol Abuse" OR "Alcoholism" OR "alcohol abuse" OR "Alcohol Dependence" OR "Alcohol Addiction" OR "Alcoholic Intoxication" OR "Ethanol Abuse" OR "Alcohol Use Disorder" OR "Alcohol Use Disorders" OR "oral contraceptive agent" OR "oral contraception" OR "oral contraception" OR "Oral Contraceptives" OR "Oral Contraceptive" OR "hormonal contraceptive agent" OR "hormonal contraceptive" OR "hormonal contraceptives" OR "Hormone Substitution" OR "hormone replacement therapy" OR "Estrogen Replacement Therapy" OR "Oestrogen Replacement Therapy" OR "hormone replacement" OR "Estrogen Replacement" OR "Oestrogen Replacement" OR "hormone therapy" OR "hormone therapeutic" OR "hormone treatment" OR "hormone substitution" OR "hormonal therapy" OR "Ethnicity" OR "ethnicity" OR "ethnic" OR "Hypercholesterolemia" OR "Hypercholesterolemia" OR "Hypercholesterolem*" OR "hypercholesterolaemia" OR "hypercholesterolaem*" OR "High Cholesterol" OR "Elevated Cholesterol" OR "Exercise" OR "physical activity" OR "rigorous physical activity" OR "physical activity" OR "Exercise Training" OR "Physical Activities" OR "Physical Exercise" OR "Physical Exercises" OR "lean BMI" OR ("Body Mass" AND "lean") OR "lean Body Mass Index" OR "Diabetes Mellitus" OR "Diabetes Mellitus" OR "diabetes" OR "diabetic" OR "diabetics") OR AK=("Smoking" OR "smoking" OR "smoke" OR "smoked" OR "Nicotine" OR "Nicotine" OR "Tobacco" OR "Tobacco" OR "Hypertension" OR "hypertension" OR "hypertens*" OR "High Blood Pressure" OR "elevated blood pressure" OR "Alcoholism" OR "Alcohol Abuse" OR "Alcoholism" OR "alcohol abuse" OR "Alcohol Dependence" OR "Alcohol Addiction" OR "Alcoholic Intoxication" OR "Ethanol Abuse" OR "Alcohol Use Disorder" OR "Alcohol Use Disorders" OR "oral contraceptive agent" OR "oral contraception" OR "oral contraception" OR "Oral Contraceptives" OR "Oral Contraceptive" OR "hormonal contraceptive agent" OR "hormonal contraceptive" OR "hormonal contraceptives" OR "Hormone Substitution" OR "hormone replacement therapy" OR "Estrogen Replacement Therapy" OR "Oestrogen Replacement Therapy" OR "hormone replacement" OR "Estrogen Replacement" OR "Oestrogen Replacement" OR "hormone therapy" OR "hormone therapeutic" OR "hormone treatment" OR "hormone substitution" OR "hormonal therapy" OR "Ethnicity" OR "ethnicity" OR "ethnic" OR "Hypercholesterolemia" OR "Hypercholesterolemia" OR "Hypercholesterolem*" OR "hypercholesterolaemia" OR "hypercholesterolaem*" OR "High Cholesterol" OR "Elevated Cholesterol" OR "Exercise" OR "physical activity" OR "rigorous physical activity" OR "physical activity" OR "Exercise Training" OR "Physical Activities" OR "Physical Exercise" OR "Physical Exercises" OR "lean BMI" OR ("Body Mass" AND "lean") OR "lean Body Mass Index" OR "Diabetes Mellitus" OR "Diabetes Mellitus" OR "diabetes" OR "diabetic" OR "diabetics") OR AB=("Smoking" OR "smoking" OR "smoke" OR "smoked" OR "Nicotine" OR "Nicotine" OR "Tobacco" OR "Tobacco" OR "Hypertension" OR "hypertension" OR "hypertens*" OR "High Blood Pressure" OR "elevated blood pressure" OR "Alcoholism" OR "Alcohol Abuse" OR "Alcoholism" OR "alcohol abuse" OR "Alcohol Dependence" OR "Alcohol Addiction" OR "Alcoholic Intoxication" OR "Ethanol Abuse" OR "Alcohol Use Disorder" OR "Alcohol Use Disorders" OR "oral contraceptive agent" OR "oral contraception" OR "oral contraception" OR "Oral Contraceptives" OR "Oral Contraceptive" OR "hormonal contraceptive agent" OR "hormonal contraceptive" OR "hormonal contraceptives" OR "Hormone Substitution" OR "hormone replacement therapy" OR "Estrogen Replacement Therapy" OR "Oestrogen Replacement Therapy" OR "hormone replacement" OR "Estrogen Replacement" OR "Oestrogen Replacement" OR "hormone therapy" OR "hormone therapeutic" OR "hormone treatment" OR "hormone substitution" OR "hormonal therapy" OR "Ethnicity" OR "ethnicity" OR "ethnic" OR "Hypercholesterolemia" OR "Hypercholesterolemia" OR "Hypercholesterolem*" OR "hypercholesterolaemia" OR "hypercholesterolaem*" OR "High Cholesterol" OR "Elevated Cholesterol" OR "Exercise" OR "physical activity" OR "rigorous physical activity" OR "physical activity" OR "Exercise Training" OR "Physical Activities" OR "Physical Exercise" OR "Physical Exercises" OR "lean BMI" OR ("Body Mass" AND "lean") OR "lean Body Mass Index" OR "Diabetes Mellitus" OR "Diabetes Mellitus" OR "diabetes" OR "diabetic" OR "diabetics")))) AND TS=("Case Control Study" OR "case control" OR "case controlled" OR "case control*" OR "Prospective Study" OR "Prospective" OR "Prospectiv*" OR "Longitudinal Study" OR "Longitudinal" OR "Longitudinal*" OR "Follow Up" OR "Follow-Up" OR "Retrospective Study" OR "Retrospective" OR "Retrospectiv*" OR "Cohort Analysis" OR "Cohort" OR "Cohorts") AND LA=("english") NOT (TI=("veterinary" OR "rabbit" OR "rabbits" OR "animal" OR "animals" OR "mouse" OR "mice" OR "rodent" OR "rodents" OR "rat" OR "rats" OR "pig" OR "pigs" OR "porcine" OR "horse" OR "horses" OR "equine" OR "cow" OR "cows" OR "bovine" OR "goat" OR "goats" OR "sheep" OR "ovine" OR "canine" OR "dog" OR "dogs" OR "feline" OR "cat" OR "cats") OR AK=("veterinary" OR "rabbit" OR "rabbits" OR "animal" OR "animals" OR "mouse" OR "mice" OR "rodent" OR "rodents" OR "rat" OR "rats" OR "pig" OR "pigs" OR "porcine" OR "horse" OR "horses" OR "equine" OR "cow" OR "cows" OR "bovine" OR "goat" OR "goats" OR "sheep" OR "ovine" OR "canine" OR "dog" OR "dogs" OR "feline" OR "cat" OR "cats")) NOT (TI="Case Report" OR AK="case report") NOT DT=("meeting abstract"))

**IV. Cochrane Library**

*(Final search run on 01-03-2024; 11 unique items)*

(((("Intracranial Aneurysm" OR "Intracranial Aneurysm" OR "Intracranial Aneurysms" OR "cerebral aneurysm" OR "cerebral aneurysms" OR "Brain Aneurysm" OR "Brain Aneurysms" OR "Berry Aneurysm" OR "Berry Aneurysms" OR "Saccular Aneurysm" OR "Saccular Aneurysms" OR (("Intracranial" NEAR/3 "Aneurysm") OR ("Intracranial" NEAR/3 "Aneurysms") OR ("cerebral" NEAR/3 "aneurysm") OR ("cerebral" NEAR/3 "aneurysms") OR ("brain" NEAR/3 "aneurysm") OR ("brain" NEAR/3 "aneurysms") OR ("berry" NEAR/3 "aneurysm") OR ("berry" NEAR/3 "aneurysms") OR ("saccular" NEAR/3 "aneurysm") OR ("saccular" NEAR/3 "aneurysms"))):ti,ab,kw AND ("Smoking" OR "smoking" OR "smoke" OR "smoked" OR "Nicotine" OR "Nicotine" OR "Tobacco" OR "Tobacco" OR "Hypertension" OR "hypertension" OR "hypertens*" OR "High Blood Pressure" OR "elevated blood pressure" OR "Alcoholism" OR "Alcohol Abuse" OR "Alcoholism" OR "alcohol abuse" OR "Alcohol Dependence" OR "Alcohol Addiction" OR "Alcoholic Intoxication" OR "Ethanol Abuse" OR "Alcohol Use Disorder" OR "Alcohol Use Disorders" OR "oral contraceptive agent" OR "oral contraception" OR "oral contraception" OR "Oral Contraceptives" OR "Oral Contraceptive" OR "hormonal contraceptive agent" OR "hormonal contraceptive" OR "hormonal contraceptives" OR "Hormone Substitution" OR "hormone replacement therapy" OR "Estrogen Replacement Therapy" OR "Oestrogen Replacement Therapy" OR "hormone replacement" OR "Estrogen Replacement" OR "Oestrogen Replacement" OR "hormone therapy" OR "hormone therapeutic" OR "hormone treatment" OR "hormone substitution" OR exp "hormonal therapy" OR "Ethnicity" OR "ethnicity" OR "ethnic" OR "Hypercholesterolemia" OR "Hypercholesterolemia" OR "Hypercholesterolem*" OR "hypercholesterolaemia" OR "hypercholesterolaem*" OR "High Cholesterol" OR "Elevated Cholesterol" OR "Exercise" OR "physical activity" OR "rigorous physical activity" OR "physical activity" OR "Exercise Training" OR "Physical Activities" OR "Physical Exercise" OR "Physical Exercises" OR "lean BMI" OR ("Body Mass" AND "lean") OR "lean Body Mass Index" OR "Diabetes Mellitus" OR "Diabetes Mellitus" OR "diabetes" OR "diabetic" OR "diabetics"):ti) OR (("Intracranial Aneurysm" OR "Intracranial Aneurysm" OR "Intracranial Aneurysms" OR "cerebral aneurysm" OR "cerebral aneurysms" OR "Brain Aneurysm" OR "Brain Aneurysms" OR "Berry Aneurysm" OR "Berry Aneurysms" OR "Saccular Aneurysm" OR "Saccular Aneurysms" OR (("Intracranial" NEAR/3 "Aneurysm") OR ("Intracranial" NEAR/3 "Aneurysms") OR ("cerebral" NEAR/3 "aneurysm") OR ("cerebral" NEAR/3 "aneurysms") OR ("brain" NEAR/3 "aneurysm") OR ("brain" NEAR/3 "aneurysms") OR ("berry" NEAR/3 "aneurysm") OR ("berry" NEAR/3 "aneurysms") OR ("saccular" NEAR/3 "aneurysm") OR ("saccular" NEAR/3 "aneurysms"))):ti AND ("Smoking" OR "smoking" OR "smoke" OR "smoked" OR "Nicotine" OR "Nicotine" OR "Tobacco" OR "Tobacco" OR "Hypertension" OR "hypertension" OR "hypertens*" OR "High Blood Pressure" OR "elevated blood pressure" OR "Alcoholism" OR "Alcohol Abuse" OR "Alcoholism" OR "alcohol abuse" OR "Alcohol Dependence" OR "Alcohol Addiction" OR "Alcoholic Intoxication" OR "Ethanol Abuse" OR "Alcohol Use Disorder" OR "Alcohol Use Disorders" OR "oral contraceptive agent" OR "oral contraception" OR "oral contraception" OR "Oral Contraceptives" OR "Oral Contraceptive" OR "hormonal contraceptive agent" OR "hormonal contraceptive" OR "hormonal contraceptives" OR "Hormone Substitution" OR "hormone replacement therapy" OR "Estrogen Replacement Therapy" OR "Oestrogen Replacement Therapy" OR "hormone replacement" OR "Estrogen Replacement" OR "Oestrogen Replacement" OR "hormone therapy" OR "hormone therapeutic" OR "hormone treatment" OR "hormone substitution" OR exp "hormonal therapy" OR "Ethnicity" OR "ethnicity" OR "ethnic" OR "Hypercholesterolemia" OR "Hypercholesterolemia" OR "Hypercholesterolem*" OR "hypercholesterolaemia" OR "hypercholesterolaem*" OR "High Cholesterol" OR "Elevated Cholesterol" OR "Exercise" OR "physical activity" OR "rigorous physical activity" OR "physical activity" OR "Exercise Training" OR "Physical Activities" OR "Physical Exercise" OR "Physical Exercises" OR "lean BMI" OR ("Body Mass" AND "lean") OR "lean Body Mass Index" OR "Diabetes Mellitus" OR "Diabetes Mellitus" OR "diabetes" OR "diabetic" OR "diabetics"):ti,ab,kw) OR (("Intracranial Aneurysm" OR "Intracranial Aneurysm" OR "Intracranial Aneurysms" OR "cerebral aneurysm" OR "cerebral aneurysms" OR "Brain Aneurysm" OR "Brain Aneurysms" OR "Berry Aneurysm" OR "Berry Aneurysms" OR "Saccular Aneurysm" OR "Saccular Aneurysms" OR (("Intracranial" NEAR/3 "Aneurysm") OR ("Intracranial" NEAR/3 "Aneurysms") OR ("cerebral" NEAR/3 "aneurysm") OR ("cerebral" NEAR/3 "aneurysms") OR ("brain" NEAR/3 "aneurysm") OR ("brain" NEAR/3 "aneurysms") OR ("berry" NEAR/3 "aneurysm") OR ("berry" NEAR/3 "aneurysms") OR ("saccular" NEAR/3 "aneurysm") OR ("saccular" NEAR/3 "aneurysms"))):ti,ab,kw AND ("unruptured" OR "un ruptured" OR "unruptur*" OR "un ruptur*" OR "incidental" OR "incidental*"):ti,ab,kw AND ("Smoking" OR "smoking" OR "smoke" OR "smoked" OR "Nicotine" OR "Nicotine" OR "Tobacco" OR "Tobacco" OR "Hypertension" OR "hypertension" OR "hypertens*" OR "High Blood Pressure" OR "elevated blood pressure" OR "Alcoholism" OR "Alcohol Abuse" OR "Alcoholism" OR "alcohol abuse" OR "Alcohol Dependence" OR "Alcohol Addiction" OR "Alcoholic Intoxication" OR "Ethanol Abuse" OR "Alcohol Use Disorder" OR "Alcohol Use Disorders" OR "oral contraceptive agent" OR "oral contraception" OR "oral contraception" OR "Oral Contraceptives" OR "Oral Contraceptive" OR "hormonal contraceptive agent" OR "hormonal contraceptive" OR "hormonal contraceptives" OR "Hormone Substitution" OR "hormone replacement therapy" OR "Estrogen Replacement Therapy" OR "Oestrogen Replacement Therapy" OR "hormone replacement" OR "Estrogen Replacement" OR "Oestrogen Replacement" OR "hormone therapy" OR "hormone therapeutic" OR "hormone treatment" OR "hormone substitution" OR exp "hormonal therapy" OR "Ethnicity" OR "ethnicity" OR "ethnic" OR "Hypercholesterolemia" OR "Hypercholesterolemia" OR "Hypercholesterolem*" OR "hypercholesterolaemia" OR "hypercholesterolaem*" OR "High Cholesterol" OR "Elevated Cholesterol" OR "Exercise" OR "physical activity" OR "rigorous physical activity" OR "physical activity" OR "Exercise Training" OR "Physical Activities" OR "Physical Exercise" OR "Physical Exercises" OR "lean BMI" OR ("Body Mass" AND "lean") OR "lean Body Mass Index" OR "Diabetes Mellitus" OR "Diabetes Mellitus" OR "diabetes" OR "diabetic" OR "diabetics"):ti,ab,kw)) AND ("Case Control" OR "case controlled" OR "Prospective Cohort" OR "Prospective Cohorts" OR "Cohort Study"):ti,ab,kw) OR (((("Subarachnoid Hemorrhage" OR "Subarachnoid Hemorrhage" OR "Subarachnoid Hemorrhages" OR "Subarachnoid Hemorrhag*" OR "Subarachnoid Haemorrhage" OR "Subarachnoid Haemorrhages" OR "Subarachnoid Haemorrhag*" OR "Subarachnoid Bleeding" OR "Subarachnoidal Hemorrhage" OR "Subarachnoidal Hemorrhages" OR "Subarachnoidal Hemorrhag*" OR "Subarachnoidal Haemorrhage" OR "Subarachnoidal Haemorrhages" OR "Subarachnoidal Haemorrhag*" OR "Subarachnoidal Bleeding" OR (("Subarachnoid" NEAR/3 "Hemorrhage") OR ("Subarachnoid" NEAR/3 "Hemorrhages") OR ("Subarachnoid" NEAR/3 "Haemorrhage") OR ("Subarachnoid" NEAR/3 "Haemorrhages") OR ("Subarachnoid" NEAR/3 "Bleeding") OR ("Subarachnoidal" NEAR/3 "Hemorrhage") OR ("Subarachnoidal" NEAR/3 "Hemorrhages") OR ("Subarachnoidal" NEAR/3 "Haemorrhage") OR ("Subarachnoidal" NEAR/3 "Haemorrhages") OR ("Subarachnoidal" NEAR/3 "Bleeding")) OR "hemorrhagic stroke" OR "hemorrhagic strokes" OR "haemorrhagic stroke" OR "haemorrhagic strokes" OR (("hemorrhagic" NEAR/3 "stroke") OR ("hemorrhagic" NEAR/3 "strokes") OR ("haemorrhagic" NEAR/3 "stroke") OR ("haemorrhagic" NEAR/3 "strokes"))):ti,ab,kw AND ("Smoking" OR "smoking" OR "smoke" OR "smoked" OR "Nicotine" OR "Nicotine" OR "Tobacco" OR "Tobacco" OR "Hypertension" OR "hypertension" OR "hypertens*" OR "High Blood Pressure" OR "elevated blood pressure" OR "Alcoholism" OR "Alcohol Abuse" OR "Alcoholism" OR "alcohol abuse" OR "Alcohol Dependence" OR "Alcohol Addiction" OR "Alcoholic Intoxication" OR "Ethanol Abuse" OR "Alcohol Use Disorder" OR "Alcohol Use Disorders" OR "oral contraceptive agent" OR "oral contraception" OR "oral contraception" OR "Oral Contraceptives" OR "Oral Contraceptive" OR "hormonal contraceptive agent" OR "hormonal contraceptive" OR "hormonal contraceptives" OR "Hormone Substitution" OR "hormone replacement therapy" OR "Estrogen Replacement Therapy" OR "Oestrogen Replacement Therapy" OR "hormone replacement" OR "Estrogen Replacement" OR "Oestrogen Replacement" OR "hormone therapy" OR "hormone therapeutic" OR "hormone treatment" OR "hormone substitution" OR "hormonal therapy" OR "Ethnicity" OR "ethnicity" OR "ethnic" OR "Hypercholesterolemia" OR "Hypercholesterolemia" OR "Hypercholesterolem*" OR "hypercholesterolaemia" OR "hypercholesterolaem*" OR "High Cholesterol" OR "Elevated Cholesterol" OR "Exercise" OR "physical activity" OR "rigorous physical activity" OR "physical activity" OR "Exercise Training" OR "Physical Activities" OR "Physical Exercise" OR "Physical Exercises" OR "lean BMI" OR ("Body Mass" AND "lean") OR "lean Body Mass Index" OR "Diabetes Mellitus" OR "Diabetes Mellitus" OR "diabetes" OR "diabetic" OR "diabetics"):ti) OR (("Subarachnoid Hemorrhage" OR "Subarachnoid Hemorrhage" OR "Subarachnoid Hemorrhages" OR "Subarachnoid Hemorrhag*" OR "Subarachnoid Haemorrhage" OR "Subarachnoid Haemorrhages" OR "Subarachnoid Haemorrhag*" OR "Subarachnoid Bleeding" OR "Subarachnoidal Hemorrhage" OR "Subarachnoidal Hemorrhages" OR "Subarachnoidal Hemorrhag*" OR "Subarachnoidal Haemorrhage" OR "Subarachnoidal Haemorrhages" OR "Subarachnoidal Haemorrhag*" OR "Subarachnoidal Bleeding" OR (("Subarachnoid" NEAR/3 "Hemorrhage") OR ("Subarachnoid" NEAR/3 "Hemorrhages") OR ("Subarachnoid" NEAR/3 "Haemorrhage") OR ("Subarachnoid" NEAR/3 "Haemorrhages") OR ("Subarachnoid" NEAR/3 "Bleeding") OR ("Subarachnoidal" NEAR/3 "Hemorrhage") OR ("Subarachnoidal" NEAR/3 "Hemorrhages") OR ("Subarachnoidal" NEAR/3 "Haemorrhage") OR ("Subarachnoidal" NEAR/3 "Haemorrhages") OR ("Subarachnoidal" NEAR/3 "Bleeding")) OR "hemorrhagic stroke" OR "hemorrhagic strokes" OR "haemorrhagic stroke" OR "haemorrhagic strokes" OR (("hemorrhagic" NEAR/3 "stroke") OR ("hemorrhagic" NEAR/3 "strokes") OR ("haemorrhagic" NEAR/3 "stroke") OR ("haemorrhagic" NEAR/3 "strokes"))):ti AND ("Smoking" OR "smoking" OR "smoke" OR "smoked" OR "Nicotine" OR "Nicotine" OR "Tobacco" OR "Tobacco" OR "Hypertension" OR "hypertension" OR "hypertens*" OR "High Blood Pressure" OR "elevated blood pressure" OR "Alcoholism" OR "Alcohol Abuse" OR "Alcoholism" OR "alcohol abuse" OR "Alcohol Dependence" OR "Alcohol Addiction" OR "Alcoholic Intoxication" OR "Ethanol Abuse" OR "Alcohol Use Disorder" OR "Alcohol Use Disorders" OR "oral contraceptive agent" OR "oral contraception" OR "oral contraception" OR "Oral Contraceptives" OR "Oral Contraceptive" OR "hormonal contraceptive agent" OR "hormonal contraceptive" OR "hormonal contraceptives" OR "Hormone Substitution" OR "hormone replacement therapy" OR "Estrogen Replacement Therapy" OR "Oestrogen Replacement Therapy" OR "hormone replacement" OR "Estrogen Replacement" OR "Oestrogen Replacement" OR "hormone therapy" OR "hormone therapeutic" OR "hormone treatment" OR "hormone substitution" OR "hormonal therapy" OR "Ethnicity" OR "ethnicity" OR "ethnic" OR "Hypercholesterolemia" OR "Hypercholesterolemia" OR "Hypercholesterolem*" OR "hypercholesterolaemia" OR "hypercholesterolaem*" OR "High Cholesterol" OR "Elevated Cholesterol" OR "Exercise" OR "physical activity" OR "rigorous physical activity" OR "physical activity" OR "Exercise Training" OR "Physical Activities" OR "Physical Exercise" OR "Physical Exercises" OR "lean BMI" OR ("Body Mass" AND "lean") OR "lean Body Mass Index" OR "Diabetes Mellitus" OR "Diabetes Mellitus" OR "diabetes" OR "diabetic" OR "diabetics"):ti,ab,kw) OR ((("Subarachnoid Hemorrhage" AND "Aneurysm") OR "Subarachnoid Hemorrhage" OR "aneurysmal Subarachnoid Hemorrhages" OR "aneurysmal Subarachnoid Hemorrhag*" OR "aneurysmal Subarachnoid Haemorrhage" OR "aneurysmal Subarachnoid Haemorrhages" OR "aneurysmal Subarachnoid Haemorrhag*" OR "aneurysmal Subarachnoid Bleeding" OR "aneurysmal Subarachnoidal Hemorrhage" OR "aneurysmal Subarachnoidal Hemorrhages" OR "aneurysmal Subarachnoidal Hemorrhag*" OR "aneurysmal Subarachnoidal Haemorrhage" OR "aneurysmal Subarachnoidal Haemorrhages" OR "aneurysmal Subarachnoidal Haemorrhag*" OR "aneurysmal Subarachnoidal Bleeding" OR (( "aneurysm*" NEAR/3 "subarachnoid" NEAR/3 "Hemorrhage") OR ( "aneurysm*" NEAR/3 "subarachnoid" NEAR/3 "Hemorrhages") OR ( "aneurysm*" NEAR/3 "subarachnoid" NEAR/3 "Haemorrhage") OR ( "aneurysm*" NEAR/3 "subarachnoid" NEAR/3 "Haemorrhages") OR ( "aneurysm*" NEAR/3 "subarachnoid" NEAR/3 "Bleeding") OR ("Subarachnoidal" NEAR/3 "Hemorrhage") OR ("Subarachnoidal" NEAR/3 "Hemorrhages") OR ("Subarachnoidal" NEAR/3 "Haemorrhage") OR ("Subarachnoidal" NEAR/3 "Haemorrhages") OR ("Subarachnoidal" NEAR/3 "Bleeding"))):ti,ab,kw AND ("Smoking" OR "smoking" OR "smoke" OR "smoked" OR "Nicotine" OR "Nicotine" OR "Tobacco" OR "Tobacco" OR "Hypertension" OR "hypertension" OR "hypertens*" OR "High Blood Pressure" OR "elevated blood pressure" OR "Alcoholism" OR "Alcohol Abuse" OR "Alcoholism" OR "alcohol abuse" OR "Alcohol Dependence" OR "Alcohol Addiction" OR "Alcoholic Intoxication" OR "Ethanol Abuse" OR "Alcohol Use Disorder" OR "Alcohol Use Disorders" OR "oral contraceptive agent" OR "oral contraception" OR "oral contraception" OR "Oral Contraceptives" OR "Oral Contraceptive" OR "hormonal contraceptive agent" OR "hormonal contraceptive" OR "hormonal contraceptives" OR "Hormone Substitution" OR "hormone replacement therapy" OR "Estrogen Replacement Therapy" OR "Oestrogen Replacement Therapy" OR "hormone replacement" OR "Estrogen Replacement" OR "Oestrogen Replacement" OR "hormone therapy" OR "hormone therapeutic" OR "hormone treatment" OR "hormone substitution" OR "hormonal therapy" OR "Ethnicity" OR "ethnicity" OR "ethnic" OR "Hypercholesterolemia" OR "Hypercholesterolemia" OR "Hypercholesterolem*" OR "hypercholesterolaemia" OR "hypercholesterolaem*" OR "High Cholesterol" OR "Elevated Cholesterol" OR "Exercise" OR "physical activity" OR "rigorous physical activity" OR "physical activity" OR "Exercise Training" OR "Physical Activities" OR "Physical Exercise" OR "Physical Exercises" OR "lean BMI" OR ("Body Mass" AND "lean") OR "lean Body Mass Index" OR "Diabetes Mellitus" OR "Diabetes Mellitus" OR "diabetes" OR "diabetic" OR "diabetics"):ti,ab,kw)) AND ("Case Control" OR "case controlled" OR "Prospective Cohort" OR "Prospective Cohorts" OR "Cohort Study"):ti,ab,kw)

**V. Emcare** (OVID version)

*(Final search run on 01-03-2024; 26 unique items)*

(((((exp *"Intracranial Aneurysm"/ OR "Intracranial Aneurysm".ti,ab OR "Intracranial Aneurysms".ti,ab OR "cerebral aneurysm".ti,ab OR "cerebral aneurysms".ti,ab OR "Brain Aneurysm".ti,ab OR "Brain Aneurysms".ti,ab OR "Berry Aneurysm".ti,ab OR "Berry Aneurysms".ti,ab OR "Saccular Aneurysm".ti,ab OR "Saccular Aneurysms".ti,ab OR (("Intracranial" ADJ3 "Aneurysm") OR ("Intracranial" ADJ3 "Aneurysms") OR ("cerebral" ADJ3 "aneurysm") OR ("cerebral" ADJ3 "aneurysms") OR ("brain" ADJ3 "aneurysm") OR ("brain" ADJ3 "aneurysms") OR ("berry" ADJ3 "aneurysm") OR ("berry" ADJ3 "aneurysms") OR ("saccular" ADJ3 "aneurysm") OR ("saccular" ADJ3 "aneurysms")).ti,ab) AND (exp *"Smoking"/ OR "smoking".ti OR "smoke".ti OR "smoked".ti OR exp *"Nicotine"/ OR "Nicotine".ti OR exp *"Tobacco"/ OR "Tobacco".ti OR exp *"Hypertension"/ OR "hypertension".ti OR "hypertens*".ti OR "High Blood Pressure".ti OR "elevated blood pressure".ti OR exp *"Alcoholism"/ OR exp *"Alcohol Abuse"/ OR "Alcoholism".ti OR "alcohol abuse".ti OR "Alcohol Dependence".ti OR "Alcohol Addiction".ti OR "Alcoholic Intoxication".ti OR "Ethanol Abuse".ti OR "Alcohol Use Disorder".ti OR "Alcohol Use Disorders".ti OR exp *"oral contraceptive agent"/ OR exp *"oral contraception"/ OR "oral contraception".ti OR "Oral Contraceptives".ti OR "Oral Contraceptive".ti OR "hormonal contraceptive agent"/ OR "hormonal contraceptive".ti OR "hormonal contraceptives".ti OR exp *"Hormone Substitution"/ OR "hormone replacement therapy".ti OR "Estrogen Replacement Therapy".ti OR "Oestrogen Replacement Therapy".ti OR "hormone replacement".ti OR "Estrogen Replacement".ti OR "Oestrogen Replacement".ti OR "hormone therapy".ti OR "hormone therapeutic".ti OR "hormone treatment".ti OR "hormone substitution".ti OR exp "hormonal therapy"/ OR exp *"Ethnicity"/ OR "ethnicity".ti OR "ethnic".ti OR exp *"Hypercholesterolemia"/ OR "Hypercholesterolemia".ti OR "Hypercholesterolem*".ti OR "hypercholesterolaemia".ti OR "hypercholesterolaem*".ti OR "High Cholesterol".ti OR "Elevated Cholesterol".ti OR exp *"Exercise"/ OR exp *"physical activity"/ OR "rigorous physical activity".ti OR "physical activity".ti OR "Exercise Training".ti OR "Physical Activities".ti OR "Physical Exercise".ti OR "Physical Exercises".ti OR "lean BMI".ti OR (exp *"Body Mass"/ AND "lean".ti) OR "lean Body Mass Index".ti OR exp *"Diabetes Mellitus"/ OR "Diabetes Mellitus".ti OR "diabetes".ti OR "diabetic".ti OR "diabetics".ti)) OR OR ((exp *"Intracranial Aneurysm"/ OR "Intracranial Aneurysm".ti OR "Intracranial Aneurysms".ti OR "cerebral aneurysm".ti OR "cerebral aneurysms".ti OR "Brain Aneurysm".ti OR "Brain Aneurysms".ti OR "Berry Aneurysm".ti OR "Berry Aneurysms".ti OR "Saccular Aneurysm".ti OR "Saccular Aneurysms".ti OR (("Intracranial" ADJ3 "Aneurysm") OR ("Intracranial" ADJ3 "Aneurysms") OR ("cerebral" ADJ3 "aneurysm") OR ("cerebral" ADJ3 "aneurysms") OR ("brain" ADJ3 "aneurysm") OR ("brain" ADJ3 "aneurysms") OR ("berry" ADJ3 "aneurysm") OR ("berry" ADJ3 "aneurysms") OR ("saccular" ADJ3 "aneurysm") OR ("saccular" ADJ3 "aneurysms")).ti) AND (exp *"Smoking"/ OR "smoking".ti,ab OR "smoke".ti,ab OR "smoked".ti,ab OR exp *"Nicotine"/ OR "Nicotine".ti,ab OR exp *"Tobacco"/ OR "Tobacco".ti,ab OR exp *"Hypertension"/ OR "hypertension".ti,ab OR "hypertens*".ti,ab OR "High Blood Pressure".ti,ab OR "elevated blood pressure".ti,ab OR exp *"Alcoholism"/ OR exp *"Alcohol Abuse"/ OR "Alcoholism".ti,ab OR "alcohol abuse".ti,ab OR "Alcohol Dependence".ti,ab OR "Alcohol Addiction".ti,ab OR "Alcoholic Intoxication".ti,ab OR "Ethanol Abuse".ti,ab OR "Alcohol Use Disorder".ti,ab OR "Alcohol Use Disorders".ti,ab OR exp *"oral contraceptive agent"/ OR exp *"oral contraception"/ OR "oral contraception".ti,ab OR "Oral Contraceptives".ti,ab OR "Oral Contraceptive".ti,ab OR "hormonal contraceptive agent"/ OR "hormonal contraceptive".ti,ab OR "hormonal contraceptives".ti,ab OR exp *"Hormone Substitution"/ OR "hormone replacement therapy".ti,ab OR "Estrogen Replacement Therapy".ti,ab OR "Oestrogen Replacement Therapy".ti,ab OR "hormone replacement".ti,ab OR "Estrogen Replacement".ti,ab OR "Oestrogen Replacement".ti,ab OR "hormone therapy".ti,ab OR "hormone therapeutic".ti,ab OR "hormone treatment".ti,ab OR "hormone substitution".ti,ab OR exp "hormonal therapy"/ OR exp *"Ethnicity"/ OR "ethnicity".ti,ab OR "ethnic".ti,ab OR exp *"Hypercholesterolemia"/ OR "Hypercholesterolemia".ti,ab OR "Hypercholesterolem*".ti,ab OR "hypercholesterolaemia".ti,ab OR "hypercholesterolaem*".ti,ab OR "High Cholesterol".ti,ab OR "Elevated Cholesterol".ti,ab OR exp *"Exercise"/ OR exp *"physical activity"/ OR "rigorous physical activity".ti,ab OR "physical activity".ti,ab OR "Exercise Training".ti,ab OR "Physical Activities".ti,ab OR "Physical Exercise".ti,ab OR "Physical Exercises".ti,ab OR "lean BMI".ti,ab OR (exp *"Body Mass"/ AND "lean".ti,ab) OR "lean Body Mass Index".ti,ab OR exp *"Diabetes Mellitus"/ OR "Diabetes Mellitus".ti,ab OR "diabetes".ti,ab OR "diabetic".ti,ab OR "diabetics".ti,ab)) OR ((exp *"Intracranial Aneurysm"/ OR "Intracranial Aneurysm".ti,ab OR "Intracranial Aneurysms".ti,ab OR "cerebral aneurysm".ti,ab OR "cerebral aneurysms".ti,ab OR "Brain Aneurysm".ti,ab OR "Brain Aneurysms".ti,ab OR "Berry Aneurysm".ti,ab OR "Berry Aneurysms".ti,ab OR "Saccular Aneurysm".ti,ab OR "Saccular Aneurysms".ti,ab OR (("Intracranial" ADJ3 "Aneurysm") OR ("Intracranial" ADJ3 "Aneurysms") OR ("cerebral" ADJ3 "aneurysm") OR ("cerebral" ADJ3 "aneurysms") OR ("brain" ADJ3 "aneurysm") OR ("brain" ADJ3 "aneurysms") OR ("berry" ADJ3 "aneurysm") OR ("berry" ADJ3 "aneurysms") OR ("saccular" ADJ3 "aneurysm") OR ("saccular" ADJ3 "aneurysms")).ti,ab) AND ("unruptured".ti OR "un ruptured".ti OR "unruptur*".ti OR "un ruptur*".ti OR "incidental".ti OR "incidental*".ti) AND (exp *"Smoking"/ OR "smoking".ti,ab OR "smoke".ti,ab OR "smoked".ti,ab OR exp *"Nicotine"/ OR "Nicotine".ti,ab OR exp *"Tobacco"/ OR "Tobacco".ti,ab OR exp *"Hypertension"/ OR "hypertension".ti,ab OR "hypertens*".ti,ab OR "High Blood Pressure".ti,ab OR "elevated blood pressure".ti,ab OR exp *"Alcoholism"/ OR exp *"Alcohol Abuse"/ OR "Alcoholism".ti,ab OR "alcohol abuse".ti,ab OR "Alcohol Dependence".ti,ab OR "Alcohol Addiction".ti,ab OR "Alcoholic Intoxication".ti,ab OR "Ethanol Abuse".ti,ab OR "Alcohol Use Disorder".ti,ab OR "Alcohol Use Disorders".ti,ab OR exp *"oral contraceptive agent"/ OR exp *"oral contraception"/ OR "oral contraception".ti,ab OR "Oral Contraceptives".ti,ab OR "Oral Contraceptive".ti,ab OR "hormonal contraceptive agent"/ OR "hormonal contraceptive".ti,ab OR "hormonal contraceptives".ti,ab OR exp *"Hormone Substitution"/ OR "hormone replacement therapy".ti,ab OR "Estrogen Replacement Therapy".ti,ab OR "Oestrogen Replacement Therapy".ti,ab OR "hormone replacement".ti,ab OR "Estrogen Replacement".ti,ab OR "Oestrogen Replacement".ti,ab OR "hormone therapy".ti,ab OR "hormone therapeutic".ti,ab OR "hormone treatment".ti,ab OR "hormone substitution".ti,ab OR exp "hormonal therapy"/ OR exp *"Ethnicity"/ OR "ethnicity".ti,ab OR "ethnic".ti,ab OR exp *"Hypercholesterolemia"/ OR "Hypercholesterolemia".ti,ab OR "Hypercholesterolem*".ti,ab OR "hypercholesterolaemia".ti,ab OR "hypercholesterolaem*".ti,ab OR "High Cholesterol".ti,ab OR "Elevated Cholesterol".ti,ab OR exp *"Exercise"/ OR exp *"physical activity"/ OR "rigorous physical activity".ti,ab OR "physical activity".ti,ab OR "Exercise Training".ti,ab OR "Physical Activities".ti,ab OR "Physical Exercise".ti,ab OR "Physical Exercises".ti,ab OR "lean BMI".ti,ab OR (exp *"Body Mass"/ AND "lean".ti,ab) OR "lean Body Mass Index".ti,ab OR exp *"Diabetes Mellitus"/ OR "Diabetes Mellitus".ti,ab OR "diabetes".ti,ab OR "diabetic".ti,ab OR "diabetics".ti,ab))) AND (exp "Case Control Study"/ OR "case control".mp OR "case controlled".mp OR "case control*".mp OR exp "Prospective Study"/ OR "Prospective".mp OR "Prospectiv*".mp OR exp "Longitudinal Study"/ OR "Longitudinal".mp OR "Longitudinal*".mp OR exp *"Follow Up"/ OR "Follow-Up".mp OR exp "Retrospective Study"/ OR "Retrospective".mp OR "Retrospectiv*".mp OR exp "Cohort Analysis"/ OR "Cohort".mp OR "Cohorts".mp) AND ("english".la) NOT (exp "Animals"/ NOT exp "Humans"/) NOT (("Case Report"/ OR "case report".ti OR (case AND (report OR reports)).jw) NOT (exp "Review"/ OR "review".ti OR "Clinical Study"/ OR exp "Clinical Trial"/ OR "trial".ti OR "RCT".ti)) OR ((((exp *"Subarachnoid Hemorrhage"/ OR "Subarachnoid Hemorrhage".ti,ab OR "Subarachnoid Hemorrhages".ti,ab OR "Subarachnoid Hemorrhag*".ti,ab OR "Subarachnoid Haemorrhage".ti,ab OR "Subarachnoid Haemorrhages".ti,ab OR "Subarachnoid Haemorrhag*".ti,ab OR "Subarachnoid Bleeding".ti,ab OR "Subarachnoidal Hemorrhage".ti,ab OR "Subarachnoidal Hemorrhages".ti,ab OR "Subarachnoidal Hemorrhag*".ti,ab OR "Subarachnoidal Haemorrhage".ti,ab OR "Subarachnoidal Haemorrhages".ti,ab OR "Subarachnoidal Haemorrhag*".ti,ab OR "Subarachnoidal Bleeding".ti,ab OR (("Subarachnoid" ADJ3 "Hemorrhage") OR ("Subarachnoid" ADJ3 "Hemorrhages") OR ("Subarachnoid" ADJ3 "Haemorrhage") OR ("Subarachnoid" ADJ3 "Haemorrhages") OR ("Subarachnoid" ADJ3 "Bleeding") OR ("Subarachnoidal" ADJ3 "Hemorrhage") OR ("Subarachnoidal" ADJ3 "Hemorrhages") OR ("Subarachnoidal" ADJ3 "Haemorrhage") OR ("Subarachnoidal" ADJ3 "Haemorrhages") OR ("Subarachnoidal" ADJ3 "Bleeding")).ti,ab OR "hemorrhagic stroke".ti,ab OR "hemorrhagic strokes".ti,ab OR "haemorrhagic stroke".ti,ab OR "haemorrhagic strokes".ti,ab OR (("hemorrhagic" ADJ3 "stroke") OR ("hemorrhagic" ADJ3 "strokes") OR ("haemorrhagic" ADJ3 "stroke") OR ("haemorrhagic" ADJ3 "strokes")).ti,ab) AND (exp *"Smoking"/ OR "smoking".ti OR "smoke".ti OR "smoked".ti OR exp *"Nicotine"/ OR "Nicotine".ti OR exp *"Tobacco"/ OR "Tobacco".ti OR exp *"Hypertension"/ OR "hypertension".ti OR "hypertens*".ti OR "High Blood Pressure".ti OR "elevated blood pressure".ti OR exp *"Alcoholism"/ OR exp *"Alcohol Abuse"/ OR "Alcoholism".ti OR "alcohol abuse".ti OR "Alcohol Dependence".ti OR "Alcohol Addiction".ti OR "Alcoholic Intoxication".ti OR "Ethanol Abuse".ti OR "Alcohol Use Disorder".ti OR "Alcohol Use Disorders".ti OR exp *"oral contraceptive agent"/ OR exp *"oral contraception"/ OR "oral contraception".ti OR "Oral Contraceptives".ti OR "Oral Contraceptive".ti OR "hormonal contraceptive agent"/ OR "hormonal contraceptive".ti OR "hormonal contraceptives".ti OR exp *"Hormone Substitution"/ OR "hormone replacement therapy".ti OR "Estrogen Replacement Therapy".ti OR "Oestrogen Replacement Therapy".ti OR "hormone replacement".ti OR "Estrogen Replacement".ti OR "Oestrogen Replacement".ti OR "hormone therapy".ti OR "hormone therapeutic".ti OR "hormone treatment".ti OR "hormone substitution".ti OR exp "hormonal therapy"/ OR exp *"Ethnicity"/ OR "ethnicity".ti OR "ethnic".ti OR exp *"Hypercholesterolemia"/ OR "Hypercholesterolemia".ti OR "Hypercholesterolem*".ti OR "hypercholesterolaemia".ti OR "hypercholesterolaem*".ti OR "High Cholesterol".ti OR "Elevated Cholesterol".ti OR exp *"Exercise"/ OR exp *"physical activity"/ OR "rigorous physical activity".ti OR "physical activity".ti OR "Exercise Training".ti OR "Physical Activities".ti OR "Physical Exercise".ti OR "Physical Exercises".ti OR "lean BMI".ti OR (exp *"Body Mass"/ AND "lean".ti) OR "lean Body Mass Index".ti OR exp *"Diabetes Mellitus"/ OR "Diabetes Mellitus".ti OR "diabetes".ti OR "diabetic".ti OR "diabetics".ti)) OR ((exp *"Subarachnoid Hemorrhage"/ OR "Subarachnoid Hemorrhage".ti OR "Subarachnoid Hemorrhages".ti OR "Subarachnoid Hemorrhag*".ti OR "Subarachnoid Haemorrhage".ti OR "Subarachnoid Haemorrhages".ti OR "Subarachnoid Haemorrhag*".ti OR "Subarachnoid Bleeding".ti OR "Subarachnoidal Hemorrhage".ti OR "Subarachnoidal Hemorrhages".ti OR "Subarachnoidal Hemorrhag*".ti OR "Subarachnoidal Haemorrhage".ti OR "Subarachnoidal Haemorrhages".ti OR "Subarachnoidal Haemorrhag*".ti OR "Subarachnoidal Bleeding".ti OR (("Subarachnoid" ADJ3 "Hemorrhage") OR ("Subarachnoid" ADJ3 "Hemorrhages") OR ("Subarachnoid" ADJ3 "Haemorrhage") OR ("Subarachnoid" ADJ3 "Haemorrhages") OR ("Subarachnoid" ADJ3 "Bleeding") OR ("Subarachnoidal" ADJ3 "Hemorrhage") OR ("Subarachnoidal" ADJ3 "Hemorrhages") OR ("Subarachnoidal" ADJ3 "Haemorrhage") OR ("Subarachnoidal" ADJ3 "Haemorrhages") OR ("Subarachnoidal" ADJ3 "Bleeding")).ti OR "hemorrhagic stroke".ti OR "hemorrhagic strokes".ti OR "haemorrhagic stroke".ti OR "haemorrhagic strokes".ti OR (("hemorrhagic" ADJ3 "stroke") OR ("hemorrhagic" ADJ3 "strokes") OR ("haemorrhagic" ADJ3 "stroke") OR ("haemorrhagic" ADJ3 "strokes")).ti) AND (exp *"Smoking"/ OR "smoking".ti,ab OR "smoke".ti,ab OR "smoked".ti,ab OR exp *"Nicotine"/ OR "Nicotine".ti,ab OR exp *"Tobacco"/ OR "Tobacco".ti,ab OR exp *"Hypertension"/ OR "hypertension".ti,ab OR "hypertens*".ti,ab OR "High Blood Pressure".ti,ab OR "elevated blood pressure".ti,ab OR exp *"Alcoholism"/ OR exp *"Alcohol Abuse"/ OR "Alcoholism".ti,ab OR "alcohol abuse".ti,ab OR "Alcohol Dependence".ti,ab OR "Alcohol Addiction".ti,ab OR "Alcoholic Intoxication".ti,ab OR "Ethanol Abuse".ti,ab OR "Alcohol Use Disorder".ti,ab OR "Alcohol Use Disorders".ti,ab OR exp *"oral contraceptive agent"/ OR exp *"oral contraception"/ OR "oral contraception".ti,ab OR "Oral Contraceptives".ti,ab OR "Oral Contraceptive".ti,ab OR "hormonal contraceptive agent"/ OR "hormonal contraceptive".ti,ab OR "hormonal contraceptives".ti,ab OR exp *"Hormone Substitution"/ OR "hormone replacement therapy".ti,ab OR "Estrogen Replacement Therapy".ti,ab OR "Oestrogen Replacement Therapy".ti,ab OR "hormone replacement".ti,ab OR "Estrogen Replacement".ti,ab OR "Oestrogen Replacement".ti,ab OR "hormone therapy".ti,ab OR "hormone therapeutic".ti,ab OR "hormone treatment".ti,ab OR "hormone substitution".ti,ab OR exp "hormonal therapy"/ OR exp *"Ethnicity"/ OR "ethnicity".ti,ab OR "ethnic".ti,ab OR exp *"Hypercholesterolemia"/ OR "Hypercholesterolemia".ti,ab OR "Hypercholesterolem*".ti,ab OR "hypercholesterolaemia".ti,ab OR "hypercholesterolaem*".ti,ab OR "High Cholesterol".ti,ab OR "Elevated Cholesterol".ti,ab OR exp *"Exercise"/ OR exp *"physical activity"/ OR "rigorous physical activity".ti,ab OR "physical activity".ti,ab OR "Exercise Training".ti,ab OR "Physical Activities".ti,ab OR "Physical Exercise".ti,ab OR "Physical Exercises".ti,ab OR "lean BMI".ti,ab OR (exp *"Body Mass"/ AND "lean".ti,ab) OR "lean Body Mass Index".ti,ab OR exp *"Diabetes Mellitus"/ OR "Diabetes Mellitus".ti,ab OR "diabetes".ti,ab OR "diabetic".ti,ab OR "diabetics".ti,ab)) OR (((exp *"Subarachnoid Hemorrhage"/ AND exp *"Aneurysm"/) OR "Subarachnoid Hemorrhage".ti,ab OR "aneurysmal Subarachnoid Hemorrhages".ti,ab OR "aneurysmal Subarachnoid Hemorrhag*".ti,ab OR "aneurysmal Subarachnoid Haemorrhage".ti,ab OR "aneurysmal Subarachnoid Haemorrhages".ti,ab OR "aneurysmal Subarachnoid Haemorrhag*".ti,ab OR "aneurysmal Subarachnoid Bleeding".ti,ab OR "aneurysmal Subarachnoidal Hemorrhage".ti,ab OR "aneurysmal Subarachnoidal Hemorrhages".ti,ab OR "aneurysmal Subarachnoidal Hemorrhag*".ti,ab OR "aneurysmal Subarachnoidal Haemorrhage".ti,ab OR "aneurysmal Subarachnoidal Haemorrhages".ti,ab OR "aneurysmal Subarachnoidal Haemorrhag*".ti,ab OR "aneurysmal Subarachnoidal Bleeding".ti,ab OR (( "aneurysm*" ADJ3 "subarachnoid" ADJ3 "Hemorrhage") OR ( "aneurysm*" ADJ3 "subarachnoid" ADJ3 "Hemorrhages") OR ( "aneurysm*" ADJ3 "subarachnoid" ADJ3 "Haemorrhage") OR ( "aneurysm*" ADJ3 "subarachnoid" ADJ3 "Haemorrhages") OR ( "aneurysm*" ADJ3 "subarachnoid" ADJ3 "Bleeding") OR ("Subarachnoidal" ADJ3 "Hemorrhage") OR ("Subarachnoidal" ADJ3 "Hemorrhages") OR ("Subarachnoidal" ADJ3 "Haemorrhage") OR ("Subarachnoidal" ADJ3 "Haemorrhages") OR ("Subarachnoidal" ADJ3 "Bleeding")).ti,ab) AND (exp *"Smoking"/ OR "smoking".ti,ab OR "smoke".ti,ab OR "smoked".ti,ab OR exp *"Nicotine"/ OR "Nicotine".ti,ab OR exp *"Tobacco"/ OR "Tobacco".ti,ab OR exp *"Hypertension"/ OR "hypertension".ti,ab OR "hypertens*".ti,ab OR "High Blood Pressure".ti,ab OR "elevated blood pressure".ti,ab OR exp *"Alcoholism"/ OR exp *"Alcohol Abuse"/ OR "Alcoholism".ti,ab OR "alcohol abuse".ti,ab OR "Alcohol Dependence".ti,ab OR "Alcohol Addiction".ti,ab OR "Alcoholic Intoxication".ti,ab OR "Ethanol Abuse".ti,ab OR "Alcohol Use Disorder".ti,ab OR "Alcohol Use Disorders".ti,ab OR exp *"oral contraceptive agent"/ OR exp *"oral contraception"/ OR "oral contraception".ti,ab OR "Oral Contraceptives".ti,ab OR "Oral Contraceptive".ti,ab OR "hormonal contraceptive agent"/ OR "hormonal contraceptive".ti,ab OR "hormonal contraceptives".ti,ab OR exp *"Hormone Substitution"/ OR "hormone replacement therapy".ti,ab OR "Estrogen Replacement Therapy".ti,ab OR "Oestrogen Replacement Therapy".ti,ab OR "hormone replacement".ti,ab OR "Estrogen Replacement".ti,ab OR "Oestrogen Replacement".ti,ab OR "hormone therapy".ti,ab OR "hormone therapeutic".ti,ab OR "hormone treatment".ti,ab OR "hormone substitution".ti,ab OR exp "hormonal therapy"/ OR exp *"Ethnicity"/ OR "ethnicity".ti,ab OR "ethnic".ti,ab OR exp *"Hypercholesterolemia"/ OR "Hypercholesterolemia".ti,ab OR "Hypercholesterolem*".ti,ab OR "hypercholesterolaemia".ti,ab OR "hypercholesterolaem*".ti,ab OR "High Cholesterol".ti,ab OR "Elevated Cholesterol".ti,ab OR exp *"Exercise"/ OR exp *"physical activity"/ OR "rigorous physical activity".ti,ab OR "physical activity".ti,ab OR "Exercise Training".ti,ab OR "Physical Activities".ti,ab OR "Physical Exercise".ti,ab OR "Physical Exercises".ti,ab OR "lean BMI".ti,ab OR (exp *"Body Mass"/ AND "lean".ti,ab) OR "lean Body Mass Index".ti,ab OR exp *"Diabetes Mellitus"/ OR "Diabetes Mellitus".ti,ab OR "diabetes".ti,ab OR "diabetic".ti,ab OR "diabetics".ti,ab))) AND (exp "Case Control Study"/ OR "case control".mp OR "case controlled".mp OR "case control*".mp OR exp "Prospective Study"/ OR "Prospective".mp OR "Prospectiv*".mp OR exp "Longitudinal Study"/ OR "Longitudinal".mp OR "Longitudinal*".mp OR exp *"Follow Up"/ OR "Follow-Up".mp OR exp "Retrospective Study"/ OR "Retrospective".mp OR "Retrospectiv*".mp OR exp "Cohort Analysis"/ OR "Cohort".mp OR "Cohorts".mp) AND ("english".la) NOT (exp "Animals"/ NOT exp "Humans"/) NOT (("Case Report"/ OR "case report".ti OR (case AND (report OR reports)).jw) NOT (exp "Review"/ OR "review".ti OR "Clinical Study"/ OR exp "Clinical Trial"/ OR "trial".ti OR "RCT".ti)))))

**S2. Selection criteria**

We excluded studies that specifically focused on multiple or fusiform aneurysms. However, studies in which these aneurysm types constituted <30% of a broader group of predominantly saccular aneurysms were included.

**S3. Data extraction**

Data retrieved from each study included (1) author, country and year of publication; (2) study design; (3) size of study population, including the number of UIA cases and sex distribution; (4) age range or mean age of study population; (5) study period; (6) method used to confirm UIA presence, and (7) risk factors examined. For the reported risk factors in each study odds ratios (ORs) with 95% CIs or raw patient numbers were obtained. Sex-specific estimates were also extracted when available. In case only raw patient numbers were provided (categorized by exposure vs. non-exposure, and outcome vs. non-outcome), we calculated ORs with corresponding 95% CIs ourselves.

**S4. Protocol - Risk of Bias Assessment (Customized version of the Newcastle-Ottawa Scale)**

Studies were classified as low or high risk of bias for each element of the risk of bias analysis. Unclear risk of bias was reported when data on this item in the risk of bias assessment was either missing or unclear.

(1) Validation of diagnosis

Low risk of bias was defined as a confirmed UIA diagnosis by use of neuroimaging (either computed tomography angiography, magnetic resonance angiography or conventional angiography) or by autopsy. High risk of bias was defined as assessment of the presence of UIA by use of International Classification of Diseases codes.

(2) Assessment of risk factors

Low risk of bias was considered as risk factors being assessed through structured interviews using standardized questionnaires on lifestyle and medical history. High risk of bias included risk factor assessment through collection of data from electronic health records.

(3) Adjustment for confounding

Adjustment for at least age was considered as low risk of bias. No adjustments made for confounding or no adjustment made for age was classified as high risk of bias.

(4) Generalizability

Studies including patient populations representative of the general population were considered low risk of bias, while those with restricted populations (such as hospital-based cohorts) were categorized as high risk of bias.

**S5. Unclassifiable risk factor definitions not included in meta-analysis**

Some risk factors could not be included in the meta-analysis due to unclassifiable definitions.

Smoking

Two studies (Vlak et al., 2013; Matsukawa et al., 2014) categorized smoking as current vs. non-current, which did not match our standardized definition of ever vs. never smoking. In multivariable analysis, Vlak et al. reported an OR of 3.00 (95% CI 2.00 – 4.50), while Matsukawa et al. reported an OR of 3.50 (95% CI 0.86 – 3.14) for current smoking.

BMI

Four studies (Chen et al., 2011; Li et al.,2013; Matsukawa et al., 2014; and Igase., 2021) reported only mean BMI values for both UIA patients and controls, without using reference categories. In a multiple variable model, Cras et al. reported an OR of 0.97 (95% CI 0.93 – 1.02) per kg/m^2^, which could not be dichotomized according to our standardized definitions.

Alcohol use

One study (Cras et al., 2020) reported an OR of 0.99 (95% CI 0.97 – 1.01) per additional glass of alcohol consumed per day, based on a multivariable model.

Hyperlipidemia

One study (Yoon et al., 2019) reported only mean low-density lipoprotein cholesterol levels for cases and controls, without specifying a reference category.

Physical activity

One study (Cras et al., 2020) reported physical activity per 10 Metabolic Equivalent of Task (MET) hours per week and found an OR of 1.01 (95% CI 0.96 – 1.04).

**S6. Studies excluded after full-text review**

| **Author and year of publication** | **Reason for exclusion** |
| --- | --- |
| Wiebers 1981^1^ | No control group |
| De La Monte 1985^2^ | Data on UIA cases combined with aSAH cases |
| Ohaegbulam 1990^3^ | No control group |
| Juvela 2001^4^ | Data on UIA cases combined with aSAH cases |
| Juvela 2002^5^ | Data on UIA cases combined with aSAH cases |
| Gu 2006^6^ | Specific patient group |
| Nguyen 2009^7^ | Familial IA cases as control group |
| Marbacher 2012^8^ | Data on UIA cases combined with aSAH cases |
| Lindgren 2014^9^ | aSAH cases as control group |
| Lai 2014^10^ | Data on UIA cases combined with aSAH cases |
| Wang 2015^11^ | Data on UIA cases combined with aSAH cases |
| Guan 2016^12^ | Specific patient group |
| Lindgren 2016^13^ | Overlap of study cohort with study cohort of other study |
| Duman 2017^14^ | No control group |
| Kim 2018^15^ | Overlap of study cohort with study cohort of other study |
| Hostettler 2018^16^ | aSAH cases as control group |
| Rosi 2018^17^ | Data on UIA cases combined with aSAH cases |
| Krzyzewski 2018^18^ | No control group |
| Vourla 2019^19^ | Data on UIA cases combined with aSAH cases |
| Wang 2020^20^ | Specific patient group |
| Schatlo 2019^21^ | Data on UIA cases combined with aSAH cases |
| Rosi 2019^22^ | No control group |
| Majewska 2020^23^ | No cohort, case-control, or cross-sectional study design |
| Haase 2020^24^ | aSAH cases as control group |
| Han 2022^25^ | aSAH cases as control group |
| Missori 2022^26^ | Data on UIA cases combined with aSAH cases |
| Morel 2022^27^ | aSAH cases as control group |
| Wahood 2022^28^ | aSAH cases as control group |
| Walchli 2023^29^ | aSAH cases as control group |
| Javed 2023^30^ | Specific patient group |
| Jung 2023^31^ | Non-specified risk factor |
| Zeng 2023^32^ | No cohort, case-control, or cross-sectional study design |

Studies are shown in ascending chronological order.

UIA = unruptured intracranial aneurysm; aSAH = aneurysmal subarachnoid hemorrhage.

**S7. Risk of bias assessment of included studies**

| **Author, year** | **Validation of diagnosis** | **Assessment of risk factors** | **Adjustment for confounders** | **Generalizability** |
| --- | --- | --- | --- | --- |
| Horikoshi, 2002 | Low | High | Low | High |
| Inagawa, 2010 | Low | High | Possible | High |
| Chen, 2011 | Possible | Low | High | High |
| Vlak, 2013 | Low | Low | High | High |
| Li, 2013 | Low | Low | High | Low |
| Jing Li, 2014 | Low | Possible | High | High |
| Matsukawa, 2014 | Low | Possible | High | High |
| Zhang, 2015 | Low | Low | High | High |
| Kang, 2015 | Low | High | Low | Possible |
| Kim Tackeun, 2016 | High | Low | High | Low |
| Atchaneeyasakul, 2018 | Low | High | High | High |
| Imaizumi, 2018 | Low | Possible | Low | Low |
| Yoon, 2019 | Low | High | Low | High |
| Müller, 2019 | Low | Low | Low | Low |
| Cras, 2020 | Low | Low | Low | Low |
| Ogilvy, 2020 (single center) | Low | Possible | Low | High |
| Ogilvy, 2020 (multicenter) | Low | Possible | High | High |
| Kim Jae Ho, 2021 | Low | High | High | Low |
| Igase, 2021 | Low | Low | Possible | High |
| Räisänen, 2022 | Low | High | Low | High |
| Park, 2023 | High | High | High | Low |

**S8. Publication bias funnel plots**

Funnel plots for A) smoking; B) hypertension; C) diabetes; and D) any lipid abnormality, showing no clear asymmetry. Egger’s regression tests similarly did not indicate small-study effects: t = 0.95, df = 16, p = 0.36 for smoking; t= -1.19, df = 18, p = 0.25 for hypertension; t = -0.70, df = 14, p = 0.49 for diabetes; and

t = -0.80, df = 11, p = 0.44 for any lipid abnormality, together suggesting low risk of publication bias.


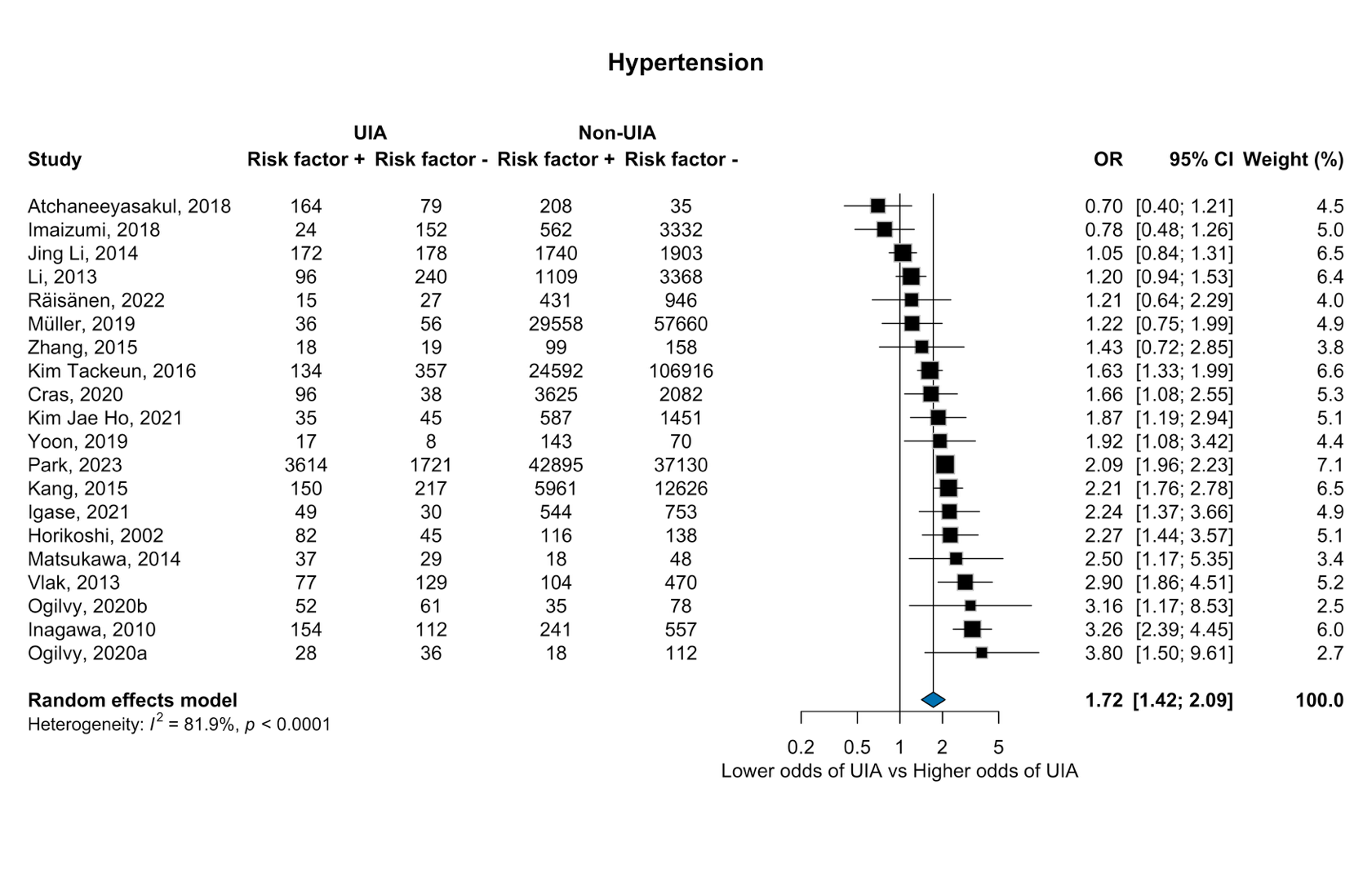
**S9. Forest plot of hypertension (present vs absent)**

Ogilvy, 2020a refers to the single center study; Ogilvy, 2020b to the multicenter study.

**S10. Forest plot of smoking (ever vs never)**


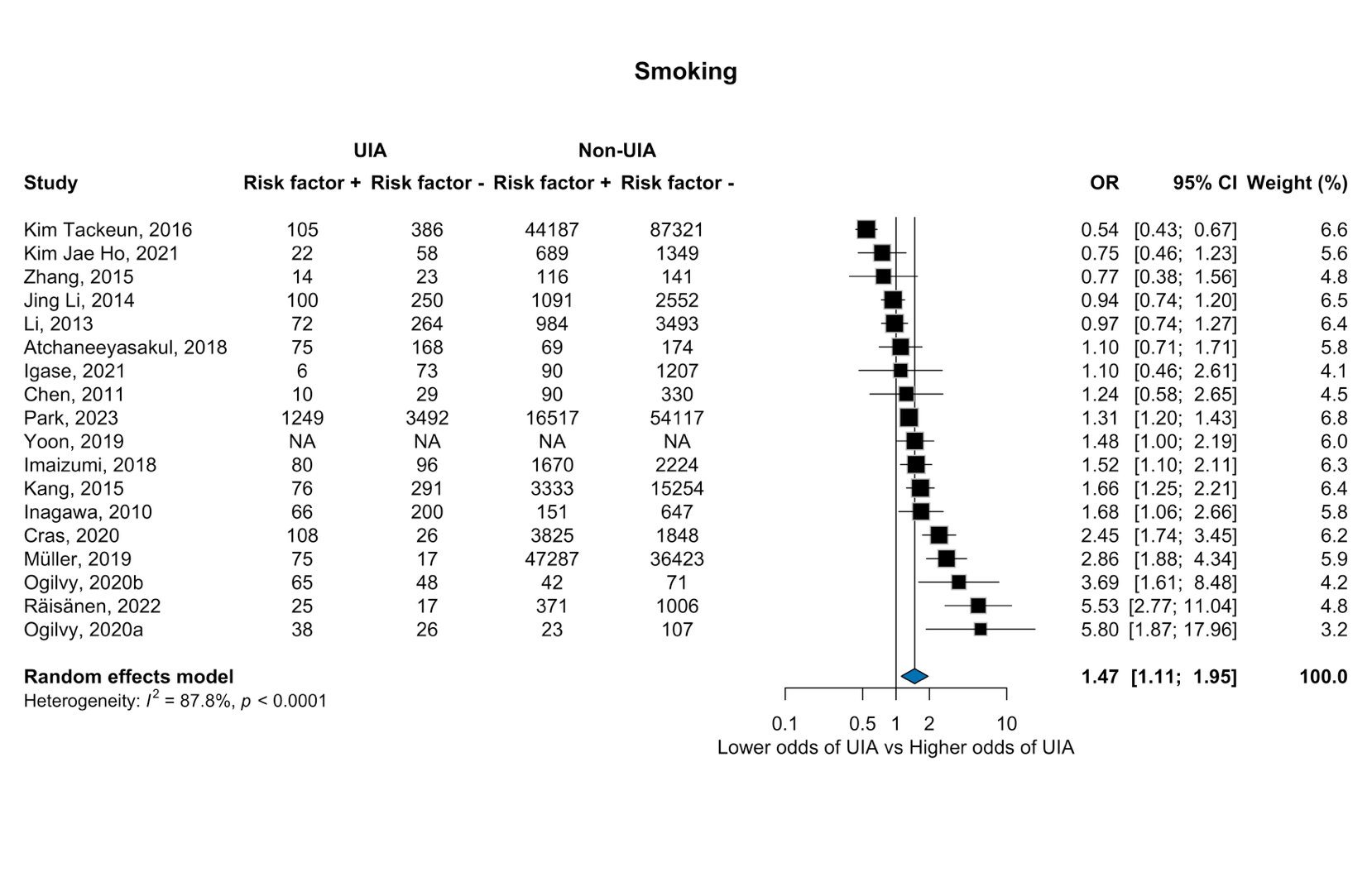


Ogilvy, 2020a refers to the single center study; Ogilvy, 2020b to the multicenter study.

NA = not available; the numbers of participants with and without the risk factor were not reported in the study. The study was still included in the forest plot using the reported odds ratio.


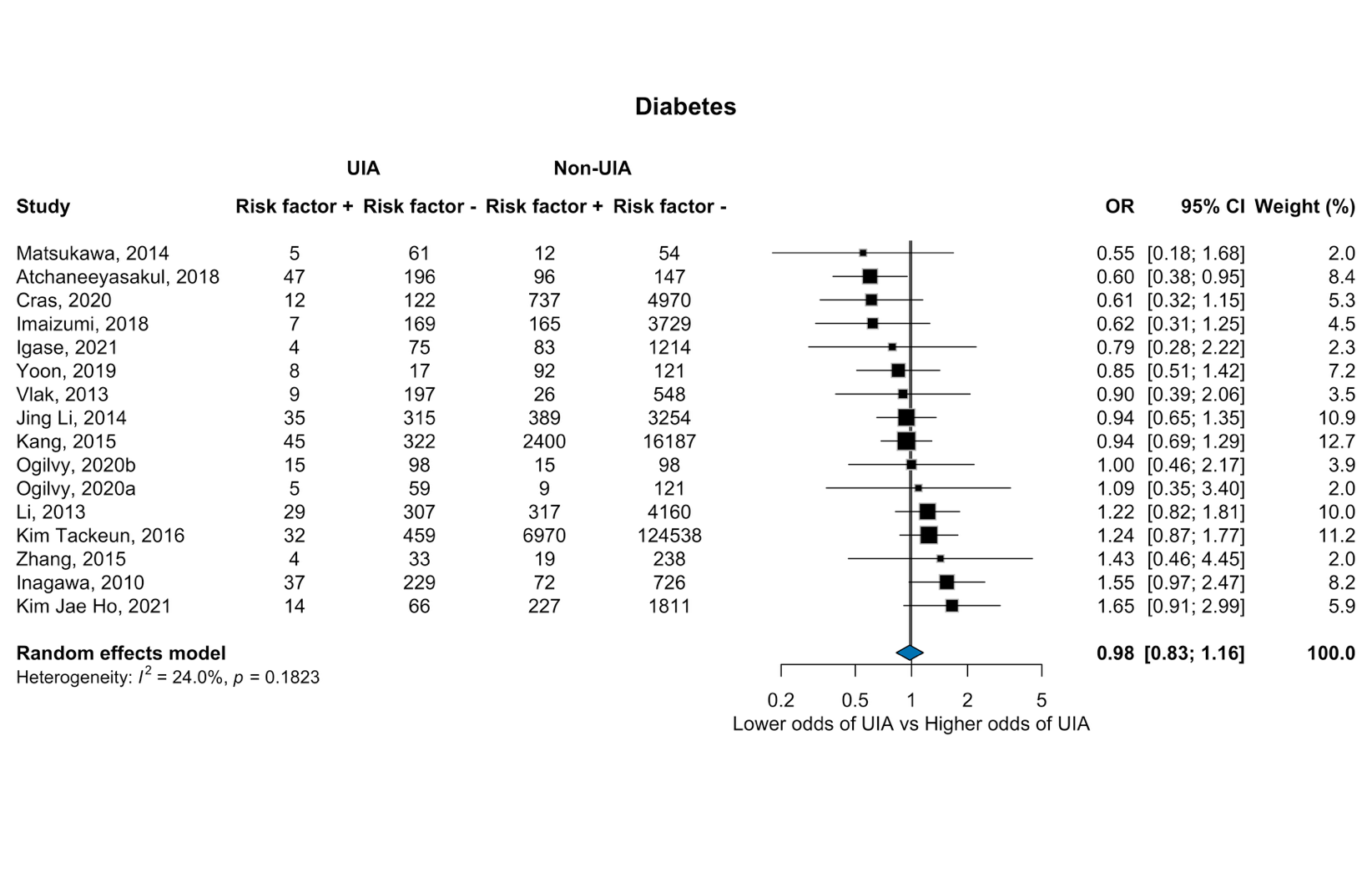
**S11. Forest plot of diabetes (present vs absent)**

Ogilvy, 2020a refers to the single center study; Ogilvy, 2020b to the multicenter study.

**S12. Forest plot of alcohol use (yes vs no)**

**
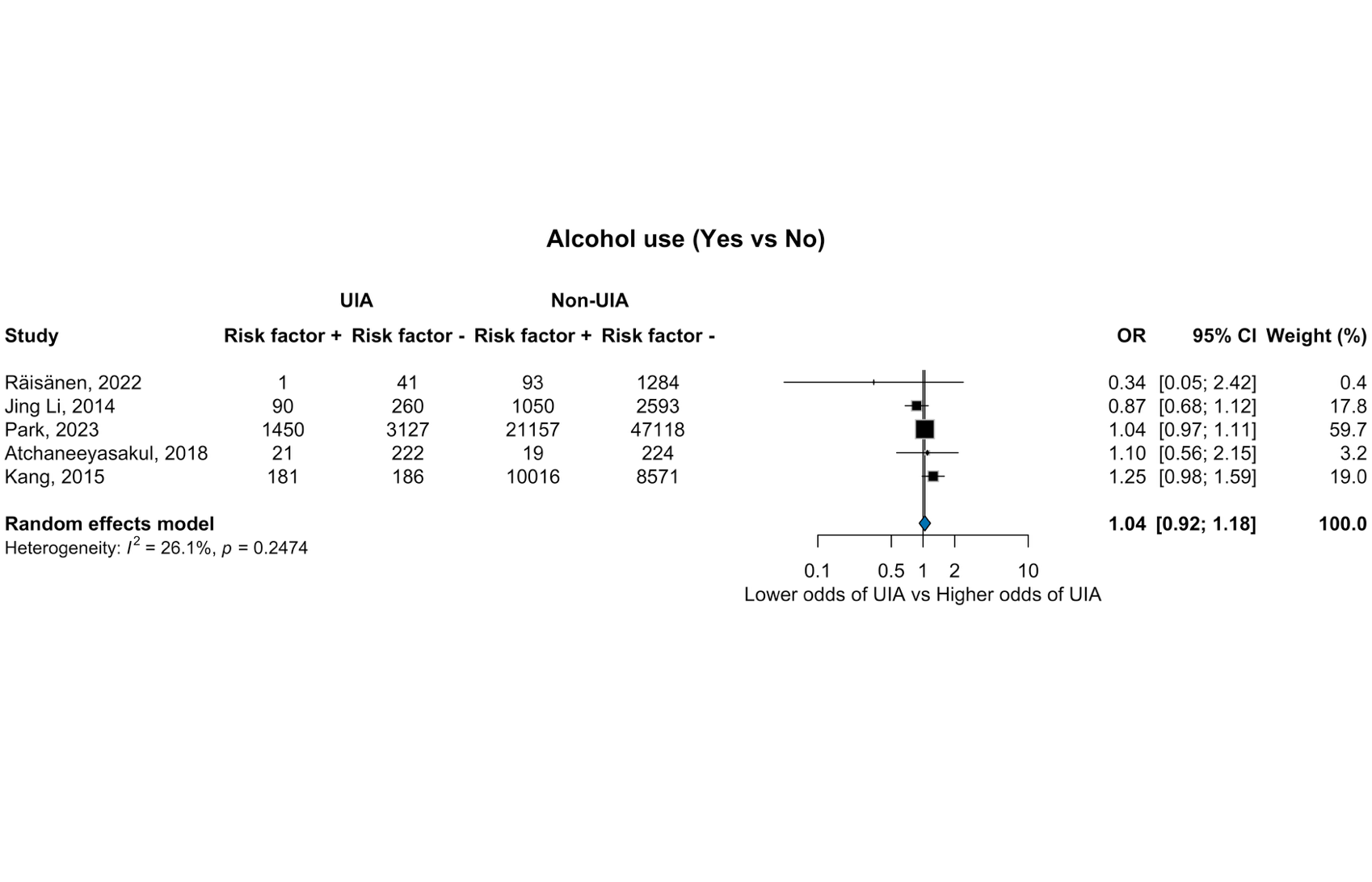
**

**S13. Forest plot of alcohol use (regular vs non-regular)**

**
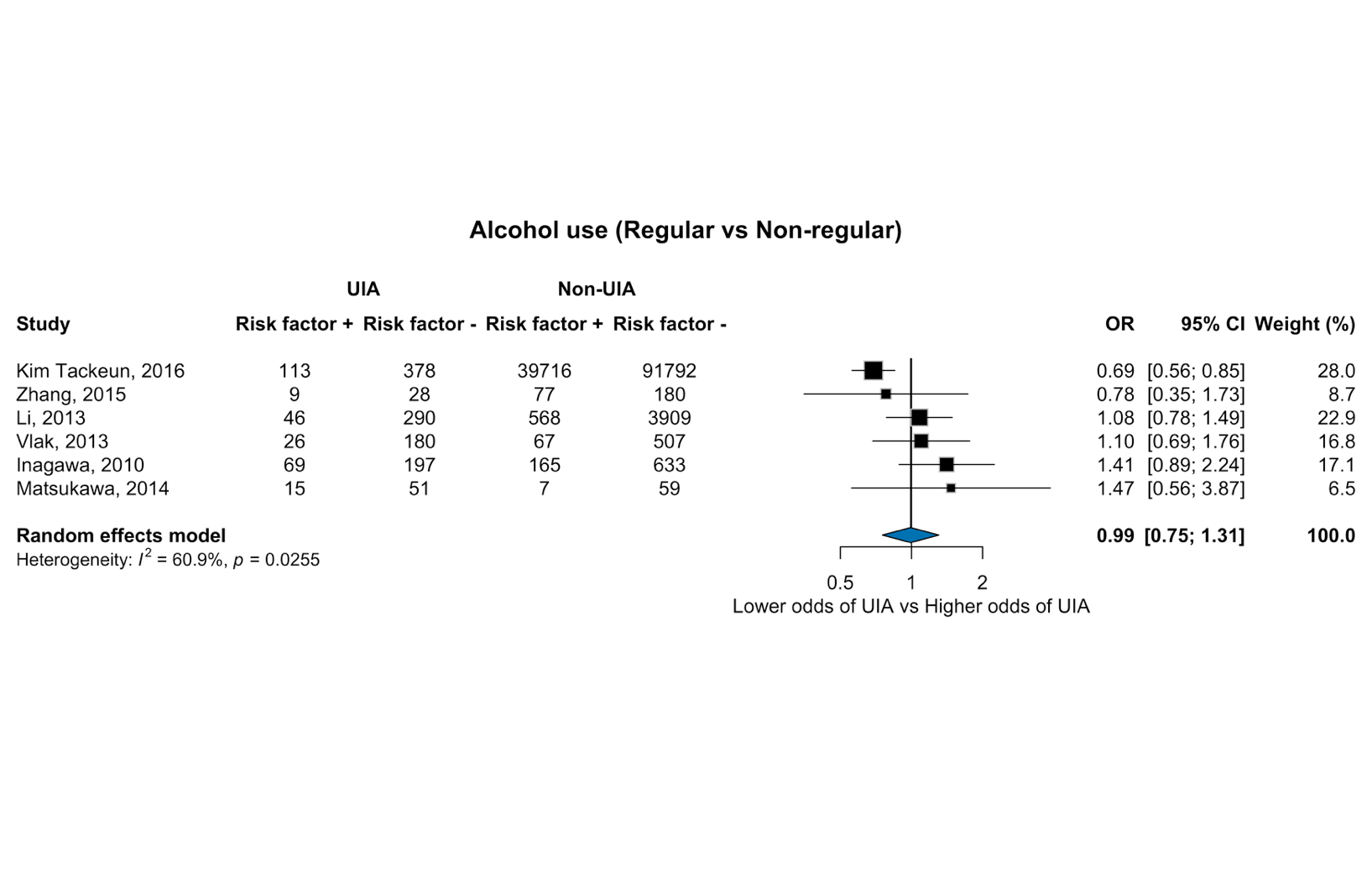
**

**S14. Forest plots of any lipid abnormality**

**
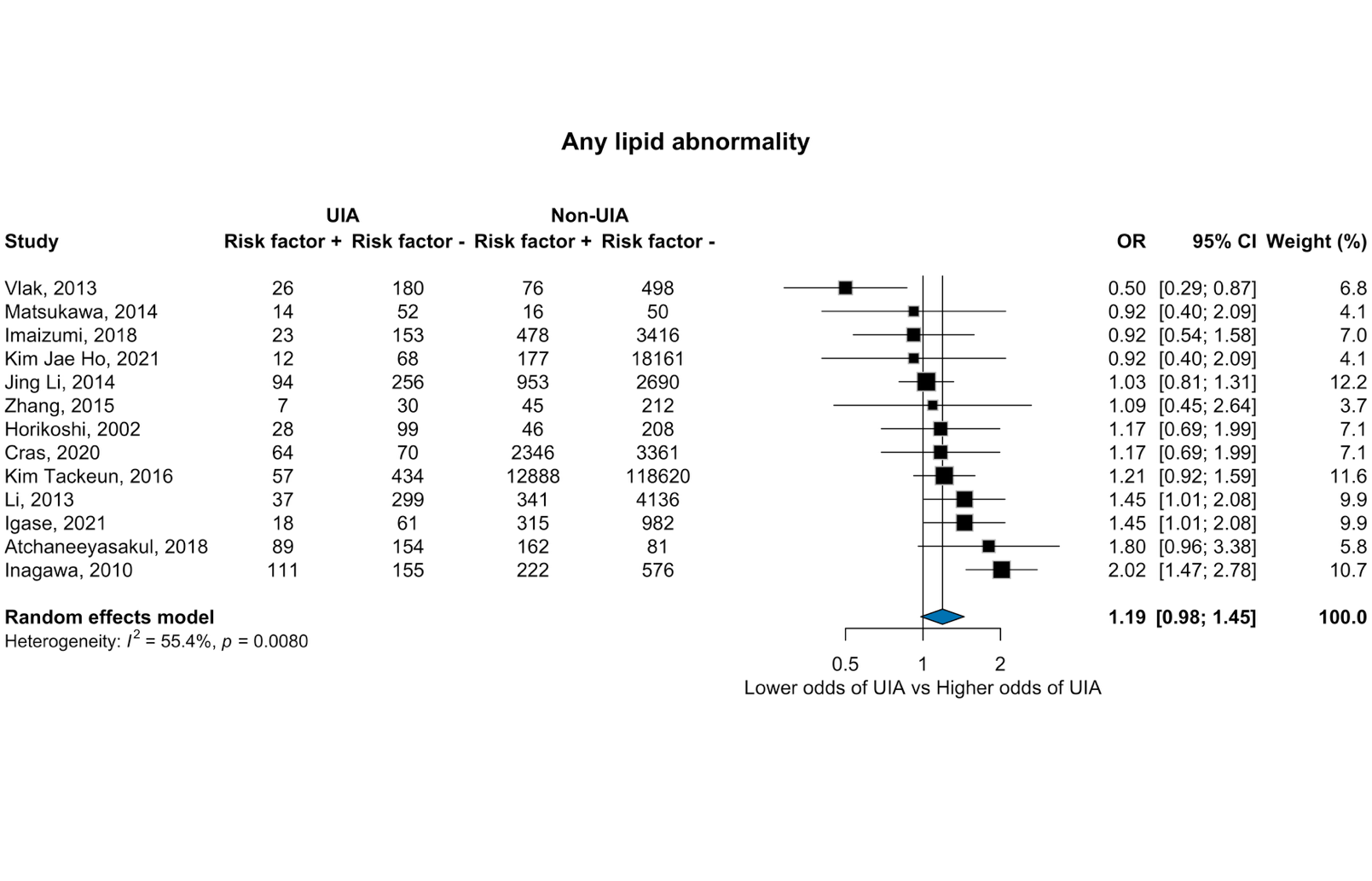
**

**S15. Forest plot of hypercholesterolemia**

**
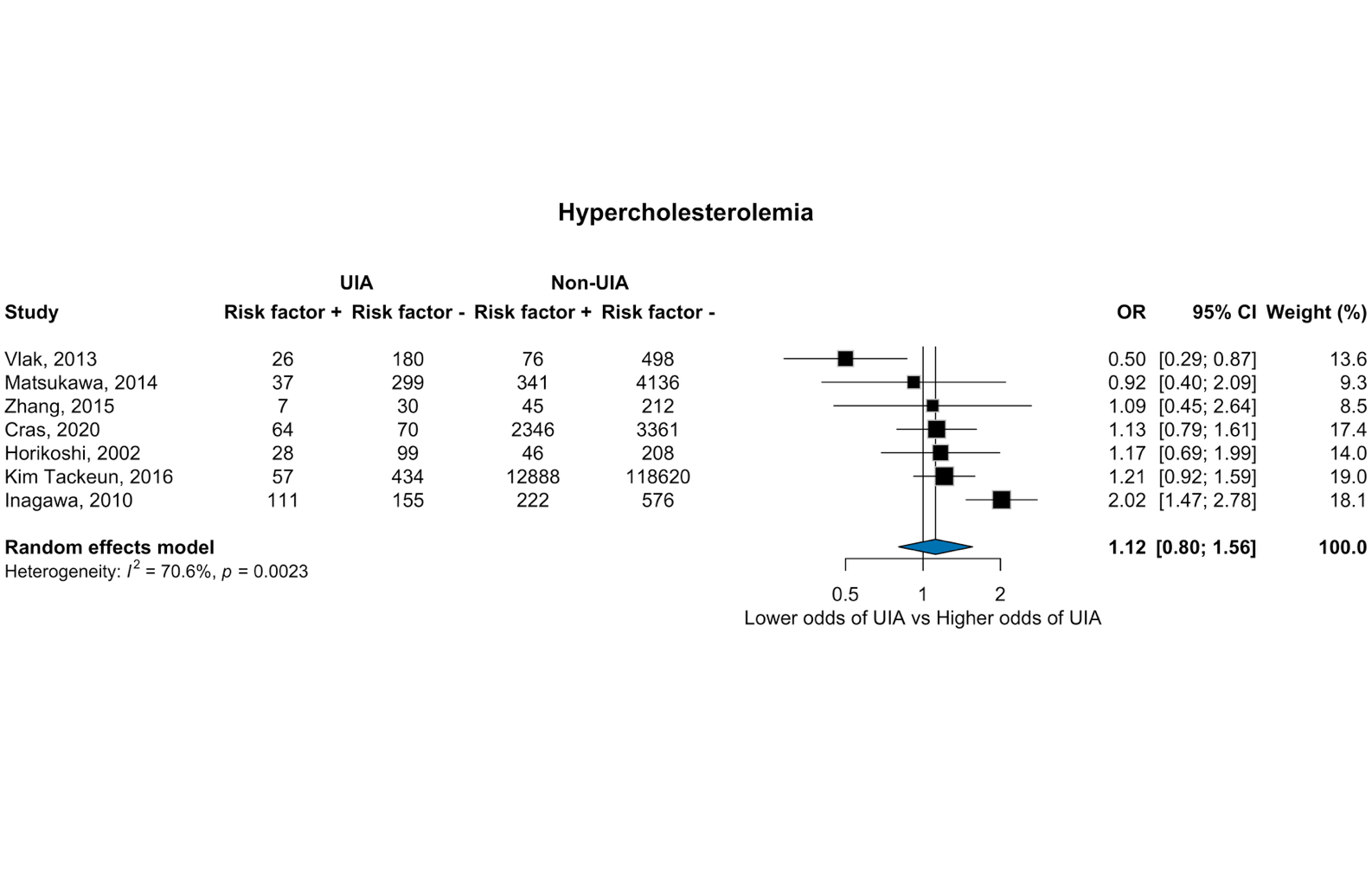
**

**S16. Forest plot of hyperlipidemia**

**
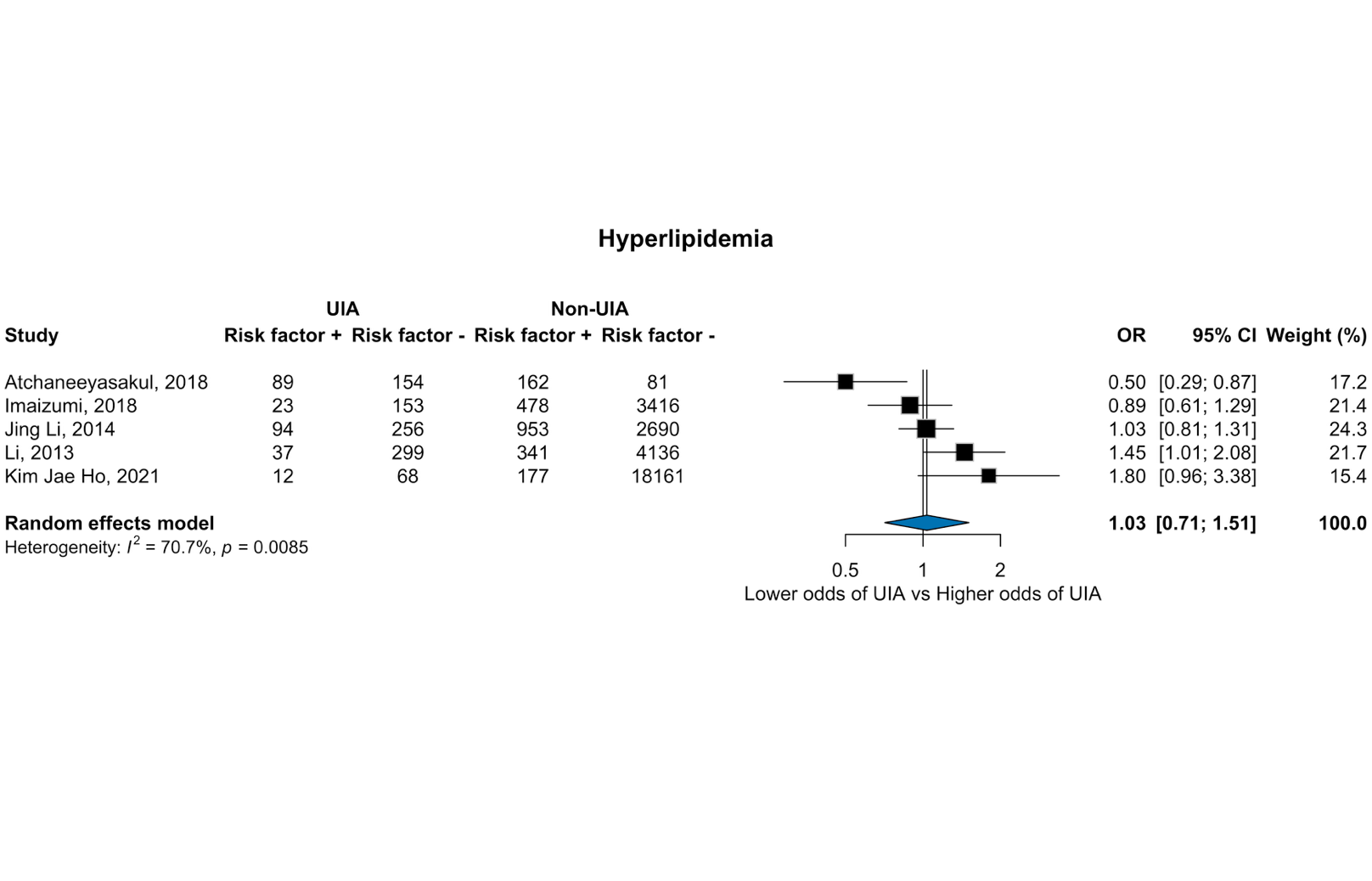
**

**S17. Forest plot of body mass index (BMI) (BMI ≥ 30 kg/m^2^ vs < 30 kg/m^2^)**

**
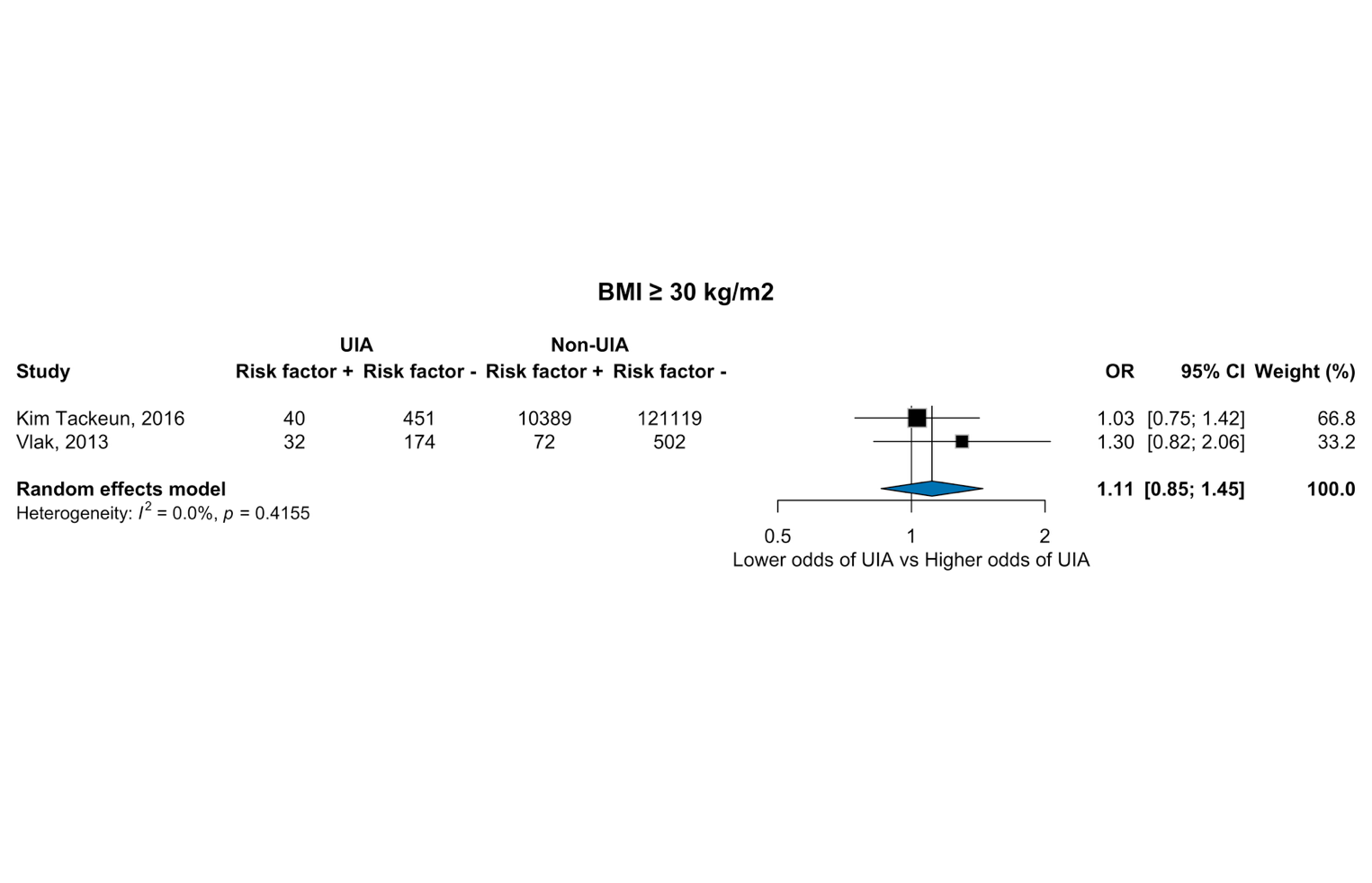
**

**S18. Forest plot of rigorous physical activity (≥ 3 times a week vs < 3 times a week)**

**
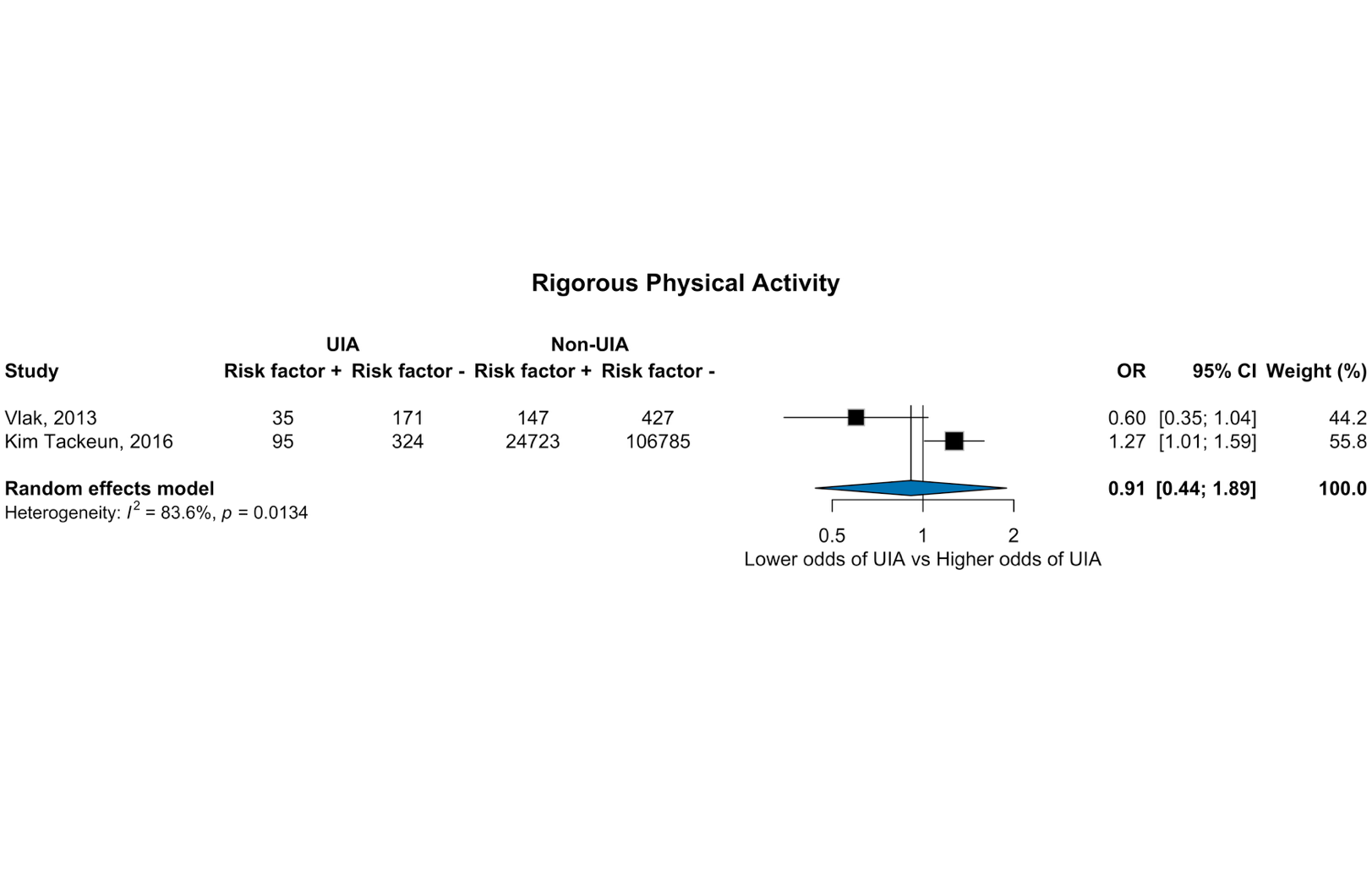
**

**S19. Subgroup analysis – Forest plot of hypertension (present vs absent)**

**
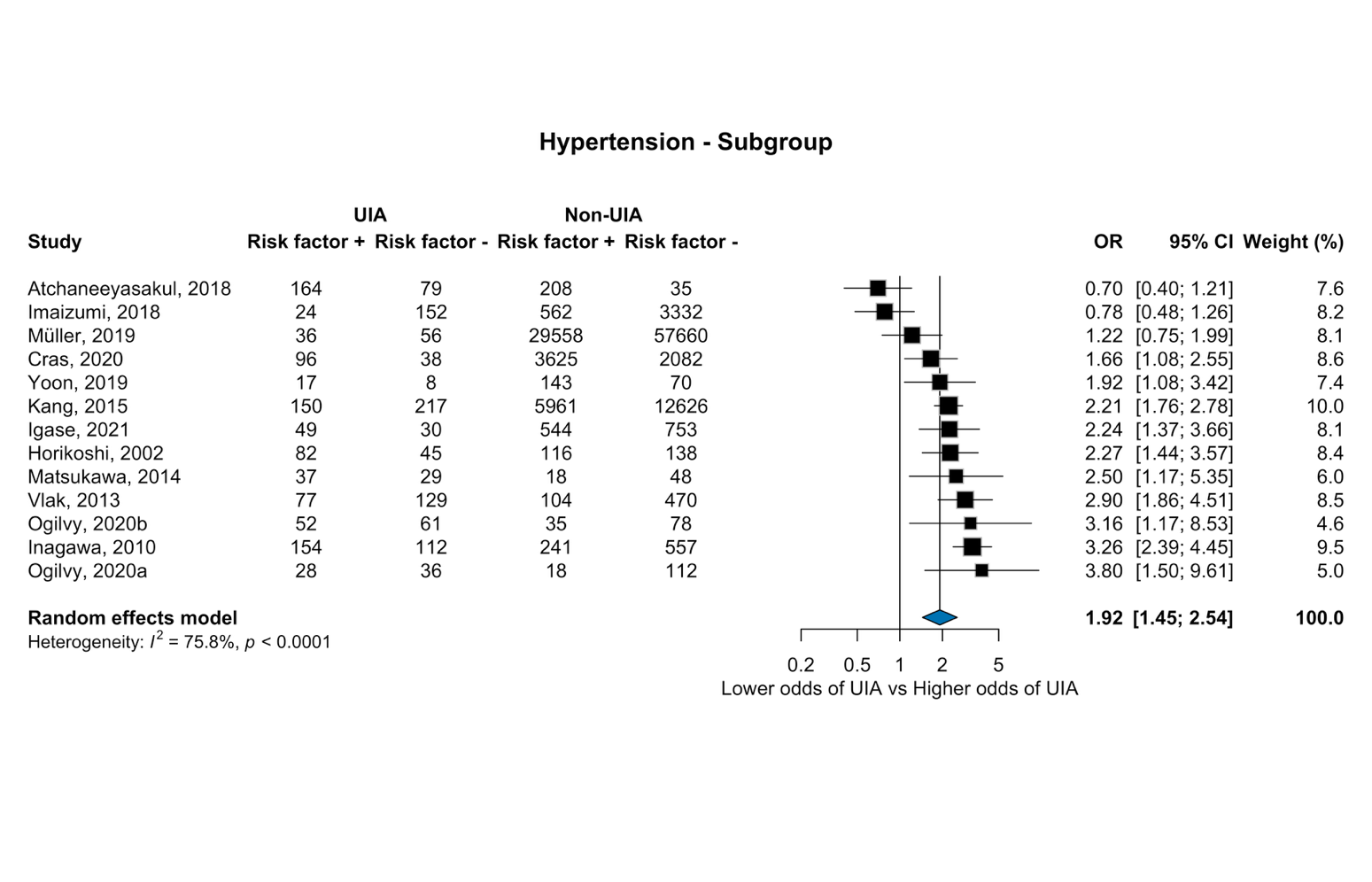
**

Ogilvy, 2020a refers to the single center study; Ogilvy, 2020b to the multicenter study.

**S20. Subgroup analysis – Forest plot of smoking (ever vs never)**

**
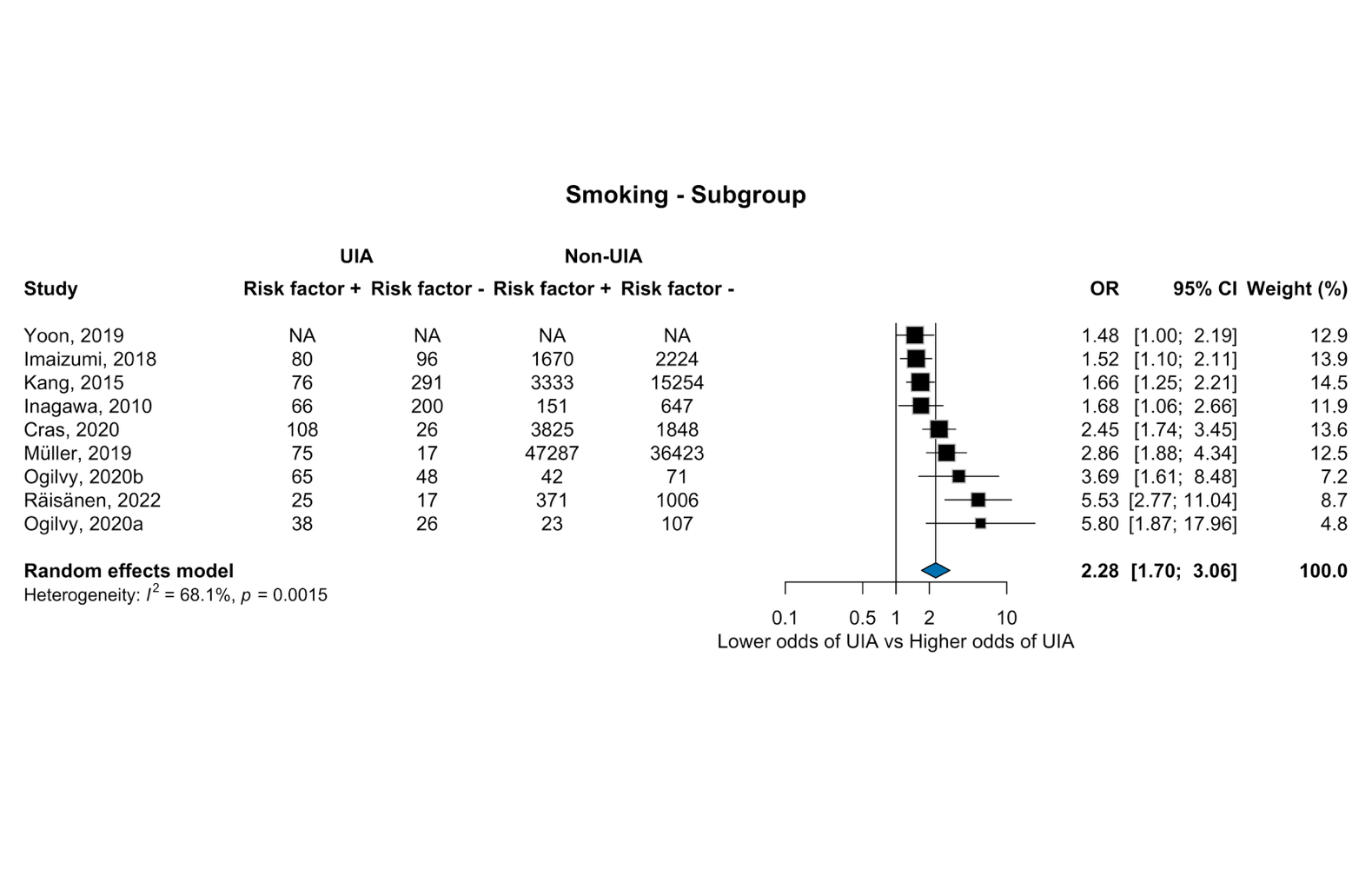
**

Ogilvy, 2020a refers to the single center study; Ogilvy, 2020b to the multicenter study.

NA = not available; the numbers of participants with and without the risk factor were not reported in the study. The study was still included in the forest plot using the reported odds ratio.

**
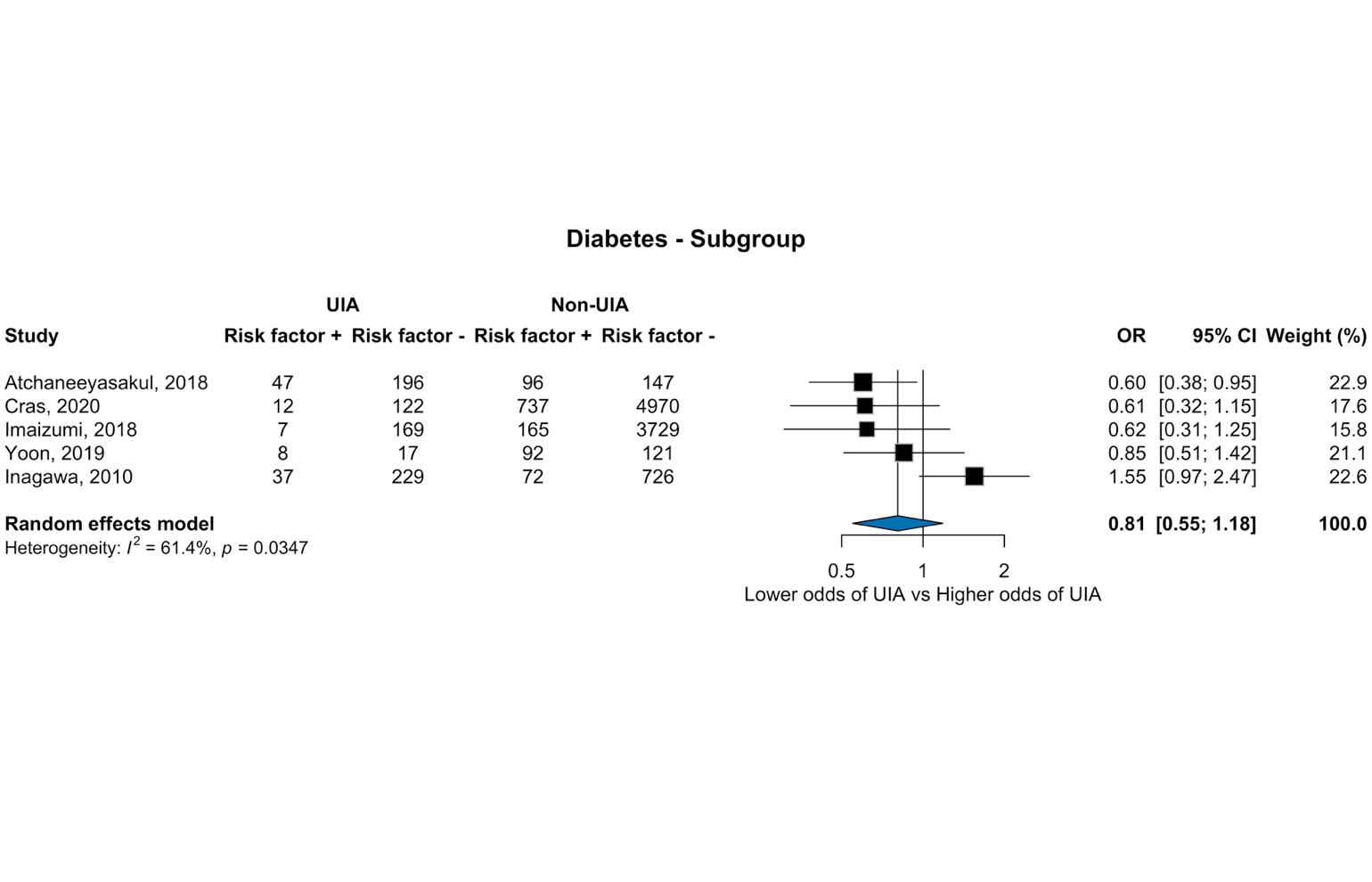
S21. Subgroup analysis – Forest plot of diabetes (present vs absent)**

**
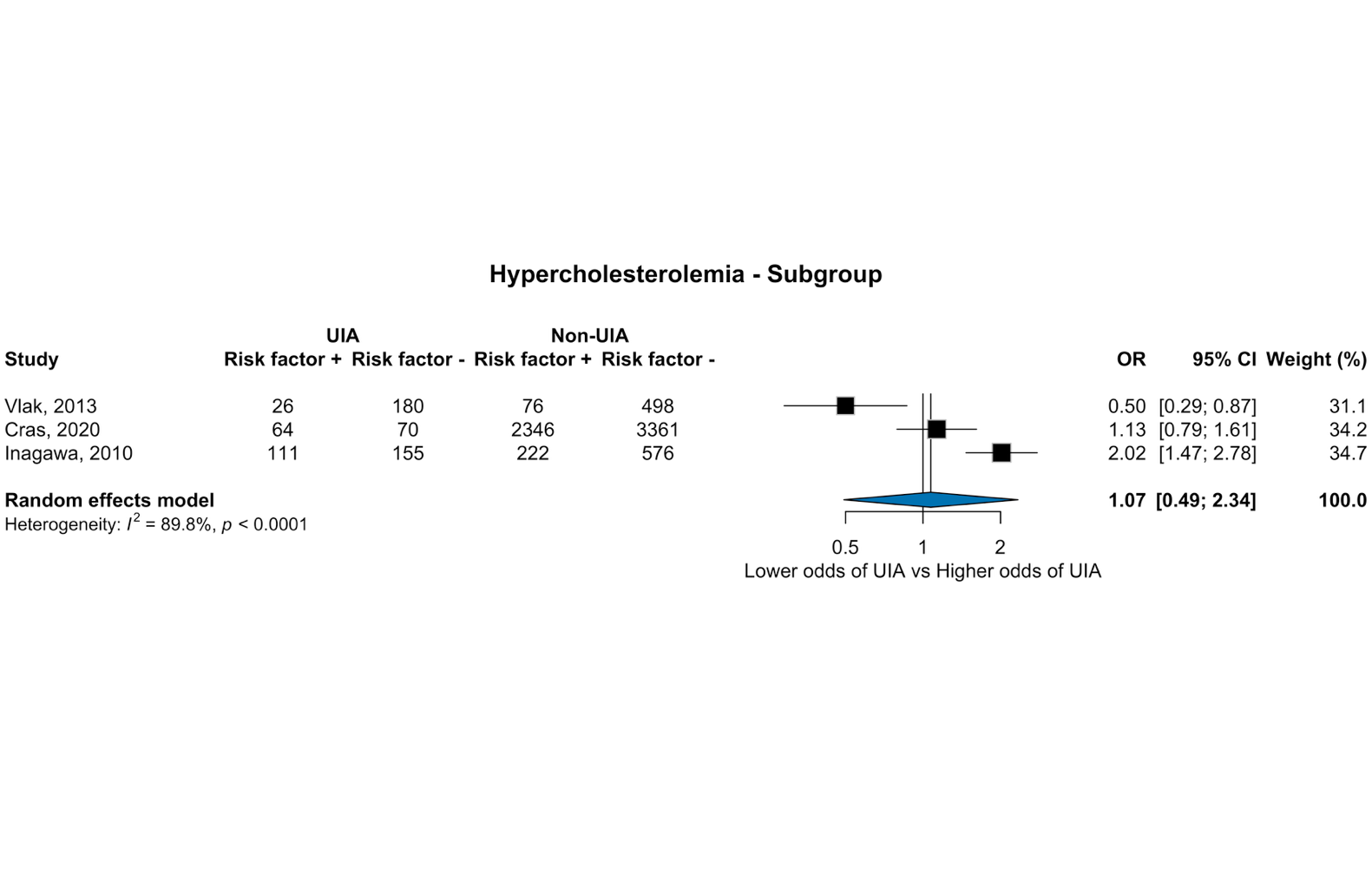
S22. Subgroup analysis – Forest plot of hypercholesterolemia (hypercholesterolemia vs normal cholesterol)**

**S23. References**

1. Wiebers DO, Whisnant JP, O’Fallon WM. The natural history of unruptured intracranial aneurysms. N Engl J Med. 1981;304(12):696–698.

2. de la Monte SM, Moore GW, Monk MA, Hutchins GM. Risk factors for the development and rupture of intracranial berry aneurysms. Am J Med. 1985;78(6 Pt 1):957–964.

3. Ohaegbulam SC, Dujovny M, Ausman JI, Diaz FG, Malik GM. Ethnic distribution of intracranial aneurysms. Acta Neurochir (Wien). 1990;106(3-4):132–135.

4. Juvela S, Poussa K, Porras M. Factors affecting formation and growth of intracranial aneurysms: a long-term follow-up study. Stroke. 2001;32(2):485–491.

5. Juvela S. Natural history of unruptured intracranial aneurysms: risks for aneurysm formation, growth, and rupture. Acta Neurochir Suppl. 2002;82:27–30.

6. Gu YX, Chen XC, Song DL, Leng B, Zhao F. Risk factors for intracranial aneurysm in a Chinese ethnic population. Chin Med J (Engl). 2006;119(16):1359–1364.

7. Nguyen TV, Chandrashekar K, Qin Z, Parent AD, Zhang J. Epidemiology of intracranial aneurysms of Mississippi: a 10-year (1997–2007) retrospective study. J Stroke Cerebrovasc Dis. 2009;18(5):374–380.

8. Marbacher S, Schläppi JA, Fung C, Hüsler J, Beck J, Raabe A. Do statins reduce the risk of aneurysm development? A case-control study. J Neurosurg. 2012;116(3):638–642.

9. Lindgren AE, Kurki MI, Riihinen A, Koivisto T, Ronkainen A, Rinne J, et al. Hypertension predisposes to the formation of saccular intracranial aneurysms in 467 unruptured and 1053 ruptured patients in Eastern Finland. Ann Med. 2014;46(3):169–176.

10. Lai LT, Morgan MK, Patel NJ. Smoking increases the risk of de novo intracranial aneurysms. World Neurosurg. 2014;82(1-2):e195–e201.

11. Wang JY, Smith R, Ye X, Yang W, Caplan JM, Radvany MG, et al. Serial imaging surveillance for patients with a history of intracranial aneurysm: risk of de novo aneurysm formation. Neurosurgery. 2015;77(1):32–43.

12. Guan J, Karsy M, Eli I, Bisson EF, McNally S, Taussky P, et al. Increased incidence of hypovitaminosis D among patients requiring treatment for cerebral aneurysms. World Neurosurg. 2016;88:15–20.

13. Lindgren AE, Räisänen S, Björkman J, Tattari H, Huttunen J, Huttunen T, et al. De novo aneurysm formation in carriers of saccular intracranial aneurysm disease in Eastern Finland. Stroke. 2016;47(5):1213–1218.

14. Duman E, Coven I, Yildirim E, Yilmaz C, Pinar HU, Ozdemir O. Association between brain venous drainage, cerebral aneurysm formation and aneurysm rupture. Turk Neurosurg. 2017;27(4):516–521.

15. Kim BJ, Lee SH, Kwun BD, Kang HG, Hong KS, Kang DW, et al. Intracranial aneurysm is associated with high intracranial artery tortuosity. World Neurosurg. 2018;112:e876–e880.

16. Hostettler IC, Alg VS, Shahi N, Jichi F, Bonner S, Walsh D, et al. Characteristics of unruptured compared to ruptured intracranial aneurysms: a multicenter case-control study. Neurosurgery. 2018;83(1):43–52.

17. Rosi J, Morais BA, Pecorino LS, Oliveira AR, Solla DJF, Teixeira MJ, et al. Hyperhomocysteinemia as a risk factor for intracranial aneurysms: a case-control study. World Neurosurg. 2018;119:e272–e275.

18. Krzyżewski RM, Kliś KM, Kucala R, Polak J, Kwinta BM, Starowicz-Filip A, et al. Intracranial aneurysm distribution and characteristics according to gender. Br J Neurosurg. 2018;32(5):541–543.

19. Vourla E, Filis A, Cornelius JF, Bostelmann R, Turowski B, Kalakoti P, et al. Natural history of de novo aneurysm formation in patients with treated aneurysmatic subarachnoid hemorrhage: a ten-year follow-up. World Neurosurg. 2019;122:e291–e295.

20. Wang Q, Zhang J, Zhao K, Xu B. Hyperhomocysteinemia is an independent risk factor for intracranial aneurysms: a case-control study in a Chinese Han population. Neurosurg Rev. 2020;43(4):1127–1134.

21. Schatlo B, Gautschi OP, Friedrich CM, Ebeling C, Jägersberg M, Kulcsár Z, et al. Association of single and multiple aneurysms with tobacco abuse: an @neurIST risk analysis. Neurosurg Focus. 2019;47(1):E9.

22. Júnior JR, Telles JPM, da Silva SA, Iglesio RF, Brigido MM, Pereira Caldas JGM, et al. Epidemiological analysis of 1404 patients with intracranial aneurysm followed in a single Brazilian institution. Surg Neurol Int. 2019;10:249.

23. Majewska P, Gulati S, Øie L, Salvesen Ø, Müller TB, Solheim O. Smoking habits and detection rate of unruptured intracranial aneurysms and incidence rate of subarachnoid haemorrhage in Norway between 2008 and 2015. Acta Neurochir (Wien). 2020;162(12):3161–3165.

24. Haase A, Schob S, Quäschling U, Hoffmann KT, Meixensberger J, Nestler U. Epidemiologic and anatomic aspects comparing incidental and ruptured intracranial aneurysms: a single centre experience. J Clin Neurosci. 2020;81:151–157.

25. Han HJ, Lee W, Kim J, Park KY, Park SK, Chung J, et al. Incidence rate and predictors of recurrent aneurysms after clipping: long-term follow-up study of survivors of subarachnoid hemorrhage. Neurosurg Rev. 2022;45(5):3209–3217..

26. Missori P, de Sousa DA, Ambrosone A, Currà A, Paolini S, Incarbone G, et al. Cotinine levels influence the risk of rupture of brain aneurysms. Acta Neurol Scand. 2022;146(5):562–567.

27. Morel S, Hostettler IC, Spinner GR, Bourcier R, Pera J, Meling TR, et al. Intracranial aneurysm classifier using phenotypic factors: an international pooled analysis. J Pers Med. 2022;12(9):1410.

28. Wahood W, Rizvi AA, Alexander AY, Yolcu YU, Lanzino G, Brinjikji W, et al. Trends in admissions and outcomes for treatment of aneurysmal subarachnoid hemorrhage in the United States. Neurocrit Care. 2022;37(1):209–218.

29. Wälchli T, Ndengera M, Constanthin PE, Bisschop J, Morel S, Gautschi O, et al. Sex-dependent manifestations of intracranial aneurysms. Stroke Vasc Interv Neurol. 2024;Epub ahead of print Jul 2024.

30. Javed K, Ahmad S, Qin J, Mowrey W, Kadaba D, Liriano G, et al. Higher incidence of unruptured intracranial aneurysms among Black and Hispanic women on screening MRA in large urban populations. AJNR Am J Neuroradiol. 2023;44(5):574–579.

31. Jung TY, Lee E, Park M, Lee JY, Hong YS, Cho J, et al. Obstructive sleep apnea and its influence on intracranial aneurysm. J Clin Med. 2023;13(1):144.

32. Zeng Y, Guo R, Cao S, Yang H. Impact of blood pressure and antihypertensive drug classes on intracranial aneurysm: a Mendelian randomization study. J Stroke Cerebrovasc Dis. 2023;32(11):107355
